# Insights from Theoretical Modeling of Cesium-Formamidinium-Based Mixed-Halide Perovskite Solar Cells for Outdoor and Indoor Applications

**DOI:** 10.1021/acsomega.4c06752

**Published:** 2024-11-04

**Authors:** David Mora-Herrera, Jorge Alberto Polito-Lucas, Mou Pal

**Affiliations:** † Ingeniería en Energía. Universidad Politécnica de Amozoc. Av. Ampliación, Luis Oropeza No. 5202 C.P., Amozoc 72980, Mexico; ‡ Ciudad Universitaria, Instituto de Física, BUAP, San Claudio Blvd. 18 Sur Col. y Av. San Manuel, C.P., Puebla 72570, Mexico

## Abstract

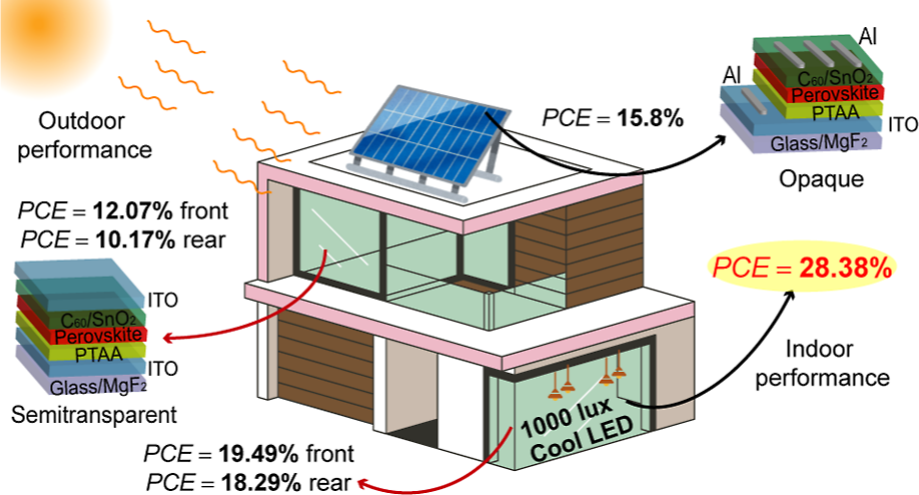

This study presents an in-depth computational analysis of hybrid organic–inorganic lead halide perovskite solar cells (PSCs) with a composition of Cs_0.17_FA_0.83_Pb(Br_0.4_I_0.6_)_3_ (FA: formamidinium) material under cool and warm light-emitting diodes (LEDs). We propose a novel design of an inverted (*p*-*i*-*n*) PSC to compare the power conversion efficiencies (PCEs) of opaque and semitransparent models under the AM1.5G spectrum and indoor LED lighting. The Shockley–Queisser (SQ) limits were estimated for LEDs with color temperatures of 3000 and 6000 K, revealing significant differences in PCE compared to standard solar radiation. The optical and electrical properties of the perovskite devices were simulated by using the transfer-matrix method and one-dimensional drift-diffusion model. We report a PCE of 15.8% for opaque devices under the AM1.5G spectrum, while the semitransparent devices exhibit PCEs of 12.07% and 10.17% for front and rear illumination, respectively. Under indoor conditions with cool LED lighting, the opaque devices demonstrate a significantly higher PCE of 28.38% and an impressive photovoltage of 1.17 V, surpassing the semitransparent devices, which show efficiencies of approximately 19.5% (front illumination) and 18.3% (rear illumination). While the interface between the hole transport layer and perovskite has a major impact on the device performance of opaque solar cells, the perovskite/electron transport layer junction plays a more critical role in the performance of semitransparent solar cells. The power densities for opaque devices reached up to 106.25 μW/cm^2^ under cool LED and 97.1 μW/cm^2^ with warm LED illumination. For semitransparent devices, the power densities exceeded 60.71 μW/cm^2^ on front-side illumination and 73.66 μW/cm^2^ on rear illumination under cool LEDs. These results emphasize the significant potential of hybrid PSCs for efficient energy harvesting under various lighting conditions, making them promising candidates for powering low-energy-consumption electronics in indoor environments.

## 1 Introduction

A viable approach to address the global energy crisis lies in the exploration of alternative clean energy sources.^1^ Solar energy stands out as one of the most powerful renewable resources because of being free of cost and due to its abundance, renewability, and environmental friendliness. By mentioning the term “solar cell”, a vast majority of the population associates it with the conversion of sunlight into electricity. However, the solar cells convert the optical power input coming from the sun or any artificial light source to electrical power output using the correct semiconductor material with photovoltaic properties.^2^ Besides traditional solar panels, indoor photovoltaics (IPVs) are gaining attention for their ability to harness photon energy utilizing household illumination or surrounding ambient.^3−5^

Nowadays, light-emitting diodes (LEDs) are becoming popular for indoor and outdoor lightning due to several advantages including less energy consumption (up to mW), longer durability, faster switching, and robustness.^5^ It is undoubtedly a brilliant idea if the emitted radiation of IPVs can be utilized for harvesting energy to power low-energy consumable electronics in addition to satisfying the prime purpose of indoor illumination.^6−9^ An IPV system enables the development of self-powered electronic devices for the Internet of Things (IoT) by stabilizing voltage fluctuations. In fact, photovoltaic (PV) technology has evolved from outdoor to indoor applications, with increased demand driven by long-term stability and higher photo conversion efficiency. In recent years, a large number of electronic devices are operated by IoT with wireless Internet connection and other low-power communication technologies like radio frequency, passive radio frequency identification, and wireless body area networks.^10−12^ In the emerging era of IoT, radio technology has introduced fresh demands regarding size, weight, energy consumption, and cost reduction.^12^ Various solar cell technologies have been explored for indoor applications starting with conventional PV types like Si,^13,14^ a-Si,^15^ GaAs,^16−18^ and GaInP,^19^ until PV options such as hybrid perovskite solar cells (PSCs),^20,21^ dye-sensitized solar cells,^22^ and organic PVs.^23^ Notably, PSCs hold significant promise for achieving high efficiency in indoor devices owing to their advantageous optoelectronic properties. These include a tunable band gap ranging from 1.2 eV to about 3.5 eV, high optical absorption coefficients (10^5^ cm^–1^), small exciton binding energy (less than 100 meV), and long carrier diffusion lengths over 1 μm.^20,21^ Recently, PSCs have reached a significant milestone, achieving a PCE exceeding 25% under AM1.5G illumination. This surpasses the efficiency levels of other mature PV technologies such as Cu(In,Ga)Se_2_, CdTe, Si, and a-Si.^24^

Over the past few years, there has been significant interest in organometallic halide-type hybrid perovskites in the field of next-generation solar cell research. This interest stems from the exceptional performance of PSCs, which depends strongly on the morphology and type of materials used as a hole or electron transport layer.^24^ The high efficiency of hybrid perovskites is attributed to their large photocurrent, photovoltage, and fill factor (FF) along with their ability to absorb light across the visible spectrum. The promising performance of perovskites highlights their potential as an ideal candidate for indoor light-harvesting applications.^20,21^ Despite their outstanding performance under 1 Sun conditions, there is a lack of comprehensive research on the fundamental mechanisms of power generation in perovskite materials under low-intensity illumination. The pioneering research on perovskite IPVs was carried out by Kawata et al. in 2015, showing a remarkable 19.8% PCE at 1000 lx under simulated indoor conditions.^25^ Within a few years, the indoor PCE of perovskite cells has exceeded 40% under the same 1000 lx illumination.^26^ Additionally, studies have shown that PSCs achieve a higher PCE under LED illumination compared to the standard AM1.5G spectrum. This is primarily attributed to the enhanced spectral overlap between indoor lighting and the absorbance spectrum of the solar cells.

In this paper, we propose a novel design for an inverted PSC and compare the conversion efficiency of IPVs in opaque and semitransparent configurations using the AM1.5G spectrum and LED illumination in a simulation environment. We begin with the estimation of the classic Shockley–Queisser (SQ) limits in order to define the ideal performance of solar cells under artificial light sources. At first instance, we predicted the performance limits of SQ using LEDs with different color temperatures of 3000 and 6000 K. Our calculations revealed an interesting finding that the dependence of solar efficiency on energy band gap varies between indoor lighting and standard solar radiation. The optical properties of the semiconductors were deduced by the transfer-matrix method (TMM) to model the inverted PSC using a refractive index database collected from experimental findings and existing literature. Our simulations aim to explore the potential of opaque and semitransparent perovskite devices by utilizing efficient electron and hole transport layers (ETL and HTL). We consider the optical characteristics of each layer and examine how their combinations affect the PCE of the final devices. The obtained results reveal that PSCs with a composition of Cs_0.17_FA_0.83_Pb(Br_0.4_I_0.6_)_3_ (FA, formamidinium)^27^ exhibit 15.8% of efficiency for opaque solar cell against the PCEs of 12.07% and 10.17% under front and rear illumination of the semitransparent device, respectively, utilizing the AM1.5G spectrum. It has been found that IPVs achieve greater efficiency with ambient illumination from cool LEDs than with outdoor light. The simulation results demonstrate that the opaque devices exhibit superior performance, yielding an outstanding PCE of 28.38% and a high photovoltage of 1.17 V in comparison to 19.49% and 18.29% PCEs for the semitransparent device by illuminating front and rear surfaces, respectively. Additionally, the power densities reached 106.25 and 97.1 μW/cm^2^ for the opaque solar cell under cool and warm LED lamps, respectively. For the semitransparent device, the power densities exceeded 60.71 (63.16) and 69.53 (73.66) μW/cm^2^ under warm and cool LED lamp with front (rear) illumination, respectively, which are significantly high for indoor low-intensity light solar cells reported so far.^20,21^ The present research offers a reliable and detailed methodology for evaluating the efficiency of IPVs in a realistic way and provides valuable insights for selecting the appropriate materials to enhance their performance.

## 2 Device Model

### 2.1 Parameters for IPVs and SQ Limit for Artificial Light Sources

**Figure 1:**
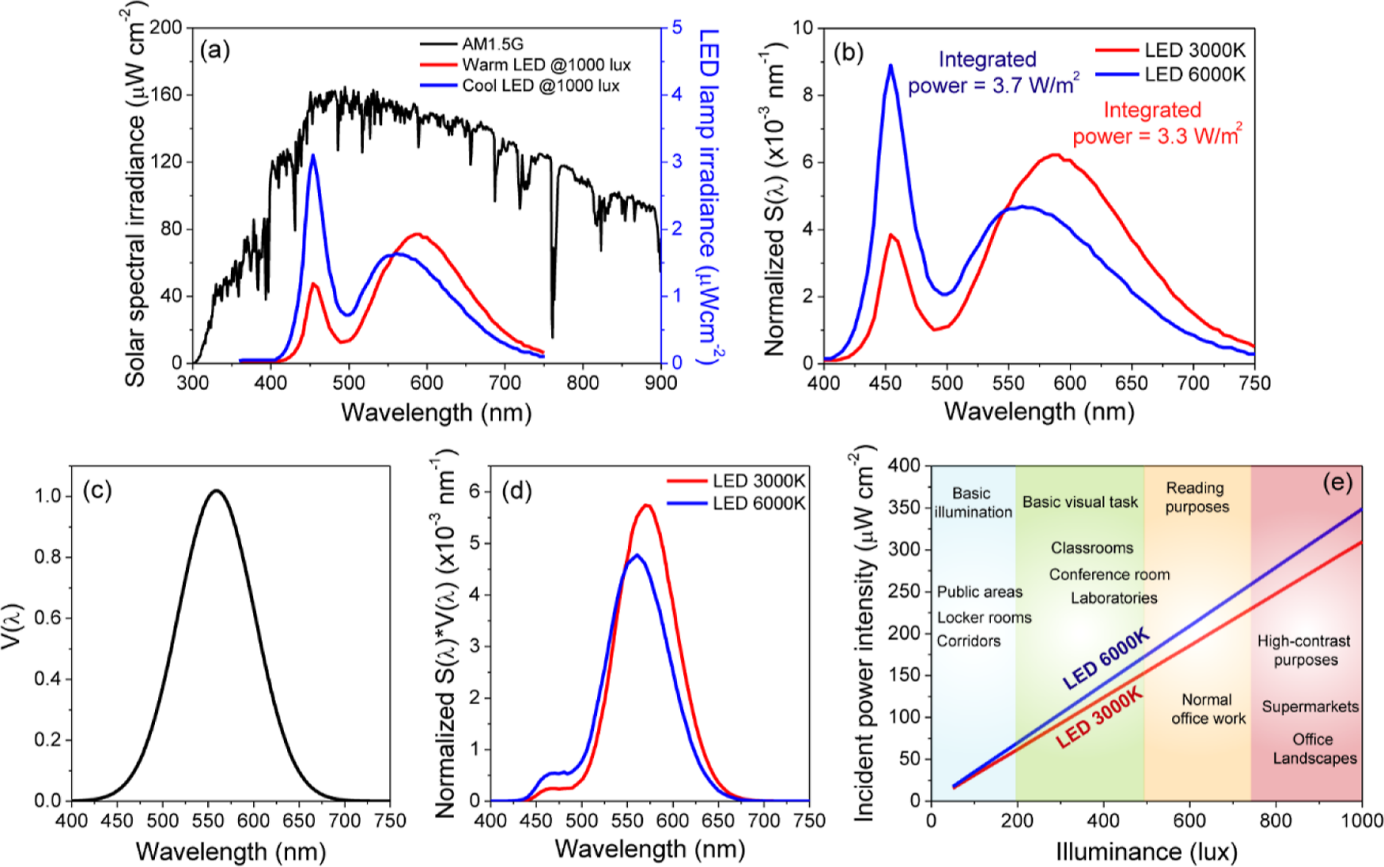
(a) Spectra of different light sources: AM1.5G and LED lamps with different color temperatures. (b) Emission spectra of two LED sources. (c) Human photopic luminous efficiency function. (d) Distribution of normalized *S*(λ)× *V*(λ). (e) Incident power intensity *P*_in_ of selected indoor light sources as a function of illuminance.

An Ocean Optics USB2000 miniature fiber optic spectrometer was utilized to measure the spectra of two artificial lights with varying color temperatures. This methodology was described in an article by Popular Mechanics^28^ and confirmed by Theremino spectrometer.^29^Figure 1a shows the emission spectra of artificial LED bulbs, which were recorded at a brightness of 1000 lx and compared with the standard AM1.5G solar spectrum. The LED light sources had color temperatures of 3000 and 6000 K for warm and cool lights with integrated power densities of 3.3 and 3.7 W/m^2^, respectively. We have chosen these specific color temperatures as they are commonly used for indoor and outdoor lighting. The warm color temperature of 3000 K is often used in residential settings or cozy environments, while the cool color temperature of 6000 K is similar to daylight and commonly utilized in offices, commercial spaces, and areas where higher visual clarity is required. It is noteworthy that the power generated by cool LEDs was slightly higher than that produced by warm LED bulbs, likely due to the increased blue light emission from the cool LEDs. Furthermore, the total energy emitted by LEDs at 1000 lx was approximately 2 orders of magnitude lower than that emitted by 1 Sun illumination. The light sources were analyzed to obtain emission spectra *S*(λ). Figure 1b shows the normalized LED emission spectra using the total emission intensity defined as ∫*S*(λ)dλ. As a result, the area under each curve is equal to one. The cool LED exhibits stronger optical emission in the near-ultraviolet region with a peak at 450 nm, while the warm LED shows higher emission in the visible and near-infrared regions with a maximum at 600 nm. The increase in the color temperature typically results in higher blue emission, producing a cooler color tone. To determine the input power intensities of the LED sources, illuminance was used as the basis of the calculation. Two units were considered to evaluate natural and artificial light sources: radiometric and photometric units. Radiometric measurements refer to the total power across the entire spectrum, whereas photometric measurements are expressed in lux. The relationship between illuminance *L* and incident power intensity *P*_in_ can be mathematically expressed by eq 1
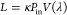
1where κ is the maximum spectral luminous efficacy for human photopic vision equal to 683.002 lm/W^30−32^ and *V*(λ) is the color matching function as defined by the International Commission on Illumination (CIE) 1931 2° (standard colorimetric observer for 1–4° field of view) and represents the average spectral sensitivity of human eye in the photopic vision regime.^9^ In order to make conversions between radiometric and photometric units, it is necessary to utilize the photopic spectral luminous efficiency curve *V*(λ), which is shown in Figure 1c. This curve reveals the sensitivity of the human eye to different wavelengths of light and reaches its highest value of unity at 555 nm, where the human eye is the most responsive. To determine the total illuminance *L*, the integral of the emission spectrum for photopic vision, represented as *S*(λ) × *V*(λ), must be calculated over the visible region as defined by the following eq 2
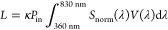
2where .

The graphical representation in Figure 1d exhibits the distribution of the normalized products *S*(λ) and *V*(λ). By utilizing eq 2, we are able to compute the power of irradiance, *P*_in_, for different light sources as a function of illuminance, *L*. Figure 1e illustrates the incident power intensity (*P*_in_) of indoor light sources as a function of illuminance (lux). At lower illumination levels (∼200 lx), the *P*_in_ is roughly 50 μW/cm^2^, while under brighter conditions (1000 lx), the *P*_in_ increases significantly to around 300 μW/cm^2^.

Using the emission spectra of two LED sources, we can determine the proportion of emitted photons that could be absorbed by the perovskite as a function of the band gap energy. It is assumed that photons with an energy exceeding the band gap are completely absorbed. To explain this phenomenon, we utilized the SQ limit, following the same methodology outlined by Rühle.^33^ Initially, we determined the maximum photocurrent density (*J*_max_), converting all spectral irradiance of light sources into an incident spectral photon flux using eq 3
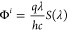
3where *q* is the elemental charge, λ is the vacuum wavelength, *h* is Planck’s constant, *c* is the speed of light in vacuum, and *S*(λ) is the emission spectra for different light sources. The *J*_max_ was calculated by integrating the spectral photon flux of *S*(λ) irradiance with the help of eq 4
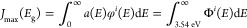
4

An energy of 3.54 eV corresponds to a wavelength of 350 nm, which is the shortest wavelength considered in this study, based on the type of light sources used. The model calculation in this work focuses on a single *p*–*n* junction cell exposed to unconcentrated or diffuse illumination. In this scenario, when a photon with energy greater than the band gap (*E*_g_) interacts with the cell, it generates one electron–hole pair. This process can be represented by a Heaviside step function, where the value is one when the photon energy is equal to or greater than *E*_g_.

Using the generalized Planck’s law of thermal radiation and Kirchhoff’s law for surface emission from a body or solar cell at different temperatures, the emitted photon flux *F* originating from the radiative recombination can be expressed as a function of an external applied voltage by eq 5 under the conditions of flat quasi-Fermi levels
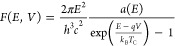
5where *E* is the photon energy, *V* is the external applied voltage, *k*_*B*_ is the Boltzmann constant, and *T*_C_ is the temperature of the cell. The recombination current density was calculated according to the relation (eq 6)
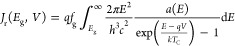
6

The factor *f*_g_ is a geometrical factor as reported by SQ^33^ based on the assumption that the solar cell is emitting radiation from the front and rear sides (*f*_g_ = 2).

Given that each photon absorbed by the cell generates one electron–hole pair, the short circuit current density (*J*_SC_) can be defined as the current produced by the overall photon flow minus the current loss due to recombination (eq 7)
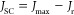
7

The *V*_OC_ was calculated according to eq 8
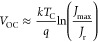
8In ideal devices, where shunt and series resistances are negligible, FF can be written using Green’s empirical formula 9
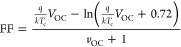
9

To estimate the SQ limits for different light sources, the maximum PCE can be calculated by eq 10
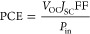
10

**Figure 2:**
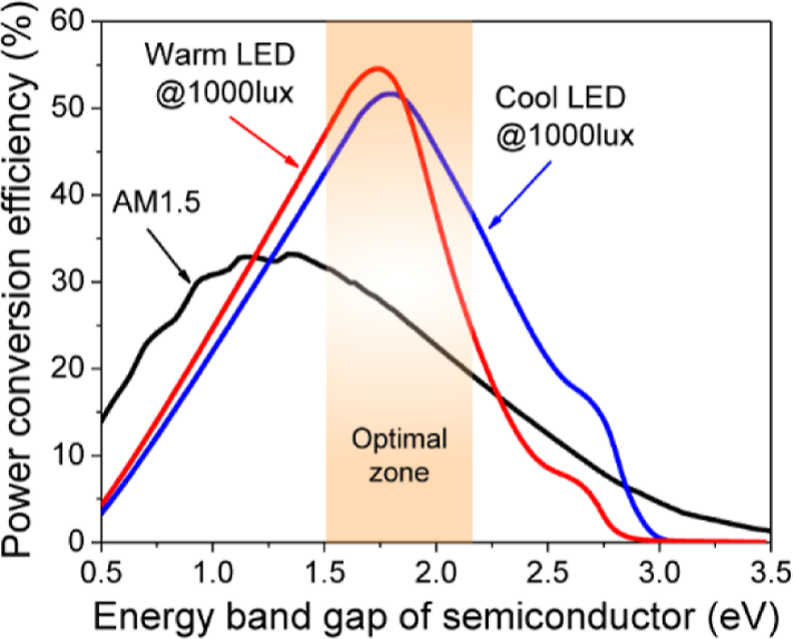
Maximum PCE as a function of band gap energy for the AM1.5G spectrum, as well as for warm and cool LED sources at 1000 lx.

Using this step-by-step approach, we calculated the efficiencies of two indoor light sources for different band gap values. The results presented in Figure 2 were compared to the SQ limit calculated for the standard solar spectrum under 1 Sun conditions. The warm LED achieved a maximum SQ limit of 54% with a band gap of 1.72 eV, while the cool LED attained a maximum of 51% with a band gap of 1.78 eV. These values are significantly higher than the maximum SQ limit of 33% for the AM1.5G solar irradiation spectrum. It was found that artificial light sources emit only a limited number of photons with wavelengths >620 nm, which explains the higher efficiency of artificial lighting compared to the AM1.5G spectrum. It is evident from Figure 2 that there is an optimal range of band gap in which the solar cell exhibits an improved PCE and the efficiency trend for indoor lighting sources differs from that observed under solar irradiation. Based on theoretical calculations, we conclude that the optimal band gap for highly efficient IPVs lies between 1.7 and 1.8 eV.

### 2.2 Optical Modeling

**Figure 3:**
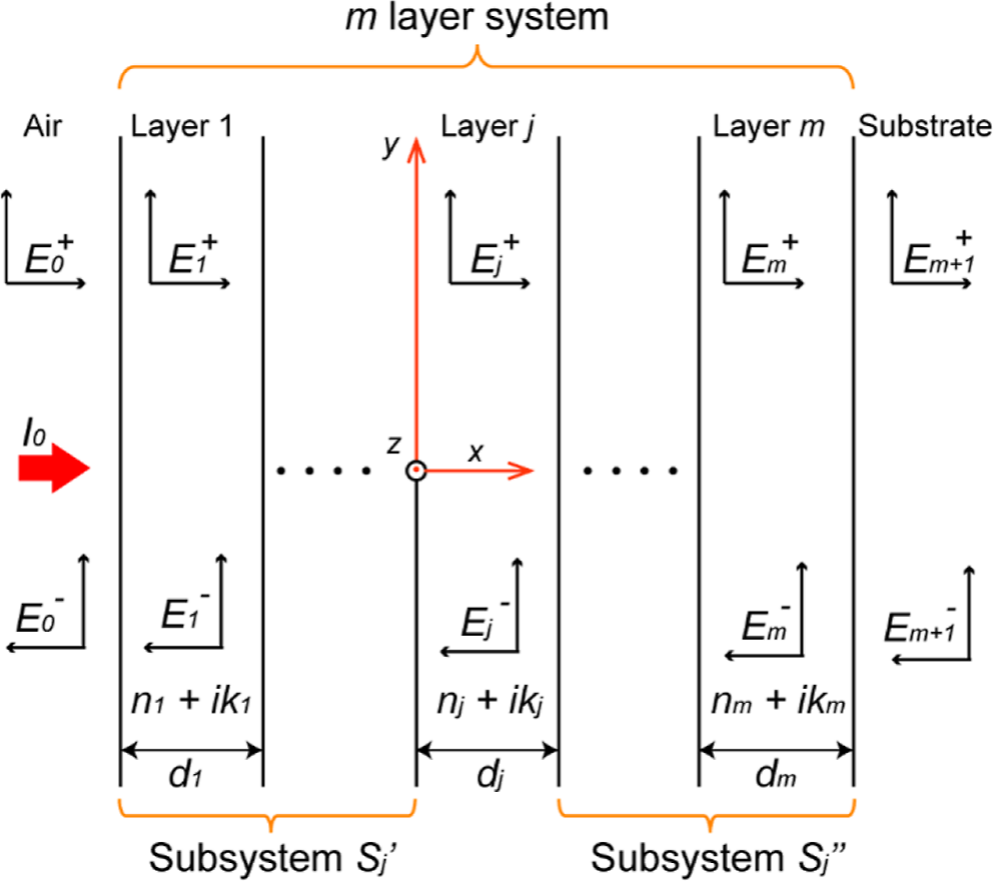
Schematic diagram of a multilayer device consisting of *m* layers between air and the substrate. The optical electrical field at any point is composed of two components.

The TMM is a powerful mathematical tool that considers the complex nature of the wave vector and studies multiple reflections and interference phenomena of the forward and backward propagating waves. The resulting electromagnetic radiation obtained by TMM can be used in the semiconductor continuity equation to determine the photocarrier generation rate. Its primary purpose is to analyze the reflection and transmission properties of plane waves within an infinite slab made of linear materials.^34,35^ In the context of modeling PSCs, TMM has been utilized to predict the optical characteristics of the material of each layer and their impact on the overall efficiency of the devices. Typically, the analysis considers a light ray incident perpendicularly on the surface. In this case, we have considered light approaching from the left side of a multilayer structure consisting of *m* layers, situated between air and a substrate (Figure 3). When light travels through a specific layer, suppose layer *j*, its velocity changes and a portion of the light is lost due to interference within the structure at any given point *x* in *j* layer. This interference results in an optical electric field composed of a positive component *E*_*j*_^+^(*x*) and a negative component *E*_*j*_^–^(*x*). To address this issue, the complex refractive index () is utilized. This parameter is defined as , where *n*_*j*_ is the refractive index and κ_*j*_ represents the extinction coefficient of the material. It is important to note that these quantities depend on the wavelength of the incident light. Additionally, the thickness (*d*_*j*_ for layer *j*) of each layer plays a crucial role in describing the optical properties of the layer.

Two matrices are commonly employed to establish optical transfer matrices. The interface matrix (*I*_*jk*_) describes each interface, while the layer matrix (*L*_*J*_) illustrates the propagation of light through a specific layer. When light travels from one layer to the adjacent one, it undergoes refraction and reflection. These phenomena can be accurately described using a 2 × 2 interface matrix, which is commonly known as the matrix of refraction. Thus, for two neighboring layers, the interface matrix *I*_*jk*_ at normal incidence can be defined by eq 11
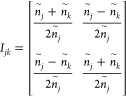
11When light passes through different layers, part of it is absorbed by each layer and can be represented by a 2 × 2 sized phase matrix. For any layer (e.g., layer *j*), the layer matrix *L*_*j*_ at normal incidence is given by eq 12
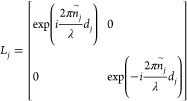
12

To proceed further, it is necessary to calculate the overall transfer matrix *S*, obtained by multiplying all of the individual transfer matrices defined in the device model. This allows us to establish a correlation between the optical electric field in the zeroth layer and that in the (*m* + 1)^th^ layer, as expressed in eq 13
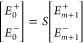
13where *S* is the scattering matrix, defined as eq 14
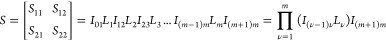
14

The general equation that describes the optical electric field distribution in the device at layer *j* is expressed as eq 15
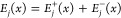
15where *E*_*j*_^+^(*x*) and *E*_*j*_^–^(*x*) are calculated by solving the matrix in eq 13 using iterative methods based on the transfer matrix described in eq 14.

The electric field intensity calculated by TMM can be expressed by eq 16
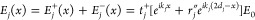
16where, *E*_0_ corresponds to the electric field of incident light from the ambient and *t*_*j*_^+^ and *r*_*j*_^″^ are the complex transmission and reflection coefficients for the layer *j* of the device, which can be defined by the eqs 17 and 18, respectively
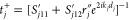
17
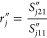
18*S*_*j*_^′^ and *S*_*j*_^″^ are partial transfer matrices obtained by subtracting the components from the transfer matrix *S* in eq 14. These subsystems are defined by eqs 19 and 20.
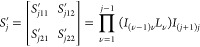
19
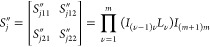
20

By rearranging the variables in eq 20 and substituting the expressions for *E*_*j*_(*x*) and light intensity *I*_*j*_(*x*) within the active layer, we obtained the following expression (eq 21) in a desired form that helps to establish the definition of *E*_*j*_(*x*) based on the constituent components of subsystem matrices.
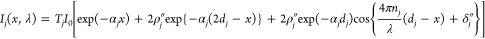
21where  represents the internal transmittance intensity, *n*_*j*_ is the refractive index of layer *j*, *n*_0_ is the refractive index of air, *t*_*j*_^+^ is the internal transfer coefficient for forward propagation within the layer, *I*_0_ is the incident light intensity (irradiance),  is the absorption coefficient for layer *j*, κ_*j*_ is the extinction coefficient of layer *j*, ρ_*j*_^″^ and δ_*j*_^″^ are the magnitude and the angle of the complex refraction coefficient (*r*_*j*_^″^) defined from *S*_*j*_^″^, respectively, λ is the wavelength, and *d*_*j*_ is the thickness of layer *j*.

Multiplying *I*_*j*_(*x*,λ) by the absorption coefficient α_*j*_ gives the power dissipation , which will help to calculate the excitation generation rate (*G*) in the active layer as shown below in eq 22
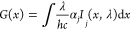
22where *h* is the Planck constant and *c* is the speed of the light in vacuum.

The TMM is employed to obtain the reflectance, transmittance, and absorbance spectra of the layers. In this case, we utilized a MATLAB/R19 script to calculate absorption and optical intensity in multilayer stacks. Additionally, we evaluated the charge carrier generation throughout the absorber layer. The models used in this simulation are the same as those utilized in the ellipsometry fitting process. The optical constants and complex refractive index spectra of the materials are collected from the previously reported experimental data^27,36−41^ (Figure S1 of Supporting Information).

### 2.3 Electrical Modeling

The *JV* characteristics of a simulated solar cell can be obtained by applying one-dimensional (1D) drift-diffusion equations for both electrons and holes and on solving the Poisson equation in 1D with finite boundary conditions using a MATLAB/R19 script.^42,43^ In this particular scenario, we assume that the rate of charge carrier generation depends on the absorption profile throughout the active layer when the device is illuminated.^33,34^ To calculate this, we utilize the TMM formalism, as described previously. When the active layer of the solar cell is exposed to light, both excitons and free carriers are created. Since the exciton binding energy is low (of the order of a few meV), we can reasonably assume that the generation of free charge carriers occurs almost instantaneously after light absorption in the active layer. Consequently, the equation that describes the transport of free charges is governed by both diffusion- and electric-field-induced drift for electrons and holes (eqs 23 and 24).
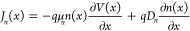
23
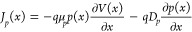
24where *J*_*n*_ and *J*_*p*_ are electron and hole current densities, respectively, μ_*n*_ and μ_*p*_ are electron and hole mobilities, respectively, *q* is the electronic charge, *V*(*x*) is the electrostatic potential, *n*(*x*) and *p*(*x*) are electron and hole concentrations, respectively, and *D*_*n*_ and *D*_*p*_ are electron and hole diffusion constants, respectively. The diffusion constant obeys the Einstein relation according to eq 25
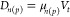
25where *V*_*t*_ is thermal voltage defined as , *T* is the absolute temperature, and *k*_*B*_ is Boltzmann’s constant.

The Poisson equation relates the electron and hole densities to the electric potential given by eq 26
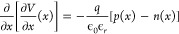
26where ϵ_0_ is the permittivity of free space and ϵ_*r*_ is the relative permittivity.

**Figure 4:**
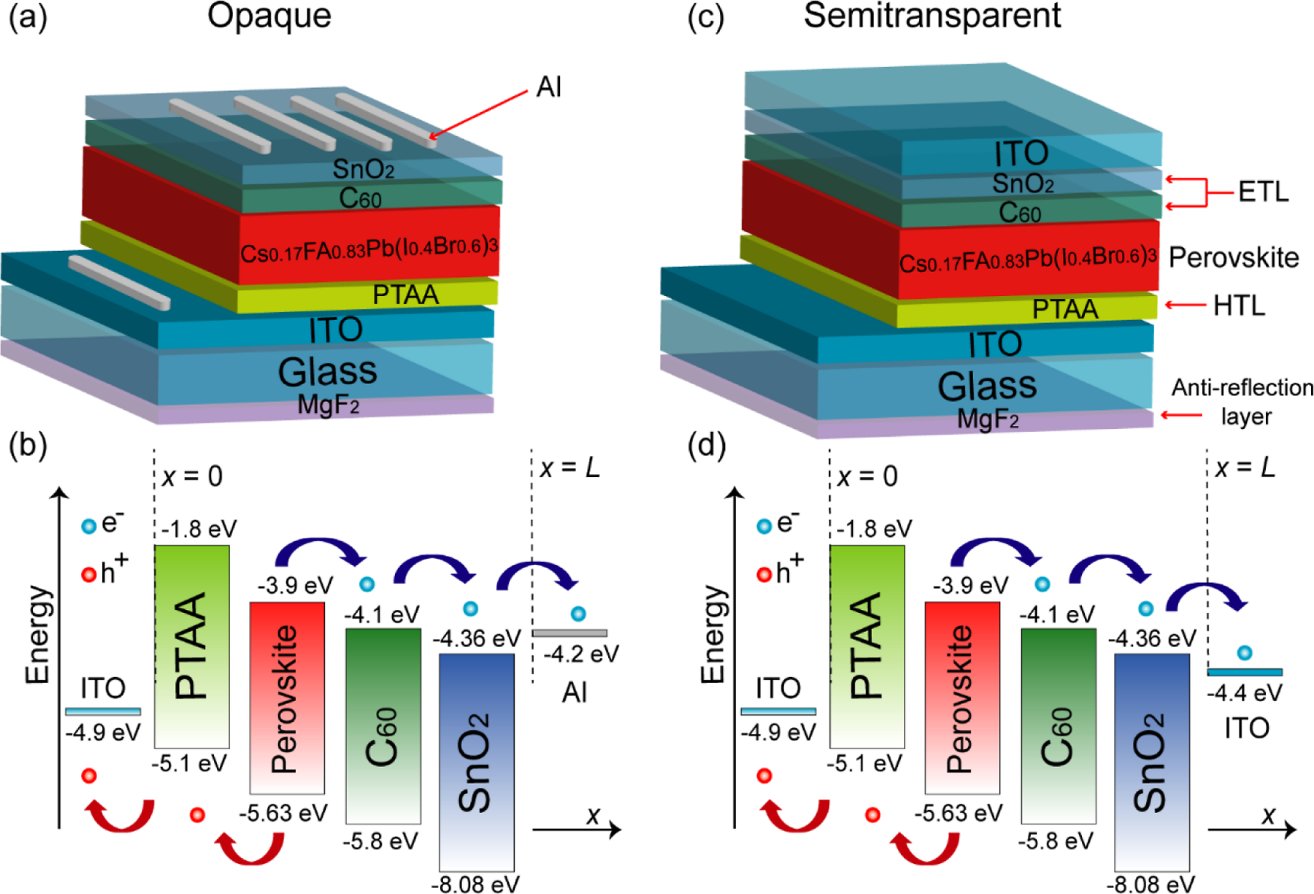
(a,c) Our proposed *p*-*i*-*n* device model for PSCs for efficiency enhancement. (b,d) 1D flat band energy diagram illustrating free electrons and holes generated in the perovskite layer, along with the direction of their respective transport.

Our next step is to solve the Poisson and drift-diffusion equations in a self-consistent manner. This can be achieved by using Gummel iterations combined with Scharfetter–Gummel discretization. The modeling of PSCs is based on our proposed device architecture in which we have used a set of reported material properties of the perovskite layer for simulation purpose,^27^ while the parameters for the HTL, ETL, and contact layers have been selected based on experimental devices developed by other research groups.^36−41^Figure 4a–c represents the structure of *p*-*i*-*n* PSCs in opaque and semitransparent configurations, where *i* is the intrinsic perovskite layer, *n* is the electron transport layer, and *p* is the hole transport layer. Different components of the devices are described as follows: A glass substrate coated with 220 nm thick of a magnesium fluoride (MgF_2_) is used as antireflective coating.^37^ A 110 nm thick indium–tin-oxide (ITO) layer is used as the anode, while the HTL is made of 40 nm thick poly[bis(4-phenyl)(2,4,6-trimethylphenyl)amine] (PTAA).^36,40^ The perovskite layer is based on Cs_*y*_FA_1–*y*_Pb(I_*x*_Br_1–*x*_)_3_ containing a mixture of formamidinium (FA), cesium (Cs), iodine(I), and bromide (Br). The specific composition is Cs_0.17_FA_0.83_Pb(Br_0.4_I_0.6_)_3_ with a band gap of 1.73 eV and a thickness of 320 nm^27^. To facilitate electron transport, an ultrathin fullerene (C_60_) layer and SnO_2_ are employed as a double-layered ETL having a thickness of 20 and 10 nm, respectively.^36^ For the opaque solar cell, a 100 nm thick aluminum (Al) is used as a cathode, while for the semitransparent solar cell, indium–tin-oxide (ITO-r) of 100 nm thickness served as contact to collect the electrons.^39−41^ The electronic energy levels of these devices are illustrated in Figure 4b–d. The correct alignment of energy levels between each constituent layer facilitates the movement of electrons from the perovskite layer to the cathode (either Al or ITO) and the hole transport from the perovskite to the anode (ITO). The initial step is to establish boundary conditions at the contact points and at interfaces between adjacent layers. In this case, the position of different layers within the device is represented by the spatial dimension *x*. On the left side of the device structure, we have the PTAA/ITO interface indicated by *x* = 0. On the right side, we find the SnO_2_/electrode interface (either Al or ITO) specified by *x* = *L*, where *L* represents the thickness of the device.

In our simulation, the solar cell structure is divided into 1D discrete grid with a mesh size of 0.1 nm, which is much smaller than other dimensions considered for the simulation of PSCs.^34,35^ The total simulation volume encompasses 1000 points. A comprehensive description of the iteration process and the discretization method can be found in the literature.^42^ The electrostatic potential is subjected to a boundary condition as outlined in eq 27
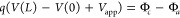
27where *V*_app_ is the applied bias and Φ_*a*_ and Φ_*c*_ are the work functions for anode and cathode, respectively. The relationship between the work functions and the built-in potential is given by eq 28
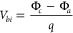
28

Under a steady state, thermodynamic equilibrium is met across the device. According to Boltzmann statistics, the boundary conditions for the charge carriers at the anode (*x* = 0) are given as follows by eqs 29 and 30
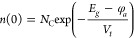
29
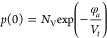
30where *N*_C_ and *N*_V_ are the effective density of states of the conduction and valence band, respectively, *E*_g_ is the band gap of the active layer, and φ_*a*_ is the injection barrier at the anode.

Similarly, the boundary conditions for the cathode at *x* = *L* are expressed by eqs 31 and 32
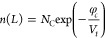
31
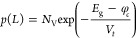
32where φ_*c*_ is the injection barrier at the cathode.

The typical recombination model used to fit transient measurements are based on the simple rate equation , where *n* is the electron density, *G* is the charge carrier generation rate, *k*_1_ is the trap-assisted recombination rate, *k*_2_ is the radiative recombination rate, and *k*_3_ is the Auger recombination rate. Usually, the photogenerated charge carriers in the active layer of a solar cell can recombine via both bimolecular and trap-assisted mechanism. Radiative recombination is modeled by Langevin bimolecular (electron–hole) recombination as given by eq 33
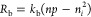
33where *k*_b_ is the bimolecular recombination constant and *n*_*i*_ is the intrinsic carrier concentration. The nonradiative trap-assisted recombination rate (*R*_SRH_) is supposed to occur only within 10 nm thick regions inside the perovskite layer near the HTL and ETL interfaces and is given by the Shockley–Read–Hall (SRH) eq 34
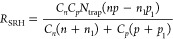
34where *C*_*n*_ and *C*_*p*_ are the capture coefficients for electrons and holes, respectively, *N*_trap_ is the concentration of trapped electrons and holes, and the constants *n*_1_ and *p*_1_ are defined by eqs 35 and 36
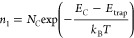
35
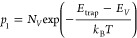
36where *E*_*C*_ and *E*_*V*_ are the conduction and valence band energy, respectively. The trap energies *E*_trap_ are assumed to be located in the middle of the band gap since it leads to the most effective recombination .

## 3 Results and Discussion

### 3.1 Optical and Electrical Simulations under Outdoor Illumination

**Figure 5:**
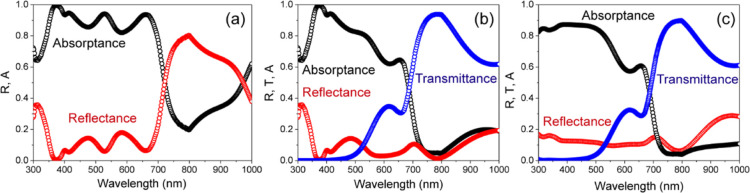
Reflectance, absorptance, and transmittance spectra of Cs_0.17_FA_0.83_Pb(Br_0.4_I_0.6_)_3_-based solar cells for front illumination: (a) opaque and (b) semitransparent devices. (c) Corresponds to optical spectra for rear illumination of the semitransparent solar cell.

We performed an optical simulation of both opaque and semitransparent Cs_0.17_FA_0.83_Pb(Br_0.4_I_0.6_)_3_ solar cells using AM1.5G solar irradiance. The refractive index of different layers needed for the calculations were taken from experimentally determined data^36−41^ and are provided in the Supporting Information. For the calculation, we assumed that all layers had uniform thickness and no surface roughness. We then performed simulations by integrating different layers of PSCs and flat band energy diagrams (Figure 4) into our optical model. Figure 5a–c displays the optical characteristics of the opaque and semitransparent PSCs considering reflectance (*R*), transmittance (*T*), and absorptance (*A*) in each case. The higher absorptance in the opaque solar cell (Figure 5a) compared to the semitransparent device (Figure 5b,c) is attributed to the aluminum rear contact in the opaque PSC, which acts as a mirror, reflecting most photons back into the cell. As a result, the effective absorptance in Cs_0.17_FA_0.83_Pb(Br_0.4_I_0.6_)_3_ has been enhanced. The semitransparent solar cell, under both front and rear illumination, exhibits low light reflection in the visible and infrared wavelengths, primarily due to its high absorptance and transmittance. This high absorptance within the solar cell leads to minimal absorption of low-energy photons, thereby reducing reflection. In contrast, the opaque solar cell exhibits higher reflectance around 730 nm and above. Some of these photons are absorbed through recombination at the back contact, while the rest are reflected back into the atmosphere.

**Figure 6:**
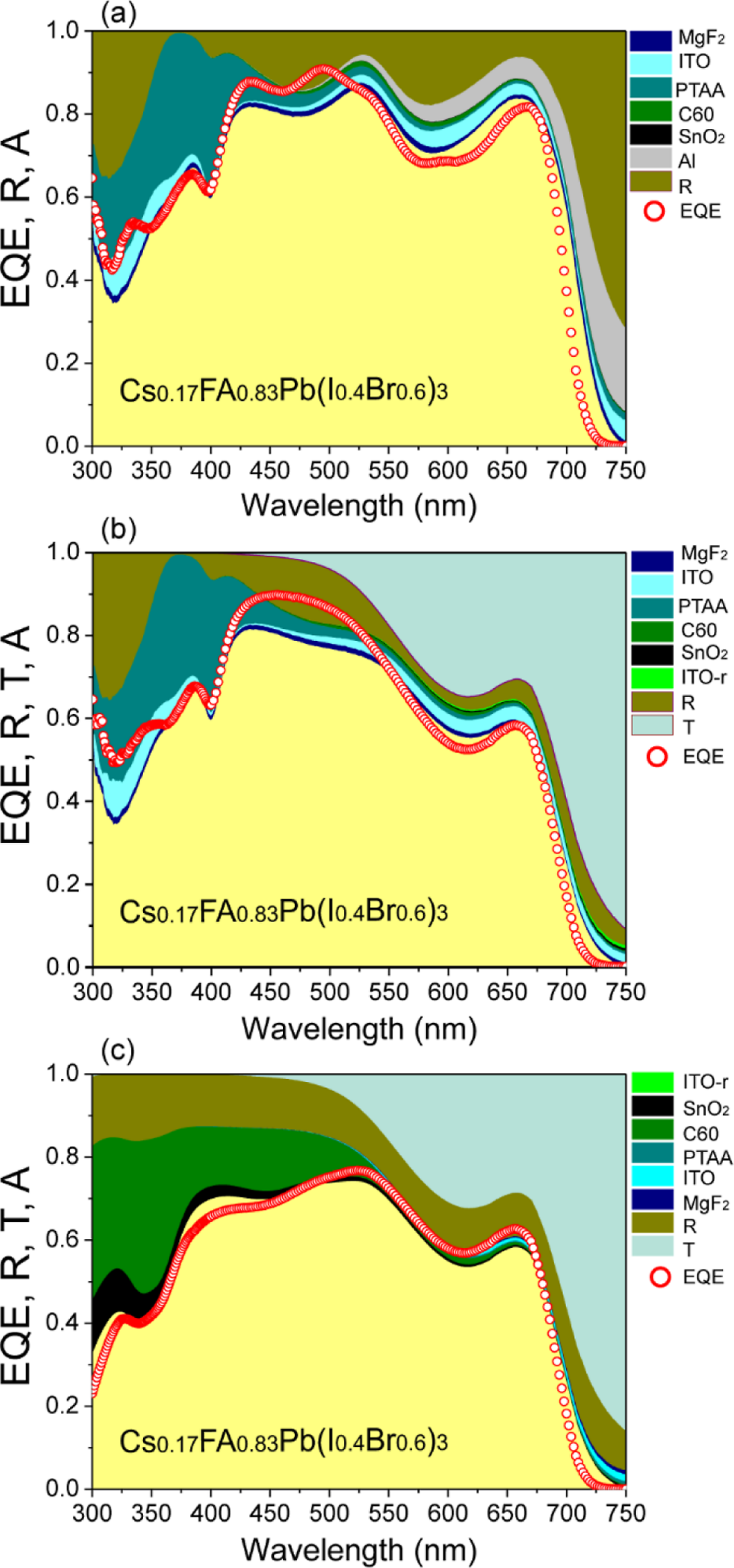
Optical analysis of reflectance, transmittance, and absorptance in each individual layer of (a) opaque, (b) semitransparent solar cells with front and (c) rear illumination. The open circles show the EQE simulation.

There are two main factors for optical loss in PSCs: reflection loss and parasitic absorption loss. These losses can hinder the photon absorption within the device, leading to a significant decay in the effective current density. Consequently, the PSCs suffer a considerable loss in *J*_SC_ and PCE. The spectral performance of PSCs in the opaque and semitransparent model has been illustrated in Figure 6a–c. The reflection loss and parasitic absorption within the PSCs are also shown in these plots. Our analysis focused on the optical loss in the wavelength range of 300–750 nm, corresponding to a band gap of 1.73 eV for Cs_0.17_FA_0.83_Pb(Br_0.4_I_0.6_)_3_ perovskite material. The plots (Figure 6a–c) show that although the perovskite absorbs the majority of incident light, considerable potential still remains for further enhancing its absorption capacity. The opaque solar cell exhibits moderate light absorption by the MgF_2_ layer, along with reflection losses and parasitic absorption by the ITO across the entire wavelength range. However, the inclusion of PTAA as the HTL reduces absorption, particularly in the longer wavelength range (λ ≥ 450 nm), due to its optical properties. On the other hand, both C_60_ and SnO_2_ have a negligible effect on parasitic absorption within the 300–750 nm range. The light absorption by Al is found to increase at longer wavelengths (Figure 6a). The semitransparent solar cell, under front illumination, exhibits significantly reduced reflection and high transmission with negligible light absorption by an ITO-r contact (Figure 6b). Besides, UV light absorption in the PTAA layer causes fewer photons to reach the photoactive perovskite layer in both the semitransparent (front illumination) and opaque cell. Notably, the semitransparent cell under rear illumination exhibits strong light absorption in the short wavelength range (λ ≤ 450 nm) due to the contributions of C_60_ and SnO_2_ layers (Figure 6c).

The external quantum efficiency (EQE) was simulated over the wavelength range of 300–750 nm. We employed the recombination model outlined in the previous report,^44^ assuming that all carriers generated within the carrier collection length (*L*_C_) from the front to end interface contribute to the current generation. In this model, the effects of carrier diffusion and drift within the space charge region are not described separately. Additionally, the effects of band alignment and carrier recombination that occur in bulk, interface, and grain boundary regions were not considered. All complex effects related to the carrier collection are represented by a single analytical parameter of *L*_C_. The relationship between *L*_C_ and the diffusion length (*L*_D_) can be described by *L*_C_ = *W* + *L*_D_, where *W* is the width of the depletion region. Figure 6a–c displays the EQE of both opaque and semitransparent Cs_0.17_FA_0.83_Pb(Br_0.4_I_0.6_)_3_ solar cells, considering a reasonable value of *L*_C_ = 0.5 μm. To determine the *J*_SC_ from the EQE curve, we have multiplied the photon flux with the EQE at the same wavelength using eq 37
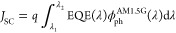
37where ϕ_ph_^AM1.5G^(λ) is the incident photon flux within a certain interval of wavelength from λ_1_ to λ_2_. A simulated *J*_SC_ of 16.76 mA/cm^2^ is determined for the opaque solar cell, while for the semitransparent solar cell, *J*_SC_ values of 14.08 mA/cm^2^ and 13.1 mA/cm^2^ were estimated for front and rear illumination, respectively.

Assuming a quantum efficiency of one, we can identify the optical losses in each layer due to reflection. Actually, the photon loss (*i*.*e*., reflection and parasitic absorption) in a solar cell can be expressed in terms of the photocurrent density. When this photon loss is converted into photocurrent, reflection loss and parasitic absorption in ITO, MgF_2_, PTAA, C_60_, SnO_2_, Al, and ITO-r layers are represented as equivalent current densities. The band gap of the Cs_0.17_FA_0.83_Pb(Br_0.4_I_0.6_)_3_ layer is 1.73 eV; therefore, the maximum short-circuit current density is 24 mA/cm^2^ obtained by integrating the area under the curve of AM1.5G solar spectrum (Figure 1a).

**Figure 7:**
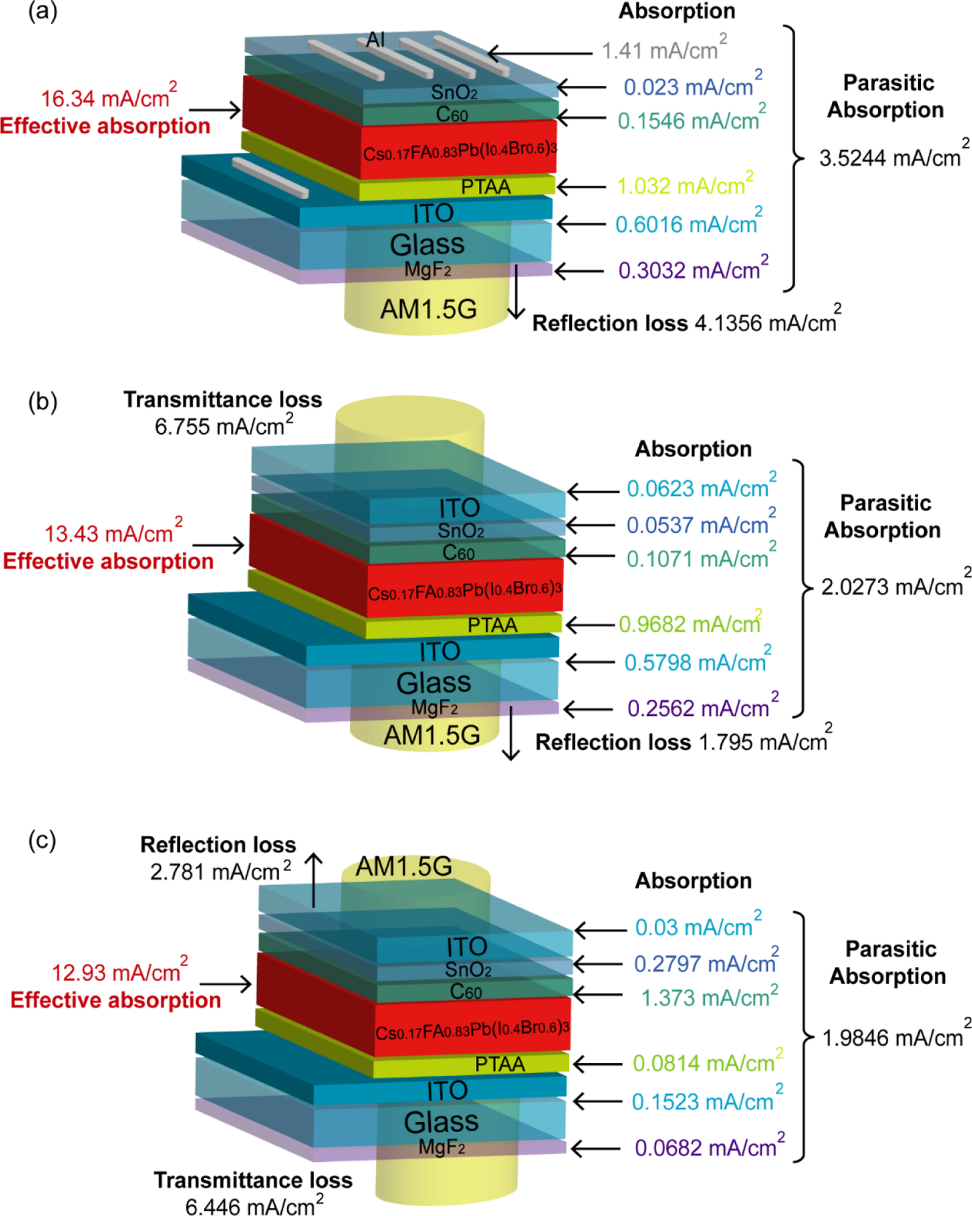
Quantification of the optical losses in the (a) opaque and (b,c) semitransparent devices.

Figure 7a–c quantifies the power losses occurring throughout the entire physical process in each layer of both opaque and semitransparent PSCs. For the opaque solar cell, the effective light absorption in the perovskite layer was increased significantly, achieving a *J*_SC_ of 16.34 mA/cm^2^. Additionally, a reflection loss of 4.13 mA/cm^2^ was found, while the total parasitic absorption within the perovskite layer was 3.52 mA/cm^2^. The simulated values for parasitic absorption in the ITO, MgF_2_, PTAA, C_60_, SnO_2_, and Al layers were determined to be 0.60, 0.30, 1.03, 0.15, 0.023, and 1.41 mA/cm^2^, respectively. It is clear that the main parasitic light absorption occurs in the PTAA and Al layers (Figure 7a). The parasitic losses in the C_60_ and SnO_2_ layers are negligible and can be ignored. In the semitransparent PSCs, the effective light absorption in the perovskite layer is lower compared with the opaque cells. Under front illumination, the semitransparent device shows a remarkable transmittance of 6.75 mA/cm^2^ due to incomplete absorption of photons near the band gap (Figure 7b). We observe an effective light absorption of 13.43 mA/cm^2^ in the perovskite layer, along with a moderate reflection loss of 1.79 mA/cm^2^, which is significantly lower than that in the opaque configuration.

Under rear illumination, the transmittance is nearly identical with that under front illumination, measuring 6.44 mA/cm^2^ (Figure 7c). However, the light absorption in the perovskite layer decreases from 13.43 to 12.93 mA/cm^2^ due to significant absorption by the C_60_ and SnO_2_ layers, which contribute to parasitic losses of 1.373 and 0.28 mA/cm^2^, respectively. In summary, for an opaque solar cell, around 14.7% of the absorbed energy is lost in parasitic absorption and 17.2% in reflection in the case of the opaque solar cell. In semitransparent PSCs, additional losses occur through transmittance. For front-surface illumination, power losses due to parasitic absorption, reflection, and transmission are 8.4%, 7.5%, and 28.1%, respectively (Figure 7b). Under rear illumination, these losses associated with parasitic absorption, reflection, and transmission are 8.3%, 11.6%, and 26.8%, respectively (Figure 7c).

**Figure 8:**
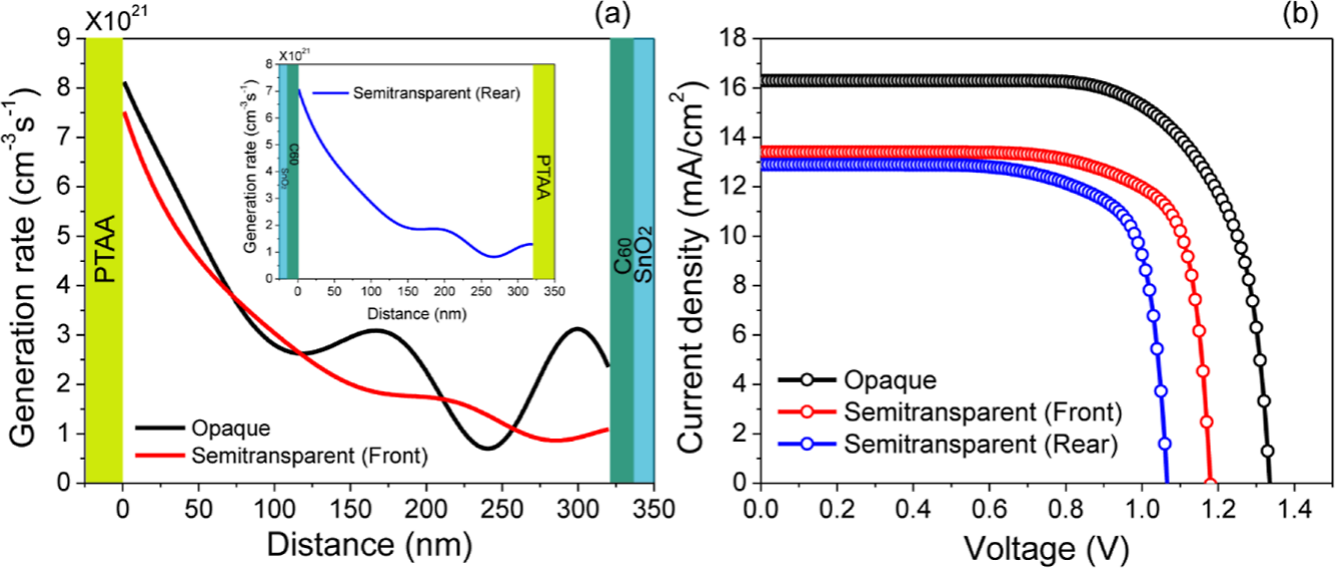
(a) Total charge carrier generation rate profile throughout the perovskite device calculated by TMM. Inset plot shows the generation rate profile for the semitransparent device with rear surface illumination. (b) Simulated *JV* curves for opaque and semitransparent PSCs.

The charge carrier generation profiles for the opaque and semitransparent solar cells under front illumination are presented in Figure 8a. The inset illustrates the photogeneration rate profile of carriers for the semitransparent solar cell under rear illumination. The optical constants of the materials (*n*, κ) introduced into TMM for the calculation of carrier generation profile in PSCs are sourced from the handbook and existing literature (Supporting Information). The current density vs voltage curves (Figure 8b) reveal that the highest carrier generation rate (*G*_max_) leads to a maximum short-circuit current density of 24 mA/cm^2^ in the opaque solar cell. Table 1 shows a summary of the material properties utilized in the simulation.^45−51^ The electron and hole mobilities within the Cs_0.17_FA_0.83_Pb(Br_0.4_I_0.6_)_3_ layer are obtained from the documented literature.^45,46^ Bimolecular recombination occurring inside the perovskite bulk is characterized by a recombination coefficient of 4 × 10^–17^ m^3^/s^46^. Additionally, trap-assisted recombination is observed at the PTTA/Cs_0.17_FA_0.83_Pb(Br_0.4_I_0.6_)_3_ and C_60_/Cs_0.17_FA_0.83_Pb(Br_0.4_I_0.6_)_3_ interfaces. Because the hole capture coefficient is lower than that of electron (*C*_*p*_ < *C*_*n*_*)*, the probability of hole capture per unit time by filled electron trap is lower than that of electron capture by a filled hole trap. This observation supports the persistence of long-lived holes in perovskite materials.^52^

**Table 1:** Basic Material Parameters Used in the Device Simulation for Both Opaque and Semitransparent Solar Cells

parameter	value
CB effective density of states in perovskite layer	2.75 × 10^24^ cm^–3^
VB effective density of states in perovskite layer	3.90 × 10^24^ cm^–3^
perovskite relative permittivity	7^46^
PTAA relative permittivity	3.5^48^
C_60_ relative permittivity	4.25^50^
SnO_2_ relative permittivity	13.5^51^
hole and electron mobility in perovskite	1.8 × 10^–3^ m^2^V^–1^s^–1^^45^
hole and electron mobility in PTAA	4 × 10^–7^ m^2^V^–1^s^–1^^47^
hole mobility in C_60_	1.6 × 10^–5^ m^2^V^–1^s^–1^^47^
hole mobility in SnO_2_	1.4 × 10^–5^ m^2^V^–1^s^–1^
electron mobility in C_60_	1.6 × 10^–4^ m^2^V^–1^s^–1^^49^
electron mobility in SnO_2_	1.1 × 10^–4^ m^2^V^–1^s^–1^^51^
bimolecular recombination constant in perovskite layer	4 × 10^–17^ m^3^s^–1^^46^
electron capture coefficients in perovskite layer	1 × 10^–12^ m^3^s^–1^
hole capture coefficients in perovskite layer	1 × 10^–14^ m^3^s^–1^
HTL/perovskite interface trap density	5 × 10^21^ m^–3^
perovskite/ETL interface trap density	6 × 10^20^ m^–3^
maximum charge generation rate (AM1.5G)	8.12 × 10^27^ m^3^s^–1^ (opaque)
	7.57 × 10^27^ m^3^s^–1^ (semitransparent front illumination)
	7.04 × 10^27^ m^3^s^–1^ (semitransparent rear illumination)

Figure 8b illustrates the *JV* characteristics for both types of solar cells. It is evident that the opaque solar cell exhibits a superior current density and voltage compared to the semitransparent cell under front illumination. The simulated device parameters for the opaque solar cell are as follows: *V*_OC_ = 1.34 V, *J*_SC_ = 16.40 mA/cm^2^, FF = 71.8%, and PCE = 15.8%. Whereas, the semitransparent solar cell with front illumination exhibits a *V*_OC_ of 1.20 V, *J*_SC_ of 13.4 mA/cm^2^, FF of 75.6%, and PCE of 12.07%. As depicted in Figure 8b, the rear illumination on the ITO-r leads to a significant decrease in *V*_OC_ from 1.20 to 1.05 V and a slight reduction in the FF from 75.6–74.1%. Regarding *J*_SC_, a minor decrease from 13.4 to 12.9 mA/cm^2^ was observed because of the increased absorption by the ITO front contact at shorter wavelengths. The poor performance of the semitransparent solar cell with rear illumination is attributed to thickness and optical properties of ITO as front and rear contact.

**Figure 9:**
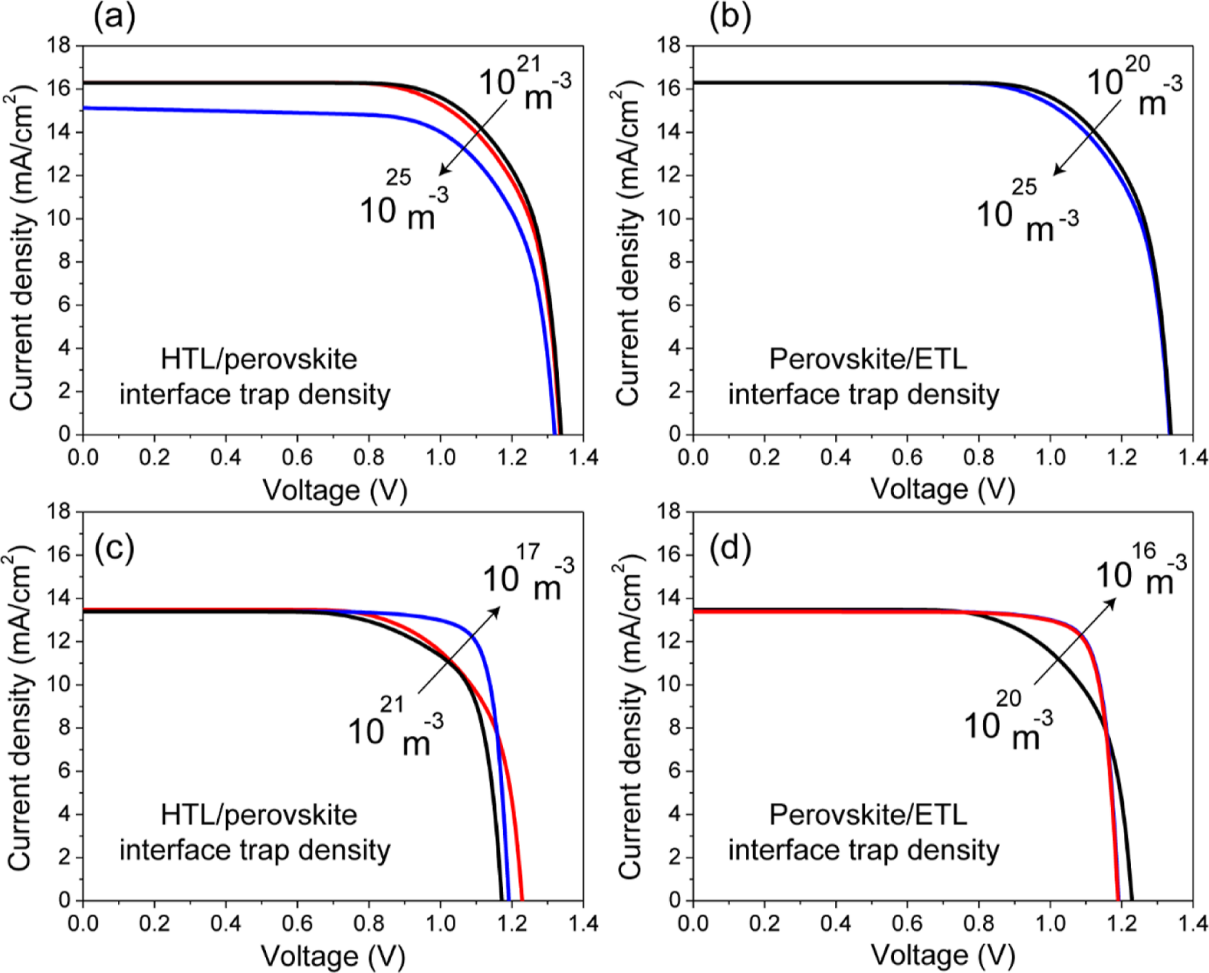
Simulated *JV* curves: (a,b) hole trap density at the HTL/perovskite interface and electron trap density at the perovskite/ETL interface for the opaque solar cell and (c,d) hole trap density at the HTL/perovskite interface and electron trap density at the perovskite/ETL interface for the semitransparent solar cell.

The junction quality is a crucial factor for achieving high-efficiency solar cells. The quantification of interface defect densities leading to recombination loss can shed light on the amount of photocurrent delivered by the system. To study the effect of junction quality on cell performance, we varied the defect densities at the two principle interfaces of HTL/perovskite and perovskite/ETL to determine which junction is more relevant in controlling the efficiency. The *JV* curves in Figure 9a–d illustrate the impact of hole and electron trap densities at the interface between the perovskite and transport layers. It is evident from Figure 9a,b that, for an opaque solar cell, the quality of the HTL/perovskite interface has a major impact on the device performance compared to the perovskite/ETL interface. Figure 9a demonstrates that increasing the defect density at the HTL/perovskite interface from 10^21^ to 10^25^ m^–3^ results in a decrease in solar cell efficiency from 15.8% to a modest efficiency of 14.72%. However, the performance of an opaque solar cell is rather insensitive to the defect densities at the perovskite/ETL interface. The *JV* curves exhibit nearly identical behavior even with an increase in defect densities of up to 5 orders of magnitude across the junction between the perovskite and ETL (Figure 9b). This is because the solar cell is irradiated from the HTL side, leading to a higher concentration of photogenerated electrons and holes near the HTL/perovskite interface in comparison to the back surface, as demonstrated by the generation profile in Figure 8a. The recombination rate is directly proportional to the free charge carriers. Due to an excess concentration of electrons available for recombination with trapped holes on HTL side, this junction has greater impact on solar cell efficiency than perovskite/ETL junction.^53^Figure 9c–d displays the *JV* curves of the semitransparent solar cell, showing a similar behavior to that of opaque solar cells. It is clear that the HTL/perovskite interface significantly affects the device characteristics, resulting in a notable enhancement in PCE of over 15% by reducing defect density from 10^21^ to 10^17^ m^–3^ (Figure 9c). In contrast to the opaque solar cell, the device efficiency in the semitransparent solar cell is greatly influenced by the perovskite/ETL junction (Figure 9d). The efficiency increases from 12.7 to 16.5% by reducing defect densities from 10^20^ to 10^16^ m^–3^ at the interface between the perovskite and ETL. At a defect density below 10^20^ m^–3^, the perovskite/ETL interface exhibits similar behavior in both opaque and semitransparent solar cells.

In PSCs, the quality of the front-side junction (HTL/perovskite) is more critical than that of the back-side interface (perovskite/ETL). Due to illumination at the HTL/perovskite interface, the concentration of free charge carriers is higher, leading to increased SRH recombination losses on the front side.

**Figure 10:**
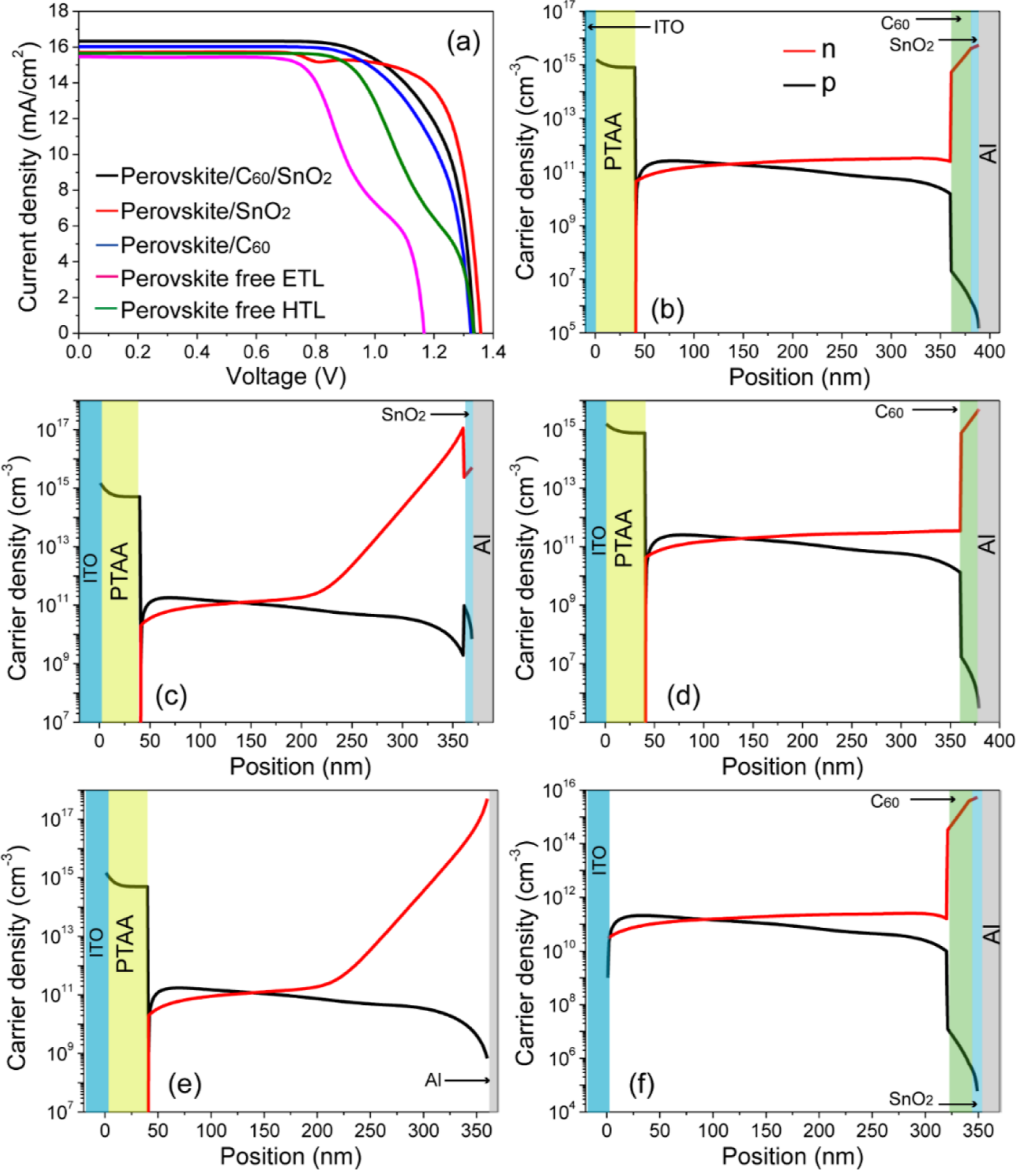
(a) *JV* curves of opaque PSCs with and without transport layers. For PSCs with the ETL, we examined the effects of either C_60_ or SnO_2_ on PV parameters. The carrier density profiles as a function of depth for the simulated opaque solar cell under illumination with a 0 V applied bias are shown in (b) dual layer of C_60_ and SnO_2_ as the ETL, (c) SnO_2_ as a single ETL, (d) C_60_ as the ETL, (e) ETL-free, and (f) HTL-free PSCs. The parameters used are listed in Table 1.

To investigate the impact of a C_60_/SnO_2_ double ETL on device performance, we simulated the opaque solar cell under various scenarios: perovskite/C_60_/SnO_2_ (reference cell), perovskite/SnO_2_, perovskite/C_60_, and ETL-free and HTL-free devices. Figure 10a shows how each modification in the device structure affects the *JV* characteristics. In the reference device (Figure 4a), the ETL is a combination of C_60_ and SnO_2_. When SnO_2_ is used as a single ETL, we observe a small distortion at 0.8 V due to the higher carrier mobility of the perovskite layer than SnO_2_, leading to the accumulation of excess electrons at the interface and thus a high carrier recombination. However, the use of C_60_ as the only ETL did not deteriorate the PV parameters much as compared to the double ETL, yielding a slightly lower *V*_OC_ of 1.31 V, *J*_SC_ of 16 mA/cm^2^, FF of 69.8%, and PCE of 14.63% in contrast to a *V*_OC_ of 1.34 V, *J*_SC_ of 16.40 mA/cm^2^, and PCE of 15.8% in the reference cell.

In the case of ETL and HTL-free PSCs, the physical modeling is based on a metal–insulator–metal mechanism. When the work function of the metal is lower than that of perovskite, electrons transfer from the Fermi level of the metal into the conduction band of the perovskite. The polarized interface creates a Schottky barrier that impedes hole transport from the valence band of perovskite to the metal contact while allowing electrons to flow easily from the perovskite to the metal. This results in a decrease in *V*_OC_, FF, and shunt resistance (*R*_SH_). In contrast, for high work-function metals, an inverse band bending is created at the perovskite/contact interface, which drives the photogenerated holes to transfer easily from the valence band of perovskite to the metal contact, while there is a Schottky barrier toward the electron transfer from the perovskite to metal, producing a higher *V*_OC_. As shown in Figure 10a, the *JV* curves for ETL- and HTL-free devices exhibit an S shape rather than a regular rectangular form. It is seen from the graph that the absence of ETL has a more detrimental effect on the *JV* characteristics compared with the HTL-free configuration. The *V*_OC_, *J*_SC_, and FF in ETL-free devices are very low, achieving a PCE of 8.08% compared to 11.56% efficiency for HTL-free devices. In general, the S-shape in the *JV* curve indicates an accumulation of charge carriers at the interfaces between the perovskite and its adjacent layers (perovskite/C_60_, perovskite/SnO_2_, or perovskite/contacts). To understand the underlying mechanisms related to the space charge effects in the opaque PSCs, we examined the carrier density profiles as a function of the solar cell depth under short-circuit current conditions (Figure 10b–f). The plots clearly show a high hole density at the PTAA/perovskite interface on the order of 10^11^ cm^–3^. Carrier densities are elevated at the contacts (*x* = 0 and *x* = *L*) but drop sharply within the thin transport layers due to their lower conductivity compared to the perovskite, as well as the presence of a strong electric field at perovskite/transport layer interfaces (Figure 10b).

In the device without C_60_, the electron extraction is poor from Cs_0.17_FA_0.83_Pb(Br_0.4_I_0.6_)_3_ perovskite to SnO_2_ due to the low mobility of SnO_2_ as observed in Figure 10c. Additionally, there is an injection barrier of 0.46 eV against electron flow at the perovskite/SnO_2_ interface in the absence of the C_60_ layer (see Figure 4), leading to a considerable charge accumulation at this interface. When C_60_ is used as a single ETL, the charge accumulation is reduced because of its mobility being higher than that of SnO_2_, leading to an increase in FF (Figure 10d).

In the case of the ETL-free PSC (Figure 10e), a high accumulation of electrons was observed at the interface of Cs_0.17_FA_0.83_Pb(Br_0.4_I_0.6_)_3_/Al, forming a Schottky barrier against hole transport. For the HTL-free device (Figure 10f), a high Schottky barrier of 0.73 eV is generated at the ITO/perovskite interface, leading to a significant reduction in electron density, while a notable hole accumulation is observed at the perovskite/ITO interface. This causes a decrease in FF and *R*_SH_, adversely affecting the solar cell performance. Usually, the origin of the S-shape in *JV* curve is attributed to various factors including charge accumulation, injection barrier between the active layer and electrode, low carrier mobility, and the effect of dipole layer.^54^ As shown in Figure 10d,f, the C_60_ devices did not present electron accumulation at the perovskite/C_60_ interface, which is consistent with the experimental data obtained from fluorescence microscopy experiments.^55^

The *JV* curve for semitransparent devices under front surface illumination (Figure S2 of Supporting Information) exhibits a behavior similar to that of opaque solar cell. Consequently, the density profiles for both opaque and semitransparent solar cells at front illumination are almost identical. As shown in Figure S2, the S-shape in the *JV* curve for the ETL-free semitransparent solar cell is less pronounced than that observed in the opaque solar cell (Figure S2 of Supporting Information), which is related to the higher work function of ITO-r compared to Al.

Another way to prove the efficient role of a double layer of C_60_/SnO_2_ is by examining the plots of potential as a function of PSC depth for the opaque solar cell (Figure S3 of Supporting Information). The inclusion of C_60_ improves the equilibrium in carrier density at the interfaces of perovskite/transport layers, thereby improving its conductivity. The high carrier mobility in C_60_ reduces the potential drop across the perovskite layer, leading to a strong electric field at the interface between the bulk perovskite and C_60_. This improves the charge extraction and reduces the recombination loss, leading to an improvement in FF.

Next, we examined the impact of carrier mobility in the C_60_ ETL on the device efficiency of the opaque solar cell, while keeping all other parameters constant (Table 1). Reducing the electron mobility in the C_60_ layer hinders the swift escape of electrons from the interface. As a result, they accumulate at the perovskite/C_60_ interface, where they recombine with holes in the perovskite, leading to pronounced carrier recombination. The *JV* shows a pronounced S-shape attributed to poor charge extraction (Figure S4 of Supporting Information).

**Figure 11:**
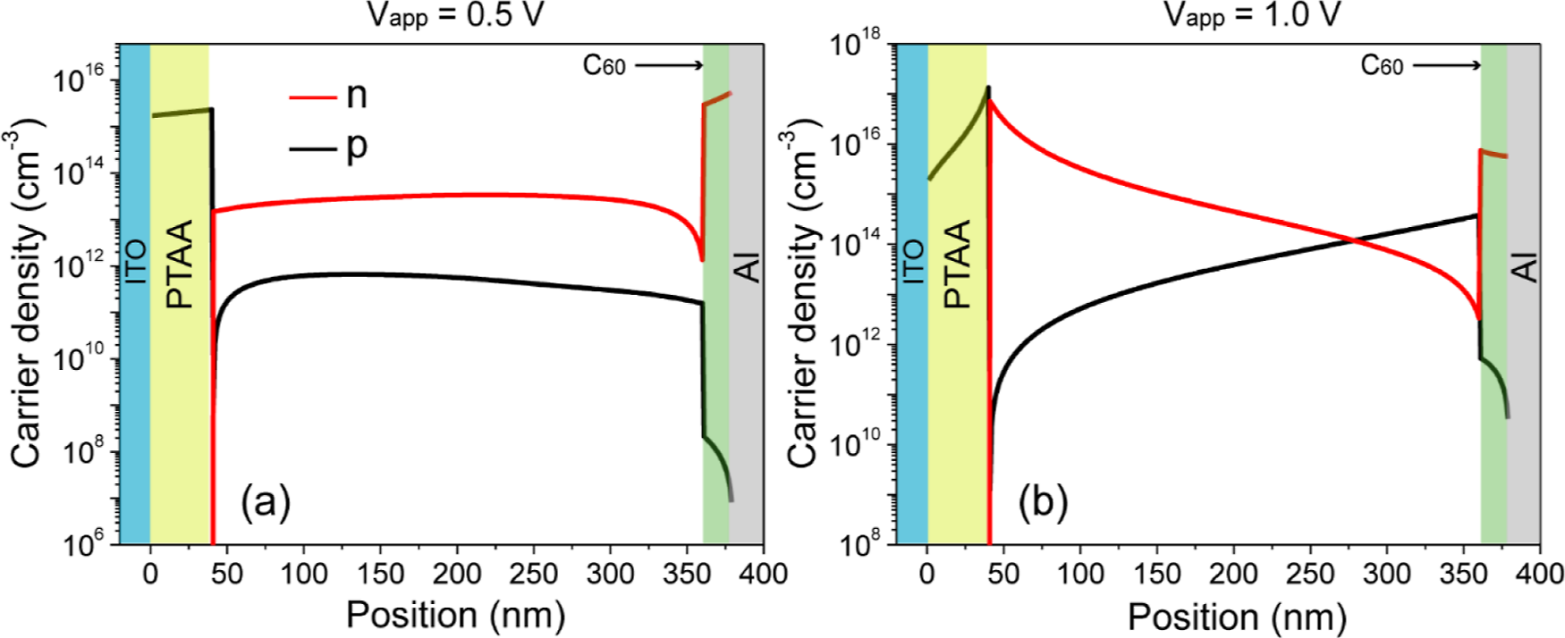
Charge carrier density profiles for the opaque solar cell considering the lowest electron mobility in C_60_ under (a) 0.5 V and (b) 1.0 V applied bias.

Figure 11a,b shows the carrier density profiles for the opaque device considering the minimum carrier mobility of 1 × 10^–5^ m^2^/(V s) in the single C_60_ ETL under applied biases of 0.5 and 1 V. At 0.5 V, the electrons fail to reach the perovskite layer, as evidenced by the sharp contrast in electron density on either side of the Cs_0.17_FA_0.83_Pb(Br_0.4_I_0.6_)_3_/C_60_ interface. As the applied voltage increases beyond 0.5 V, holes accumulate more significantly on the opposite side of the interface, forming a dipole. This dipole effectively reduces the electron injection barrier as long as the injection barrier persists. Consequently, the net current in the device remains near zero until the applied voltage is sufficient to overcome the injection barrier, which occurs at approximately 1 V. It is important to note that the width of the flat region in the *JV* curve contracts as the interface mobility decreases (Figure S4). This is because lower interface mobility requires a stronger net electric field and therefore a higher applied voltage to establish an injection current across the interface.

**Figure 12:**
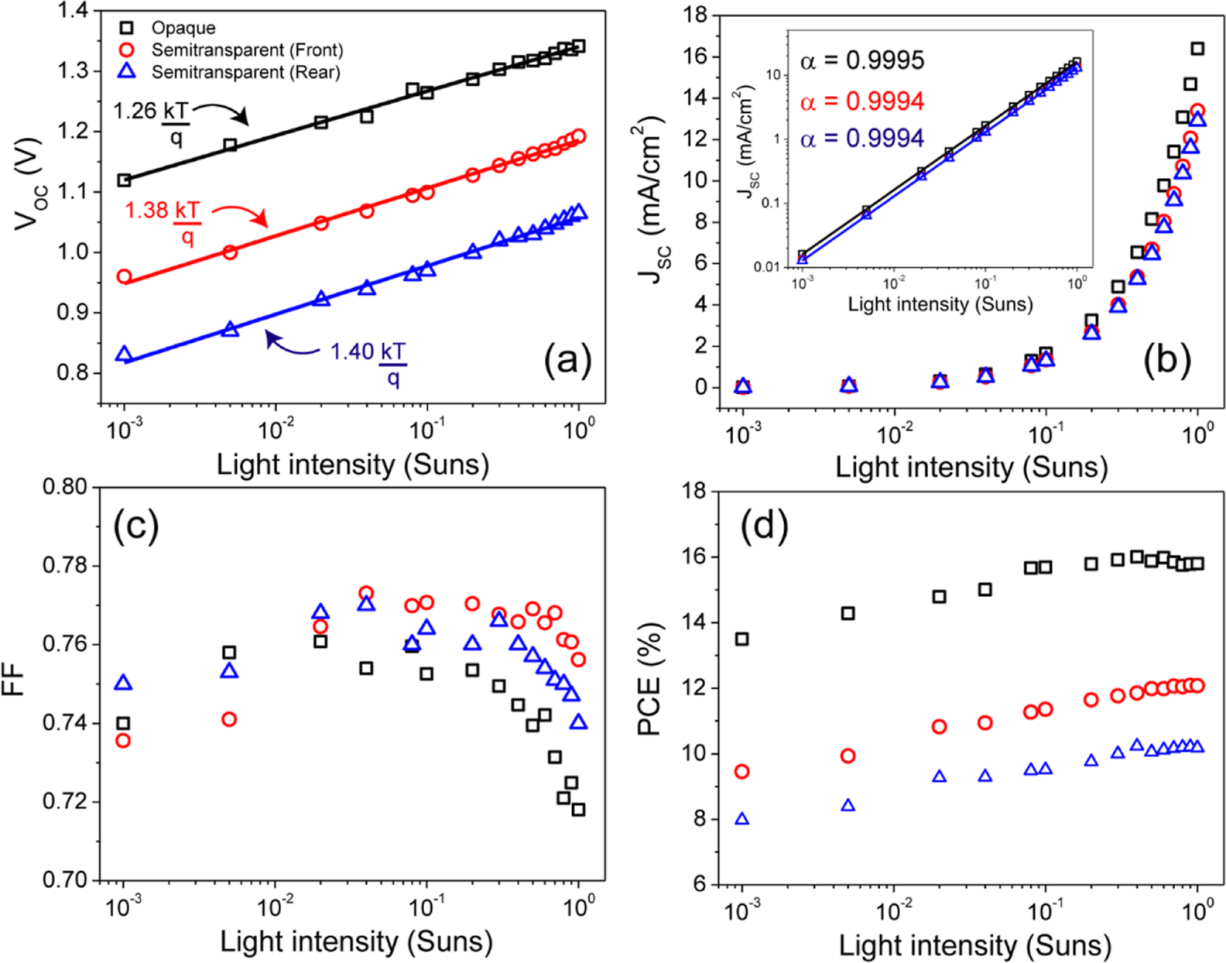
Light intensity-dependent solar cell parameters: (a) *V*_OC_, (b) *J*_SC_, (c) FF, and (d) PCE.

The model was further verified by determining the solar cell parameters under different light intensities, as *V*_OC_ depends on the incident photon flux. The simulated *JV* characteristics of the devices over an intensity range of 1 Sun to 10^–3^ Sun are shown in Figure 12a–d. The dependence of *V*_OC_ on light intensity is crucial in analyzing trap-assisted recombination, which enables to calculate the ideality factor using eq 38
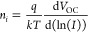
38where *n*_*i*_ is the ideality factor, *k* is the Boltzmann constant, *T* is the temperature, *q* is the elementary charge, and *I* is the normalized light intensity.

The ideality factor of a diode provides insights into the dominant charge recombination mechanism in a solar cell, distinguishing between SRH recombination mediated by a trap state (*n*_*i*_ > 1) or bimolecular recombination (*n*_*i*_ = 1).^56^ This simplified explanation is widely accepted within the PV research community. The value of *n*_*i*_ can be determined from the slope of *V*_OC_ vs light intensity plot. Figure 12a shows the dependence of *V*_OC_ on light intensity, giving a slope of  for the opaque device. On the other hand, in the semitransparent device, we obtained the slopes of  and  under front and rear illumination, respectively. When the ideality factor approaches 1, trap-assisted recombination in a solar cell is minimized. This indicates that the opaque solar cell is more efficient in suppressing trap-assisted recombination. We further investigated the relationship between light intensity and *J*_SC_ to understand charge recombination with α ≤ 1. As shown in Figure 12b, the values for all devices are close to unity, indicating a linear correlation between the photocurrent and light intensity. This behavior suggests minimal bimolecular recombination during charge extraction under short-circuit conditions.^57^

Among the solar cell parameters, the dependence of FF on light intensity is highly sensitive to the changes in ohmic loss and interface defects.^58^ Even a small change in light intensity can significantly affect the final curve shape under different illumination intensities, as shown in Figure 12c. The overall shape of the FF curve suggests a competition between two predominant recombinations (bimolecular and trap-assisted recombination) in both opaque and semitransparent solar cells. As depicted in Figure 12c, FF decreases rapidly with increasing light intensity above 0.1 Sun, which can be attributed to the dominance of bimolecular recombination. The recombination rate is proportional to the carrier densities and it decreases by reducing the light intensity. However, below 0.1 Sun, the FF decreases monotonically with decreasing light intensity, indicating pure trap-assisted recombination. For trap-mediated charge recombination, the rate at which free charges recombine with trapped charges decreases as light intensity increases. The opaque device exhibits a maximum value of FF of 76.1% at a light intensity of 0.02 Sun. This value is still much lower than the theoretical SQ limit of FF at this band gap. To illustrate, while the SQ limit for a reference device with a 1.73 eV band gap is 91%, the simulated opaque solar cell achieves a FF of 71.8% at 1 Sun, marking a difference of 19.2%.

On the other hand, for semitransparent solar cells with front-side illumination, there is a significant increase in the FF, reaching 77.3% at 0.04 Sun, which is even higher than that of the opaque solar cell. When comparing the SQ limit with that of an ideal device, we observed a significant decrease in the band gap of approximately 15%. A similar trend is observed under rear illumination, where the FF differs slightly from front-side illumination by about 6%. The improved performance of the semitransparent solar cell can be attributed to the interface between the ETL and the contact. The primary function of the ETL is to collect the photogenerated electrons from the perovskite and meanwhile to block holes to avoid recombination. Figure 4d illustrates how the ETL effectively prevents the reverse flow of electrons from the C_60_/SnO_2_ layer back into the perovskite layer. A key aspect to consider is not only the conduction band offset (CBO) between the ETL and the perovskite layer but also the conduction band alignment between the ETL and the ITO contact (Figure 4d). In the opaque device, we obtain an electron affinity difference of +0.16 eV between the SnO_2_ (φ = 4.36 eV) and Al contact (φ = 4.2 eV). In contrast, this difference in electron affinity between the SnO_2_ ETL and ITO-r rear contact (φ = 4.4 eV) is −0.04 eV. The enhanced FF can be interpreted as the result of more efficient electron extraction at the C_60_/SnO_2_/ITO interface.

Figure 12d shows the dependence of PCE on light intensity. Initially, it shows a slow increase in the low-intensity range, followed by a saturation point around 0.1 Sun and above. This can be understood in terms of the *V*_OC_ and *J*_SC_ of the device. Because of the logarithmic relationship between *V*_OC_ and *J*_SC_, a decrease in *J*_SC_, caused by the reduced light intensity results in an equivalent drop in *V*_OC_ in absolute terms. Consequently, a solar cell with a higher *V*_OC_ would experience a smaller percentage decrease in efficiency under indoor lighting conditions.

A simulated PCE of 16% was obtained for an opaque solar cell at 0.4 Sun. However, for bifacial solar cells, the efficiency is consistently higher under front-side illumination compared to rear-side illumination. For semitransparent solar cells, the PCE is 12% at 0.5 Sun and 10.2% at 0.4 Sun for front and rear illumination, respectively. Generally, the opaque solar cells demonstrate greater efficiency than their semitransparent counterparts because they utilize light more effectively. This advantage is largely due to their opaque rear electrodes, which enhance light trapping and absorption of incoming radiation (Figure 7).

### 3.2 Optical and Electrical Simulations under Indoor Illumination

Based on the analysis provided in Section 2, it can be concluded that the commonly utilized perovskite materials, such as methylammonium lead halides exhibit an optical band gap of around 1.57 eV, which is considered to be too narrow for effective energy harvesting from indoor lighting sources.^20,21^ According to theoretical modeling, the absorber materials with a band gap higher than 1.6 eV are suitable for IPV applications because the emission spectra of indoor illumination lie in the range of 200–700 nm.^20^ In this case, our new perovskite model based on an organic/inorganic Cs_0.17_FA_0.83_Pb(Br_0.4_I_0.6_)_3_ absorber material with a band gap of 1.73 eV is expected to improve the efficiency under indoor lighting conditions. In fact, perovskites with a high band gap energy (>1.5 eV) leads to a higher SQ limit under artificial light sources until the band gap energy reaches ∼2 eV (Figure 2). As shown in Figure 12c, the organic/inorganic Cs_0.17_FA_0.83_Pb(Br_0.4_I_0.6_)_3_ demonstrates a significantly higher FF under low light intensity compared with high light intensity.

**Figure 13:**
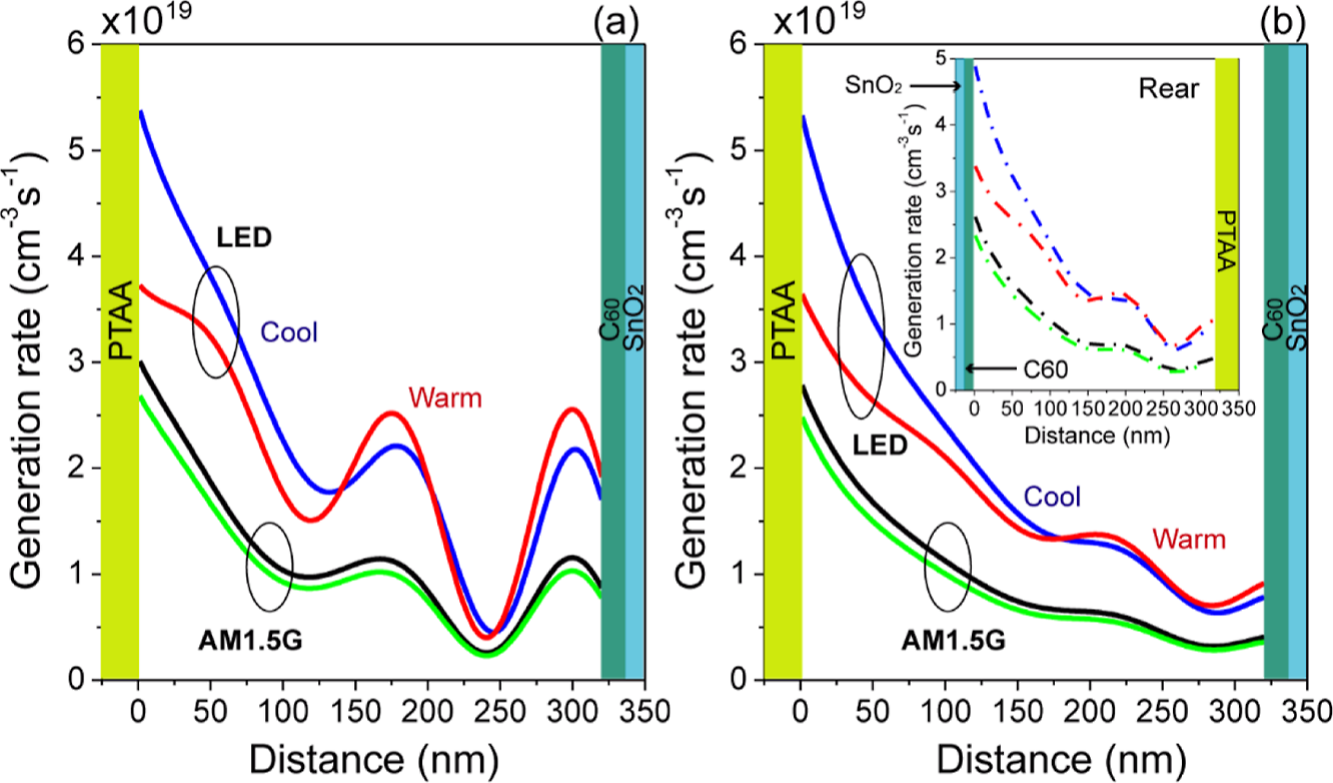
Dependence of the carrier generation rate vs the sample depth considering an irradiance of 3.3 and 3.7 W/m^2^ for a Cs_0.17_FA_0.83_Pb(Br_0.4_I_0.6_)_3_-based PSC: (a) opaque and (b) semitransparent devices. The inset figure shows the rear illumination.

Understanding the characteristics of illumination spectra and their impact on device performance is crucial for determining how warm and cool LED illumination influences the generation of free carriers within the active layer. Here, we follow the same model described in Section 2.2 to determine the free carrier generation rate as a function of the wavelength. LED irradiance spectra for warm and cool color temperatures were determined previously in Section 2.1, producing 3.3 and 3.7 W/m^2^ energy flux, respectively. These spectra were used to calculate the generation rate as a function of thickness, and the results are shown in Figure 13a–b. The AM1.5G spectrum is compared with indoor illumination from cool and warm LEDs at the same power density as displayed in Figure 13a,b. Using artificial light sources, we observed a significant increase in the carrier generation rate compared to the AM1.5G spectrum for both opaque and semitransparent devices with front and rear illumination (Figure 13b and the inset). The depth-dependent carrier generation rate is intense at the surface of the active layer and did not exhibit an important decay with the increase in thickness under artificial light sources. On the other hand, for the AM1.5G spectrum at the same power density, the generation rate is almost constant throughout the entire thickness of the active layer.

The photocurrent density (*J*_ph_) can be expressed using eq 39
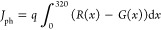
39where *R*(*x*) is the carrier recombination rate.

**Figure 14:**
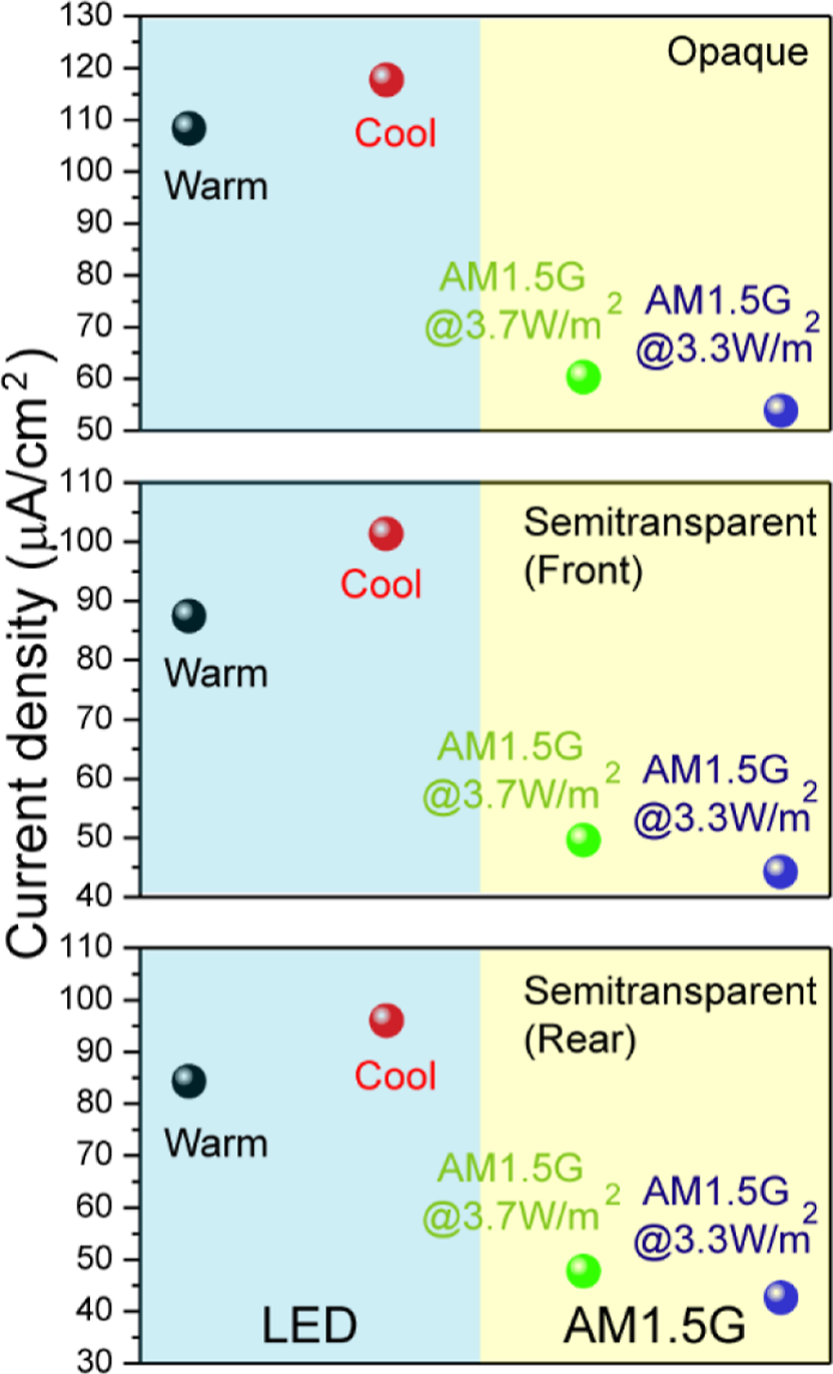
*J*_ph_ calculated for both opaque and semitransparent devices, considering the LED spectrum and AM1.5G irradiance.

Figure 14 illustrates the current density obtained from the solar cell using the AM1.5G spectrum and LED bulbs for opaque and semitransparent devices in the absence of recombination. For the opaque solar cell, the photocurrent density under cool LED was 117.7 μA/cm^2^, which was reduced to 108.3 μA/cm^2^ when using the warm LED. However, these *J*_ph_ values were nearly 2 orders of magnitude higher than those obtained under AM1.5G irradiance at the same intensities of 3.3 W/m^2^ and 3.7 W/m^2^. A similar behavior was observed for the semitransparent solar cell when illuminated from the front. The *J*_ph_ values under cool and warm LED illumination were 101.3 and 87.4 μA/cm^2^, respectively. In contrast, when illuminated from the back, the photocurrent density under cool LED was 96 μA/cm^2^, that is very similar to 84.3 μA/cm^2^ observed under warm LED.

The superior carrier generation rate observed with artificial light sources, in contrast to the AM1.5G spectrum, is attributed to the broader spectrum of AM1.5G compared to LEDs, which are not fully utilized, leaving a significant portion of it remaining unabsorbed and yielding lower *J*_ph_ values.

**Figure 15:**
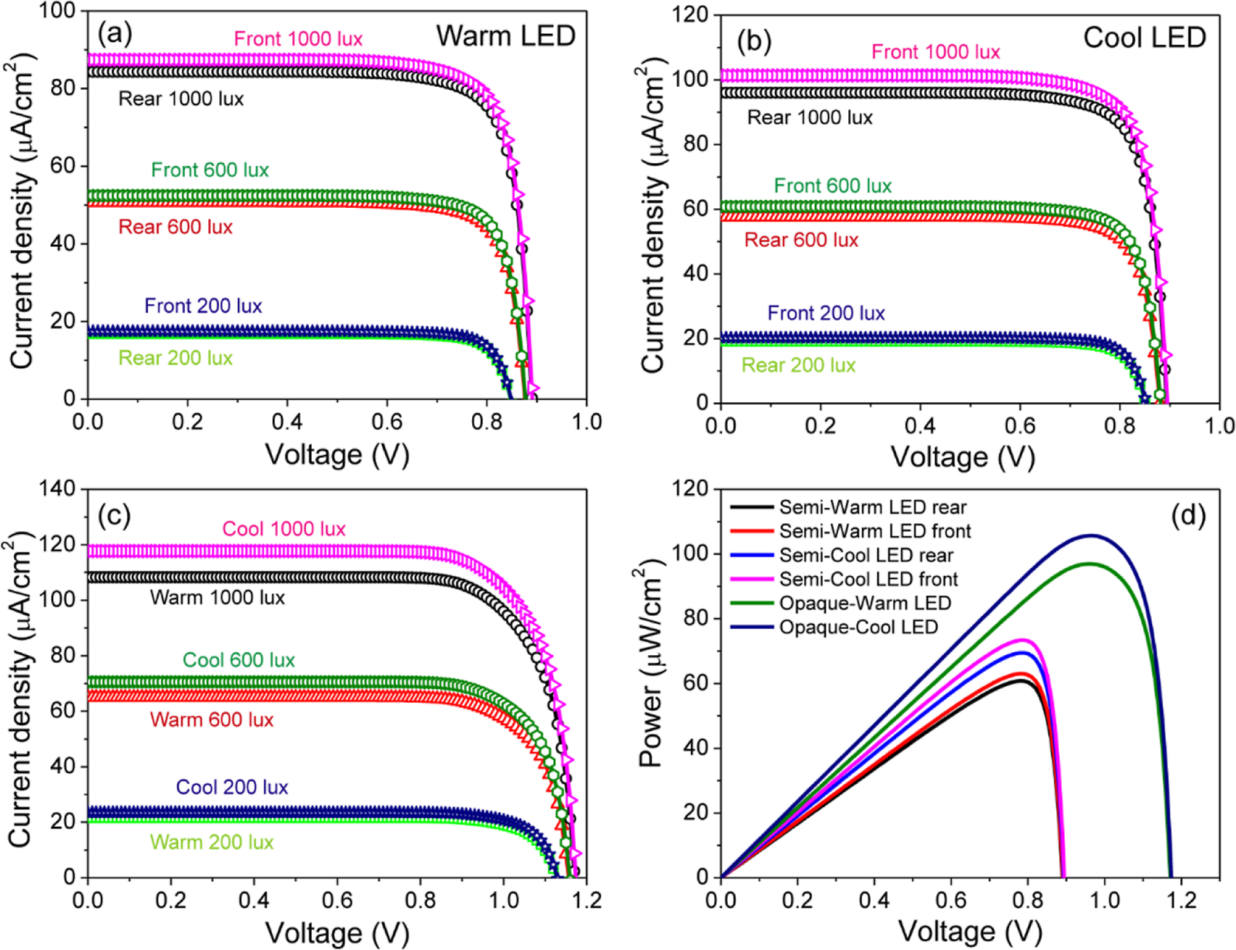
*JV* curves of Cs_0.17_FA_0.83_Pb(Br_0.4_I_0.6_)_3_ devices measured under different illumination sources: (a) semitransparent device with warm LED, (b) semitransparent device with cool LED, (c) opaque device, and (d) power output at 1000 lx for the opaque and semitransparent devices with rear and front illumination.

Figure 15a–c presents the *JV* curves of the semitransparent and opaque devices under warm and cool LED sources. These curves reveal that the Cs_0.17_FA_0.83_Pb(Br_0.4_I_0.6_)_3_ opaque device not only exhibits a higher *V*_OC_ but also generates a higher photocurrent compared with the semitransparent solar cells under LED illumination. For instance, at 1000 lx, the semitransparent device with warm LED (Figure 15a) exhibited a *V*_OC_ of 0.88 V and a *J*_SC_ of 87.32 and 84.16 μA/cm^2^ by illuminating the front and back surface, respectively. In contrast, under AM1.5G (Figure 14) at the same intensity of 1000 lx, the device showed a *V*_OC_ of 0.85 V and a *J*_SC_ of 44.4 μA/cm^2^ for front illumination and 43.5 μA/cm^2^ for back illumination.

The FF was improved to 0.80 and 0.79 under front and rear illumination, resulting in PCEs values of 18.56% and 17.72%, respectively. In comparison, under the AM1.5G spectrum, FF was 0.74 and 0.76, yielding a PCE of approximately 15.43% for both front and rear illumination. Under cool LED illumination (Figure 15b), *V*_OC_ was 0.89 V for both front and rear illumination, while *J*_SC_ increased to 101.3 μA/cm^2^ for front illumination and 96.27 μA/cm^2^ for rear illumination. The FF remained unchanged, resulting in PCE values of 19.49% and 18.29% for front and rear illumination, respectively. For the opaque device, a higher *V*_OC_ of 1.17 V was achieved under both warm and cool LED illumination (Figure 15c), which is attributed to its favorable optical properties. Additionally, the *J*_SC_ increased to 118.13 and 108.57 μA/cm^2^ under cool and warm LED sources, respectively. This led to a substantial improvement in PCE, reaching 28.38% and 25.40% under cool and warm LED illumination, respectively.

The power output for the opaque device increased to 106.25 μW/cm^2^ under cool LED illumination compared to the semitransparent device, as shown in Figure 15d. Cool LED sources consistently delivered higher power values in comparison to warm LEDs. For the semitransparent device, the power output under warm LED illumination was 60.71 μW/cm^2^ for rear illumination and 63.16 μW/cm^2^ for front illumination. In contrast, when illuminated with a cool LED source, the power slightly increased to 69.53 μW/cm^2^ for rear illumination and to 73.66 μW/cm^2^ for front illumination.

The findings confirm that increasing the band gap of the active layer improves device efficiency, as predicted by the SQ limit. Similar to their performance under 1 Sun illumination, these perovskite devices show enhanced efficiency when exposed to artificial LED light sources. Both cool and warm LED lights resulted in comparable performance, likely due to the effective absorption of visible light by the perovskite layer within a narrow wavelength range. The key distinction between sunlight and indoor lighting lies in their spectral distribution: sunlight spans a broad spectrum, from ultraviolet to infrared, whereas indoor lighting primarily emits within the visible spectrum.

Another key difference is light intensity; indoor light sources can be over 500 times less intense than sunlight. The results of this study highlight the significant potential of the Cs_0.17_FA_0.83_Pb(Br_0.4_I_0.6_)_3_ perovskite material for indoor PV applications. Additionally, the use of indoor perovskite devices may mitigate photooxidation, which deteriorates their performance and reduce the lifespan when exposed to outdoor conditions.

## 4 Conclusions

In summary, we proposed a novel design for an inverted PSC and conducted performance analysis of Cs_0.17_FA_0.83_Pb(Br_0.4_I_0.6_)_3_ perovskite material in opaque as well as semitransparent configurations under artificial light sources with the aim to evaluate their potential as an indoor PV system. By considering radiative recombination as the only loss mechanism, we determined the SQ limits using two representative indoor light sources: warm and cool LEDs. The SQ limit identified optimal band gaps for indoor lighting at 1000 lx, which were 1.7 eV for warm LED and 1.8 eV for cool LED. The TMM proved highly effective for analyzing optical and parasitic losses in individual layers of both opaque and semitransparent PSCs, providing accurate insights into the limitations of different device architectures. For the opaque device, the main parasitic light absorption occurred in the PTAA and Al layers, while the parasitic absorption in C_60_ and SnO_2_ ETLs was minimal. Regarding junction quality and defect density, the HTL/perovskite interface had a significant impact on the PCE of opaque devices, whereas in semitransparent devices, the ETL/perovskite interface played a more critical role in determining the performance. Simulated current–voltage curves at different light intensities offered insights into recombination mechanisms and carrier generation profiles in the active layer. The dependence of *V*_OC_ on light intensity suggests that the trap-assisted recombination is suppressed in the opaque device, while bimolecular recombination is reduced in semitransparent solar cells. Overall, opaque devices performed better than their semitransparent counterparts under indoor lighting, achieving a higher PCE of 15.8%, compared to 12.07% and 10.14% for front and rear illumination in semitransparent devices. Notably, the best-performing opaque cell achieved a PCE of 28.38% under cool LED illumination at 1000 lx, a performance comparable to GaAs devices. These findings highlight the significant potential of the Cs_0.17_FA_0.83_Pb(Br_0.4_I_0.6_)_3_ perovskite material for indoor PV systems, providing a comprehensive understanding of performance factors related to light intensity and paving the way for further advancements in highly efficient indoor PV technology.

## Supporting Information Available

Plots for optical constants vs wavelength, current density–voltage curves, carrier density and potential profiles as a function of depth, current density–voltage curves for different carrier mobilities, and table containing refractive index and extinction coefficients of the constituent layers in the device (PDF)
