# Supplementary material for: Insights from Theoretical Modeling of Cesium-Formamidinium-Based Mixed-Halide Perovskite Solar Cells for Outdoor and Indoor Applications
Source: ACS Omega. 2024 Nov 4;9(46):46157–75. doi: 10.1021/acsomega.4c06752 (PMC11579932; doi:10.1021/acsomega.4c06752)
Supplement: Supplementary file 1 — ao4c06752_si_001.pdf [file ao4c06752_si_001.pdf]

Supporting Information

Insights from theoretical modelling of Cesium-Formamidinium-Based Mixed-Halide Perovskite Solar Cells for outdoor and indoor applications

David Mora-Herrera[1*], Jorge Alberto Polito-Lucas[2], Mou Pal[2*]

[1]Ingeniería en Energía. Universidad Politécnica de Amozoc. Av. Ampliación, Luis Oropeza No. 5202, C.P. 72980 Amozoc, Pue.

[2]Instituto de Física, BUAP, Av. San Claudio y Blvd. 18 Sur Col. San Manuel, Ciudad Universitaria, C.P. 72570 Puebla, México.

[*]Corresponding author: D. Mora-Herrera, e-mail: david.mora@upamozoc.edu.mx

Mou Pal, e-mail: mou@ifuap.buap.mx

Figure S1 displays the variation of optical constants (refractive index *n* and extinction coefficient κ) as a function of wavelength for each layer used in the devices. The optical constants for each layer of the PSC are detailed in **Table S1**.

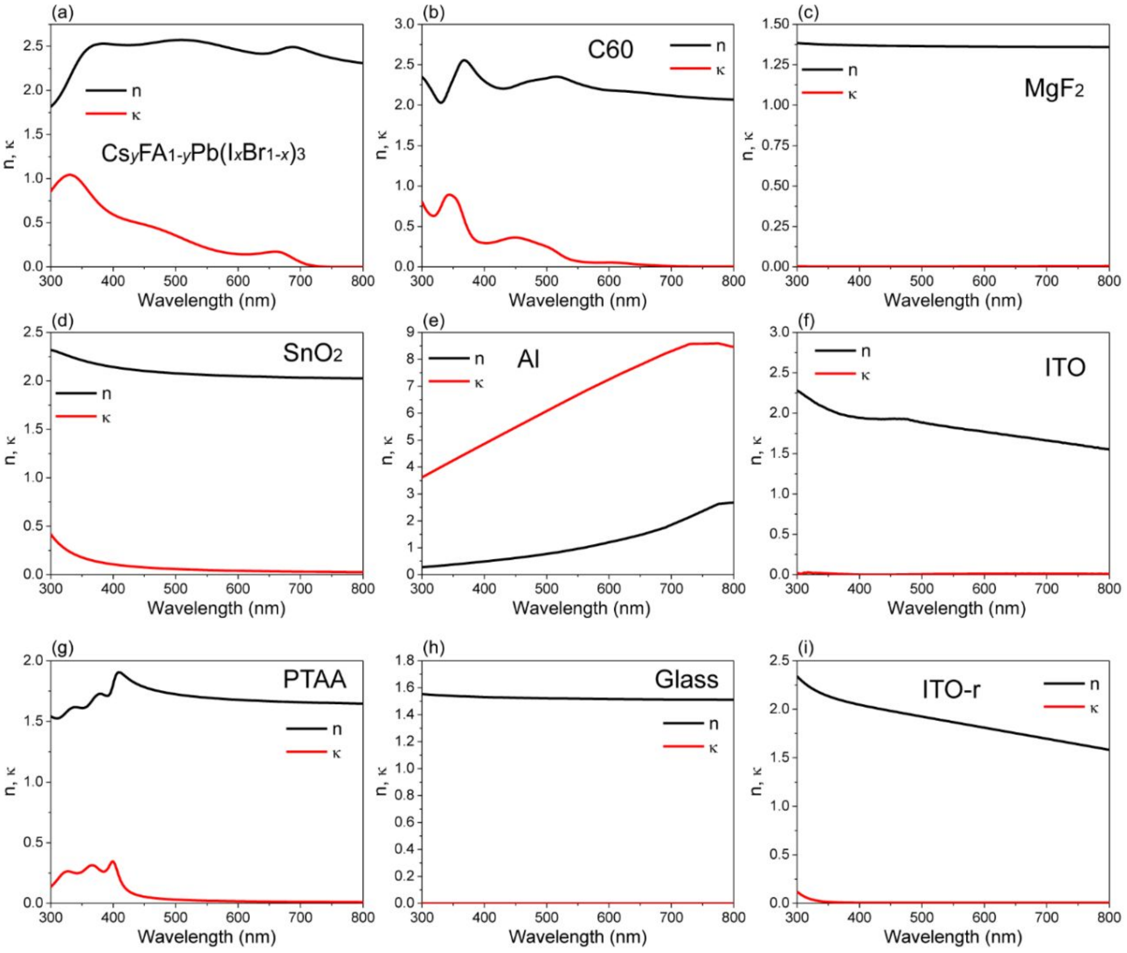

Figure S1. Optical constants of (a) perovskite layer, (b) C_{60} electron transport layer, (c) MgF_2 antireflection layer, (d) SnO_2 electron transport layer, (e) Al contact, (f) ITO contact, (g) PTAA hole transport layer, (h) glass and (i) ITO-r contact.

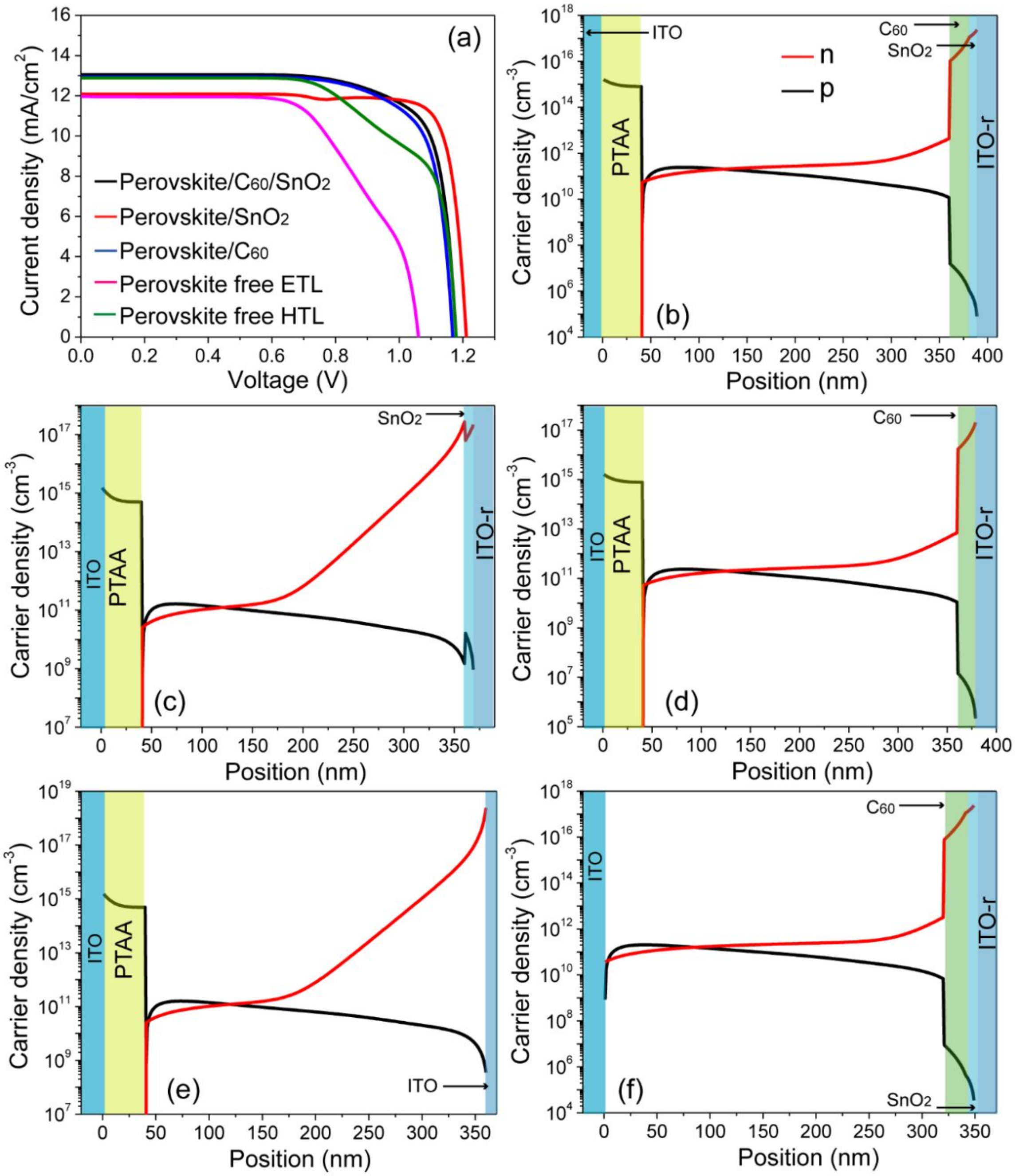

Figure S2. (a) The *JV* curves of semitransparent *PSCs* (front illumination) with or without transport layers. For *PSCs* with ETL, we analyzed the impact of using either C_{60} or SnO_2 on the *PV* parameters. Furthermore, the carrier density profiles as a function of depth for the simulated semitransparent solar cell under illumination at $V = 0$ V bias are shown for: (b) a dual layer of C_{60} and SnO_2 as ETL, (c) SnO_2 as ETL, (c) C_{60} as ETL, (e) ETL-free and (f) HTL free *PSCs*. The parameters used in the simulation are listed in **Table 1**.

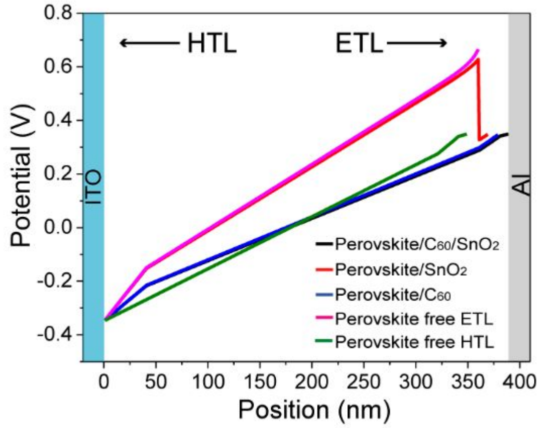

Figure S3. Comparison of potential profiles in opaque solar cell with different interface configurations.

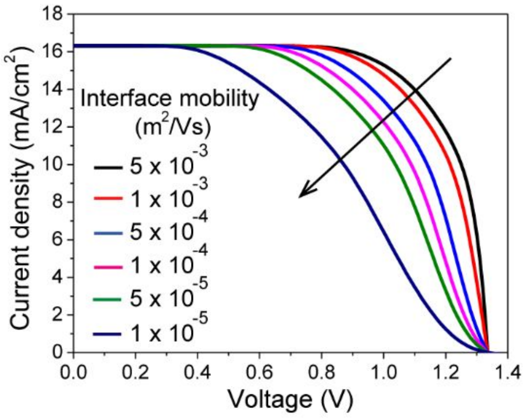

Figure S4. Simulated *JV* curves of the opaque solar cell for different carrier mobilities in C_{60} layer at perovskite/ETL interface (we considered the opaque solar cell as reference).

Table S1. Refractive index and extinction coefficients of materials employed to simulate the devices.

$Cs_yFA_{1-y}Pb(I_xBr_{1-x})_3$ (Perovskite)			C_{60}		MgF_2		SnO_2		Al	
Wavelength (nm)	*n*	κ	*n*	κ	*n*	κ	*n*	κ	*n*	κ
300	1.8144	0.8547	2.3495	0.8102	1.3836	0.0041	2.3205	0.4186	0.27571	3.61265
301	1.8205	0.8652	2.3427	0.7921	1.3834	0.004	2.3189	0.4097	0.27754	3.62538
302	1.827	0.8755	2.3351	0.7747	1.3831	0.004	2.3173	0.4009	0.27937	3.63812
303	1.8338	0.8855	2.3269	0.758	1.3828	0.004	2.3155	0.3924	0.2812	3.65085
304	1.841	0.8952	2.318	0.742	1.3826	0.0039	2.3137	0.3842	0.28303	3.66359
305	1.8485	0.9047	2.3083	0.7268	1.3823	0.0039	2.3119	0.3761	0.28485	3.67632
306	1.8563	0.9139	2.2977	0.7135	1.3821	0.0039	2.3099	0.3684	0.28668	3.68906
307	1.8645	0.9229	2.2866	0.7012	1.3818	0.0038	2.3078	0.3609	0.28851	3.70179
308	1.8731	0.9316	2.2752	0.6899	1.3816	0.0038	2.3057	0.3536	0.29034	3.71453
309	1.882	0.94	2.2634	0.6796	1.3814	0.0038	2.3036	0.3465	0.29217	3.72726
310	1.8913	0.9482	2.2511	0.6703	1.3811	0.0038	2.3014	0.3397	0.294	3.74
311	1.9009	0.9561	2.2394	0.6618	1.3809	0.0037	2.2991	0.3331	0.29596	3.75263
312	1.9109	0.9638	2.2274	0.6543	1.3807	0.0037	2.2968	0.3266	0.29792	3.76525
313	1.9212	0.9711	2.2152	0.6479	1.3804	0.0037	2.2945	0.3204	0.29988	3.77788

314	1.9319	0.9783	2.2029	0.6424	1.3802	0.0037	2.2922	0.3143	0.30185	3.7905
315	1.9429	0.9852	2.1903	0.6379	1.38	0.0037	2.2899	0.3084	0.30381	3.80313
316	1.9543	0.9919	2.1769	0.6339	1.3798	0.0036	2.2875	0.3027	0.30577	3.81575
317	1.966	0.9982	2.1634	0.6313	1.3796	0.0036	2.2852	0.2971	0.30773	3.82838
318	1.9781	1.0042	2.15	0.6299	1.3794	0.0036	2.2828	0.2918	0.30969	3.84101
319	1.9904	1.0099	2.1364	0.6299	1.3792	0.0036	2.2804	0.2865	0.31165	3.85363
320	2.0031	1.0151	2.1229	0.6312	1.379	0.0036	2.278	0.2814	0.31361	3.86626
321	2.016	1.02	2.109	0.6347	1.3788	0.0035	2.2757	0.2765	0.31557	3.87888
322	2.0292	1.0245	2.0958	0.6398	1.3786	0.0035	2.2733	0.2717	0.31754	3.89151
323	2.0427	1.0285	2.0833	0.6464	1.3784	0.0035	2.2709	0.267	0.3195	3.90414
324	2.0564	1.0321	2.0715	0.6546	1.3782	0.0035	2.2686	0.2625	0.32146	3.91676
325	2.0702	1.0352	2.0604	0.6644	1.378	0.0035	2.2662	0.2581	0.32342	3.92939
326	2.0843	1.0378	2.0507	0.6765	1.3779	0.0035	2.2639	0.2538	0.32538	3.94201
327	2.0985	1.0399	2.0426	0.6897	1.3777	0.0034	2.2616	0.2496	0.3274	3.95461
328	2.1129	1.0415	2.0364	0.7041	1.3775	0.0034	2.2593	0.2456	0.32944	3.96718
329	2.1272	1.0426	2.032	0.7195	1.3773	0.0034	2.257	0.2416	0.33148	3.97976
330	2.1417	1.0432	2.0293	0.736	1.3772	0.0034	2.2547	0.2378	0.33352	3.99234
331	2.1562	1.0432	2.0301	0.7543	1.377	0.0034	2.2525	0.2341	0.33556	4.00491
332	2.1708	1.0427	2.0331	0.7722	1.3768	0.0034	2.2502	0.2304	0.3376	4.01749
333	2.1852	1.0416	2.0384	0.7899	1.3767	0.0033	2.248	0.2269	0.33964	4.03007
334	2.1996	1.04	2.0459	0.8073	1.3765	0.0033	2.2458	0.2234	0.34168	4.04264
335	2.214	1.0379	2.0557	0.8244	1.3764	0.0033	2.2436	0.22	0.34372	4.05522
336	2.2282	1.0353	2.0694	0.839	1.3762	0.0033	2.2414	0.2168	0.34577	4.0678
337	2.2422	1.0321	2.0843	0.8521	1.3761	0.0033	2.2393	0.2136	0.34781	4.08037
338	2.2561	1.0284	2.1007	0.8636	1.3759	0.0033	2.2372	0.2104	0.34985	4.09295
339	2.2697	1.0243	2.1184	0.8735	1.3758	0.0033	2.2351	0.2074	0.35189	4.10553
340	2.2831	1.0196	2.1374	0.8819	1.3756	0.0033	2.233	0.2044	0.35393	4.1181
341	2.2963	1.0145	2.1561	0.8867	1.3755	0.0033	2.2309	0.2016	0.35597	4.13068
342	2.3091	1.009	2.1748	0.89	1.3753	0.0032	2.2289	0.1987	0.35801	4.14326
343	2.3216	1.0031	2.1937	0.8918	1.3752	0.0032	2.2269	0.196	0.36005	4.15583
344	2.3339	0.9968	2.2127	0.8922	1.375	0.0032	2.2249	0.1933	0.36209	4.16841
345	2.3457	0.99	2.2318	0.891	1.3749	0.0032	2.2229	0.1907	0.36421	4.18091
346	2.3572	0.983	2.2495	0.8895	1.3748	0.0032	2.221	0.1881	0.36638	4.19335
347	2.3683	0.9756	2.267	0.887	1.3746	0.0032	2.219	0.1856	0.36855	4.20578
348	2.3791	0.968	2.2845	0.8836	1.3745	0.0032	2.2171	0.1832	0.37072	4.21822
349	2.3894	0.96	2.3019	0.8792	1.3744	0.0032	2.2152	0.1808	0.37289	4.23066
350	2.3993	0.9518	2.3193	0.874	1.3742	0.0032	2.2134	0.1784	0.37506	4.2431
351	2.4088	0.9434	2.3374	0.8688	1.3741	0.0032	2.2115	0.1762	0.37724	4.25553
352	2.4179	0.9348	2.3555	0.8625	1.374	0.0032	2.2097	0.1739	0.37941	4.26797
353	2.4265	0.9261	2.3736	0.855	1.3739	0.0032	2.2079	0.1718	0.38158	4.28041
354	2.4348	0.9172	2.3917	0.8464	1.3737	0.0031	2.2061	0.1696	0.38375	4.29285
355	2.4426	0.9082	2.4097	0.8366	1.3736	0.0031	2.2044	0.1676	0.38592	4.30528
356	2.45	0.8991	2.4286	0.8247	1.3735	0.0031	2.2026	0.1655	0.38809	4.31772
357	2.457	0.89	2.4468	0.8112	1.3734	0.0031	2.2009	0.1635	0.39027	4.33016
358	2.4636	0.8808	2.4641	0.7964	1.3733	0.0031	2.1992	0.1616	0.39244	4.3426
359	2.4698	0.8716	2.4807	0.78	1.3732	0.0031	2.1975	0.1597	0.39461	4.35503
360	2.4756	0.8623	2.4965	0.7622	1.373	0.0031	2.1959	0.1578	0.39678	4.36747
361	2.481	0.8531	2.5102	0.7417	1.3729	0.0031	2.1942	0.156	0.39895	4.37991
362	2.486	0.844	2.5222	0.7203	1.3728	0.0031	2.1926	0.1542	0.40112	4.39235
363	2.4907	0.8349	2.5326	0.6983	1.3727	0.0031	2.191	0.1524	0.4033	4.40478
364	2.4951	0.8258	2.5412	0.6754	1.3726	0.0031	2.1894	0.1507	0.40547	4.41722
365	2.4991	0.8168	2.5482	0.6517	1.3725	0.0031	2.1879	0.149	0.40768	4.42964
366	2.5028	0.8079	2.5527	0.6292	1.3724	0.0031	2.1863	0.1474	0.41001	4.44201
367	2.5062	0.7991	2.5552	0.6066	1.3723	0.0031	2.1848	0.1458	0.41233	4.45438
368	2.5093	0.7905	2.5558	0.5838	1.3722	0.0031	2.1833	0.1442	0.41466	4.46675
369	2.5121	0.7819	2.5544	0.5609	1.3721	0.0031	2.1818	0.1426	0.41698	4.47913
370	2.5146	0.7735	2.5511	0.5379	1.372	0.0031	2.1803	0.1411	0.41931	4.4915
371	2.5169	0.7652	2.5468	0.5174	1.3719	0.0031	2.1789	0.1396	0.42163	4.50387
372	2.5189	0.7571	2.5414	0.4979	1.3718	0.003	2.1774	0.1382	0.42396	4.51624
373	2.5208	0.7491	2.535	0.4794	1.3717	0.003	2.176	0.1367	0.42629	4.52861
374	2.5224	0.7413	2.5278	0.462	1.3716	0.003	2.1746	0.1353	0.42861	4.54098
375	2.5238	0.7336	2.5199	0.4456	1.3715	0.003	2.1732	0.1339	0.43094	4.55335
376	2.525	0.7261	2.5113	0.4301	1.3714	0.003	2.1719	0.1326	0.43326	4.56573
377	2.526	0.7187	2.5023	0.4157	1.3713	0.003	2.1705	0.1312	0.43559	4.5781
378	2.5269	0.7116	2.4928	0.4022	1.3712	0.003	2.1692	0.1299	0.43791	4.59047
379	2.5276	0.7045	2.483	0.3897	1.3711	0.003	2.1678	0.1287	0.44024	4.60284
380	2.5281	0.6977	2.4728	0.3782	1.371	0.003	2.1665	0.1274	0.44256	4.61521
381	2.5286	0.691	2.4623	0.3677	1.3709	0.003	2.1652	0.1262	0.44489	4.62758
382	2.5288	0.6845	2.4518	0.3581	1.3708	0.003	2.164	0.125	0.44721	4.63996
383	2.529	0.6782	2.4412	0.3496	1.3708	0.003	2.1627	0.1238	0.44954	4.65233

384	2.5291	0.672	2.4307	0.3419	1.3707	0.003	2.1615	0.1226	0.45186	4.6647
385	2.5291	0.666	2.4203	0.3351	1.3706	0.003	2.1602	0.1215	0.45419	4.67707
386	2.529	0.6601	2.4102	0.3291	1.3705	0.003	2.159	0.1203	0.45651	4.68944
387	2.5288	0.6544	2.4003	0.3238	1.3704	0.003	2.1578	0.1192	0.45884	4.70181
388	2.5285	0.6489	2.3906	0.3191	1.3703	0.003	2.1566	0.1181	0.46122	4.71412
389	2.5282	0.6435	2.3813	0.315	1.3703	0.003	2.1554	0.1171	0.46366	4.72635
390	2.5278	0.6383	2.3723	0.3115	1.3702	0.003	2.1543	0.116	0.4661	4.73858
391	2.5274	0.6332	2.3636	0.3084	1.3701	0.003	2.1531	0.115	0.46854	4.75081
392	2.527	0.6283	2.3553	0.3058	1.37	0.003	2.152	0.114	0.47097	4.76305
393	2.5265	0.6235	2.3473	0.3035	1.3699	0.003	2.1509	0.113	0.47341	4.77528
394	2.5259	0.6189	2.3396	0.3015	1.3699	0.003	2.1498	0.112	0.47585	4.78751
395	2.5253	0.6144	2.3323	0.2997	1.3698	0.003	2.1487	0.111	0.47829	4.79974
396	2.5247	0.61	2.3252	0.2982	1.3697	0.003	2.1476	0.1101	0.48073	4.81197
397	2.5241	0.6057	2.3183	0.2969	1.3696	0.003	2.1465	0.1091	0.48317	4.82421
398	2.5235	0.6016	2.3116	0.2958	1.3696	0.003	2.1454	0.1082	0.48561	4.83644
399	2.5229	0.5976	2.3052	0.2949	1.3695	0.003	2.1444	0.1073	0.48805	4.84867
400	2.5223	0.5937	2.2989	0.2942	1.3694	0.003	2.1434	0.1064	0.49048	4.8609
401	2.5216	0.59	2.2929	0.2937	1.3693	0.003	2.1423	0.1055	0.49292	4.87314
402	2.521	0.5863	2.287	0.2935	1.3693	0.003	2.1413	0.1047	0.49536	4.88537
403	2.5204	0.5828	2.2814	0.2934	1.3692	0.003	2.1403	0.1038	0.4978	4.8976
404	2.5198	0.5793	2.2759	0.2934	1.3691	0.003	2.1393	0.103	0.50024	4.90983
405	2.5192	0.576	2.2706	0.2937	1.3691	0.003	2.1383	0.1021	0.50268	4.92206
406	2.5186	0.5727	2.2656	0.2941	1.369	0.003	2.1374	0.1013	0.50512	4.9343
407	2.5181	0.5696	2.2607	0.2947	1.3689	0.003	2.1364	0.1005	0.50755	4.94653
408	2.5175	0.5665	2.256	0.2954	1.3689	0.003	2.1355	0.0997	0.50999	4.95876
409	2.517	0.5635	2.2515	0.2963	1.3688	0.003	2.1345	0.099	0.51243	4.97099
410	2.5165	0.5606	2.2472	0.2973	1.3687	0.003	2.1336	0.0982	0.51487	4.98323
411	2.5161	0.5578	2.2431	0.2985	1.3687	0.003	2.1327	0.0975	0.51731	4.99546
412	2.5156	0.5551	2.2392	0.2997	1.3686	0.003	2.1318	0.0967	0.51975	5.00769
413	2.5152	0.5524	2.2355	0.3011	1.3685	0.003	2.1309	0.096	0.52219	5.01992
414	2.5148	0.5498	2.232	0.3026	1.3685	0.003	2.13	0.0953	0.52469	5.03215
415	2.5145	0.5473	2.2287	0.3043	1.3684	0.003	2.1291	0.0946	0.52723	5.04438
416	2.5142	0.5448	2.2255	0.306	1.3683	0.003	2.1282	0.0939	0.52977	5.05661
417	2.5139	0.5424	2.2226	0.3078	1.3683	0.003	2.1273	0.0932	0.53231	5.06883
418	2.5137	0.5401	2.2199	0.3097	1.3682	0.003	2.1265	0.0925	0.53485	5.08106
419	2.5135	0.5378	2.2174	0.3117	1.3682	0.003	2.1256	0.0918	0.5374	5.09329
420	2.5133	0.5355	2.2151	0.3137	1.3681	0.003	2.1248	0.0912	0.53994	5.10552
421	2.5132	0.5333	2.213	0.3158	1.368	0.003	2.124	0.0905	0.54248	5.11774
422	2.5131	0.5312	2.2112	0.318	1.368	0.003	2.1232	0.0899	0.54502	5.12997
423	2.513	0.5291	2.2095	0.3202	1.3679	0.003	2.1224	0.0892	0.54756	5.1422
424	2.513	0.527	2.2081	0.3225	1.3679	0.003	2.1216	0.0886	0.5501	5.15443
425	2.5131	0.525	2.2069	0.3248	1.3678	0.003	2.1208	0.088	0.55264	5.16665
426	2.5131	0.523	2.2059	0.3271	1.3677	0.003	2.12	0.0874	0.55518	5.17888
427	2.5132	0.521	2.2052	0.3295	1.3677	0.003	2.1192	0.0868	0.55772	5.19111
428	2.5134	0.5191	2.2046	0.3318	1.3676	0.003	2.1184	0.0862	0.56026	5.20334
429	2.5136	0.5172	2.2043	0.3341	1.3676	0.003	2.1177	0.0856	0.5628	5.21556
430	2.5138	0.5153	2.2042	0.3363	1.3675	0.003	2.1169	0.0851	0.56534	5.22779
431	2.514	0.5134	2.2043	0.3386	1.3675	0.003	2.1162	0.0845	0.56788	5.24002
432	2.5143	0.5116	2.2045	0.3408	1.3674	0.003	2.1154	0.0839	0.57042	5.25225
433	2.5147	0.5097	2.205	0.3429	1.3674	0.003	2.1147	0.0834	0.57296	5.26447
434	2.515	0.5079	2.2057	0.345	1.3673	0.003	2.114	0.0828	0.5755	5.2767
435	2.5155	0.5061	2.2066	0.347	1.3673	0.003	2.1132	0.0823	0.57804	5.28893
436	2.5159	0.5043	2.2077	0.3489	1.3672	0.0031	2.1125	0.0818	0.58058	5.30115
437	2.5164	0.5025	2.2089	0.3507	1.3671	0.0031	2.1118	0.0812	0.58312	5.31338
438	2.5169	0.5008	2.2103	0.3524	1.3671	0.0031	2.1111	0.0807	0.58566	5.32561
439	2.5175	0.499	2.2119	0.354	1.367	0.0031	2.1104	0.0802	0.5882	5.33784
440	2.5181	0.4972	2.2136	0.3556	1.367	0.0031	2.1098	0.0797	0.59074	5.35006
441	2.5187	0.4954	2.2156	0.3569	1.3669	0.0031	2.1091	0.0792	0.59328	5.36229
442	2.5193	0.4937	2.2176	0.3582	1.3669	0.0031	2.1084	0.0787	0.59582	5.37452
443	2.52	0.4919	2.2198	0.3593	1.3668	0.0031	2.1078	0.0782	0.59841	5.38674
444	2.5208	0.4901	2.2221	0.3603	1.3668	0.0031	2.1071	0.0777	0.60125	5.39892
445	2.5215	0.4883	2.2245	0.3611	1.3667	0.0031	2.1064	0.0773	0.6041	5.4111
446	2.5223	0.4865	2.2271	0.3618	1.3667	0.0031	2.1058	0.0768	0.60695	5.42329
447	2.5231	0.4847	2.2297	0.3623	1.3667	0.0031	2.1052	0.0763	0.6098	5.43547
448	2.5239	0.4829	2.2325	0.3626	1.3666	0.0031	2.1045	0.0759	0.61264	5.44765
449	2.5248	0.4811	2.2353	0.3627	1.3666	0.0031	2.1039	0.0754	0.61549	5.45983
450	2.5257	0.4792	2.2381	0.3627	1.3665	0.0031	2.1033	0.075	0.61834	5.47202
451	2.5266	0.4774	2.2411	0.3625	1.3665	0.0031	2.1027	0.0745	0.62119	5.4842
452	2.5275	0.4755	2.244	0.3621	1.3664	0.0031	2.1021	0.0741	0.62403	5.49638
453	2.5284	0.4736	2.247	0.3616	1.3664	0.0031	2.1014	0.0737	0.62688	5.50856

454	2.5294	0.4717	2.25	0.3608	1.3663	0.0031	2.1009	0.0732	0.62973	5.52075
455	2.5304	0.4698	2.2529	0.3599	1.3663	0.0031	2.1003	0.0728	0.63258	5.53293
456	2.5314	0.4678	2.2559	0.3588	1.3662	0.0031	2.0997	0.0724	0.63542	5.54511
457	2.5324	0.4658	2.2587	0.3575	1.3662	0.0031	2.0991	0.072	0.63827	5.55729
458	2.5334	0.4638	2.2616	0.3561	1.3662	0.0031	2.0985	0.0716	0.64112	5.56947
459	2.5345	0.4618	2.2644	0.3546	1.3661	0.0031	2.0979	0.0712	0.64397	5.58166
460	2.5355	0.4598	2.2671	0.353	1.3661	0.0031	2.0974	0.0708	0.64681	5.59384
461	2.5366	0.4577	2.2698	0.3512	1.366	0.0031	2.0968	0.0704	0.64966	5.60602
462	2.5377	0.4556	2.2724	0.3493	1.366	0.0031	2.0963	0.07	0.65251	5.6182
463	2.5388	0.4535	2.2749	0.3474	1.3659	0.0031	2.0957	0.0696	0.65536	5.63039
464	2.5399	0.4513	2.2774	0.3453	1.3659	0.0031	2.0952	0.0692	0.6582	5.64257
465	2.5409	0.4492	2.2798	0.3432	1.3659	0.0031	2.0946	0.0689	0.66105	5.65475
466	2.542	0.4469	2.2821	0.3409	1.3658	0.0032	2.0941	0.0685	0.6639	5.66693
467	2.5431	0.4447	2.2843	0.3386	1.3658	0.0032	2.0936	0.0681	0.66674	5.67911
468	2.5442	0.4425	2.2865	0.3363	1.3657	0.0032	2.093	0.0678	0.66959	5.6913
469	2.5453	0.4402	2.2886	0.3339	1.3657	0.0032	2.0925	0.0674	0.67244	5.70348
470	2.5464	0.4378	2.2907	0.3314	1.3657	0.0032	2.092	0.0671	0.67529	5.71566
471	2.5475	0.4355	2.2926	0.3289	1.3656	0.0032	2.0915	0.0667	0.67813	5.72784
472	2.5486	0.4331	2.2945	0.3264	1.3656	0.0032	2.091	0.0664	0.68098	5.74003
473	2.5496	0.4307	2.2963	0.3238	1.3655	0.0032	2.0905	0.066	0.68383	5.75221
474	2.5507	0.4282	2.2981	0.3211	1.3655	0.0032	2.09	0.0657	0.68668	5.76439
475	2.5517	0.4258	2.2998	0.3185	1.3655	0.0032	2.0895	0.0653	0.68952	5.77657
476	2.5528	0.4233	2.3014	0.3158	1.3654	0.0032	2.089	0.065	0.69237	5.78875
477	2.5538	0.4208	2.303	0.3132	1.3654	0.0032	2.0885	0.0647	0.69525	5.80093
478	2.5548	0.4182	2.3044	0.3105	1.3654	0.0032	2.088	0.0643	0.69855	5.81309
479	2.5558	0.4156	2.3059	0.3078	1.3653	0.0032	2.0875	0.064	0.70185	5.82524
480	2.5568	0.413	2.3073	0.3051	1.3653	0.0032	2.087	0.0637	0.70514	5.83739
481	2.5577	0.4104	2.3086	0.3024	1.3652	0.0032	2.0866	0.0634	0.70844	5.84955
482	2.5586	0.4077	2.3099	0.2997	1.3652	0.0032	2.0861	0.0631	0.71173	5.8617
483	2.5595	0.405	2.3112	0.297	1.3652	0.0032	2.0856	0.0628	0.71503	5.87385
484	2.5604	0.4023	2.3124	0.2943	1.3651	0.0032	2.0852	0.0624	0.71833	5.88601
485	2.5613	0.3995	2.3136	0.2916	1.3651	0.0032	2.0847	0.0621	0.72162	5.89816
486	2.5621	0.3968	2.3147	0.289	1.3651	0.0032	2.0843	0.0618	0.72492	5.91031
487	2.563	0.394	2.3159	0.2864	1.365	0.0032	2.0838	0.0615	0.72821	5.92246
488	2.5637	0.3911	2.317	0.2838	1.365	0.0033	2.0834	0.0612	0.73151	5.93462
489	2.5645	0.3883	2.3182	0.2812	1.365	0.0033	2.0829	0.061	0.73481	5.94677
490	2.5652	0.3854	2.3193	0.2786	1.3649	0.0033	2.0825	0.0607	0.7381	5.95892
491	2.5659	0.3826	2.3205	0.2761	1.3649	0.0033	2.0821	0.0604	0.7414	5.97108
492	2.5666	0.3797	2.3218	0.2735	1.3649	0.0033	2.0816	0.0601	0.7447	5.98323
493	2.5672	0.3767	2.3231	0.2709	1.3648	0.0033	2.0812	0.0598	0.74799	5.99538
494	2.5678	0.3738	2.3244	0.2683	1.3648	0.0033	2.0808	0.0595	0.75129	6.00753
495	2.5683	0.3709	2.3258	0.2657	1.3648	0.0033	2.0804	0.0593	0.75458	6.01969
496	2.5689	0.3679	2.3273	0.2629	1.3647	0.0033	2.08	0.059	0.75788	6.03184
497	2.5694	0.3649	2.3287	0.2601	1.3647	0.0033	2.0796	0.0587	0.76118	6.04399
498	2.5698	0.3619	2.3303	0.2573	1.3647	0.0033	2.0791	0.0584	0.76447	6.05615
499	2.5702	0.3589	2.3318	0.2543	1.3646	0.0033	2.0787	0.0582	0.76777	6.0683
500	2.5706	0.3558	2.3334	0.2512	1.3646	0.0033	2.0783	0.0579	0.77106	6.08045
501	2.571	0.3528	2.3349	0.2481	1.3646	0.0033	2.0779	0.0577	0.77436	6.0926
502	2.5713	0.3498	2.3365	0.2448	1.3645	0.0033	2.0775	0.0574	0.77766	6.10476
503	2.5715	0.3467	2.3381	0.2413	1.3645	0.0033	2.0771	0.0571	0.78095	6.11691
504	2.5717	0.3437	2.3397	0.2378	1.3645	0.0033	2.0768	0.0569	0.78425	6.12906
505	2.5719	0.3406	2.3412	0.2341	1.3644	0.0033	2.0764	0.0566	0.78755	6.14122
506	2.5721	0.3375	2.3427	0.2302	1.3644	0.0033	2.076	0.0564	0.79084	6.15337
507	2.5722	0.3345	2.3442	0.2262	1.3644	0.0033	2.0756	0.0561	0.79414	6.16552
508	2.5722	0.3314	2.3456	0.222	1.3644	0.0034	2.0752	0.0559	0.79743	6.17767
509	2.5722	0.3283	2.3468	0.2176	1.3643	0.0034	2.0749	0.0557	0.80073	6.18983
510	2.5722	0.3252	2.348	0.2131	1.3643	0.0034	2.0745	0.0554	0.80403	6.20198
511	2.5722	0.3222	2.3489	0.2084	1.3643	0.0034	2.0741	0.0552	0.80732	6.21413
512	2.5721	0.3191	2.3497	0.2036	1.3642	0.0034	2.0737	0.0549	0.81062	6.22629
513	2.5719	0.316	2.3503	0.1986	1.3642	0.0034	2.0734	0.0547	0.81391	6.23844
514	2.5717	0.313	2.3506	0.1936	1.3642	0.0034	2.073	0.0545	0.81721	6.25059
515	2.5715	0.3099	2.3508	0.1885	1.3642	0.0034	2.0727	0.0543	0.82051	6.26275
516	2.5712	0.3069	2.3507	0.1834	1.3641	0.0034	2.0723	0.054	0.8238	6.2749
517	2.5709	0.3038	2.3504	0.1783	1.3641	0.0034	2.072	0.0538	0.82736	6.287
518	2.5706	0.3008	2.3499	0.1731	1.3641	0.0034	2.0716	0.0536	0.83145	6.29898
519	2.5702	0.2978	2.3492	0.168	1.364	0.0034	2.0713	0.0534	0.83554	6.31097
520	2.5697	0.2947	2.3482	0.1628	1.364	0.0034	2.0709	0.0531	0.83963	6.32295
521	2.5693	0.2917	2.347	0.1578	1.364	0.0034	2.0706	0.0529	0.84371	6.33494
522	2.5687	0.2888	2.3457	0.1527	1.364	0.0034	2.0702	0.0527	0.8478	6.34693
523	2.5682	0.2858	2.3441	0.1478	1.3639	0.0034	2.0699	0.0525	0.85189	6.35891

524	2.5676	0.2828	2.3423	0.143	1.3639	0.0034	2.0696	0.0523	0.85598	6.3709
525	2.567	0.2799	2.3403	0.1382	1.3639	0.0034	2.0692	0.0521	0.86006	6.38289
526	2.5663	0.277	2.3381	0.1336	1.3638	0.0035	2.0689	0.0519	0.86415	6.39487
527	2.5656	0.2741	2.3358	0.1291	1.3638	0.0035	2.0686	0.0516	0.86824	6.40686
528	2.5649	0.2712	2.3332	0.1248	1.3638	0.0035	2.0682	0.0514	0.87233	6.41885
529	2.5641	0.2683	2.3306	0.1206	1.3638	0.0035	2.0679	0.0512	0.87642	6.43083
530	2.5633	0.2655	2.3278	0.1166	1.3637	0.0035	2.0676	0.051	0.8805	6.44282
531	2.5624	0.2627	2.3249	0.1128	1.3637	0.0035	2.0673	0.0508	0.88459	6.45481
532	2.5616	0.2599	2.3219	0.1092	1.3637	0.0035	2.067	0.0506	0.88868	6.46679
533	2.5606	0.2571	2.3189	0.1058	1.3637	0.0035	2.0667	0.0504	0.89277	6.47878
534	2.5597	0.2543	2.3158	0.1026	1.3636	0.0035	2.0663	0.0502	0.89685	6.49077
535	2.5587	0.2516	2.3127	0.0996	1.3636	0.0035	2.066	0.0501	0.90094	6.50275
536	2.5577	0.2489	2.3095	0.0967	1.3636	0.0035	2.0657	0.0499	0.90503	6.51474
537	2.5567	0.2463	2.3064	0.094	1.3636	0.0035	2.0654	0.0497	0.90912	6.52672
538	2.5556	0.2436	2.3033	0.0914	1.3635	0.0035	2.0651	0.0495	0.91321	6.53871
539	2.5545	0.241	2.3002	0.089	1.3635	0.0035	2.0648	0.0493	0.91729	6.5507
540	2.5533	0.2384	2.2971	0.0867	1.3635	0.0035	2.0645	0.0491	0.92138	6.56268
541	2.5522	0.2358	2.294	0.0846	1.3635	0.0035	2.0642	0.0489	0.92547	6.57467
542	2.551	0.2333	2.291	0.0825	1.3634	0.0035	2.0639	0.0487	0.92956	6.58666
543	2.5498	0.2308	2.288	0.0806	1.3634	0.0036	2.0636	0.0486	0.93364	6.59864
544	2.5485	0.2283	2.285	0.0788	1.3634	0.0036	2.0634	0.0484	0.93773	6.61063
545	2.5473	0.2259	2.2821	0.0771	1.3634	0.0036	2.0631	0.0482	0.94182	6.62262
546	2.546	0.2235	2.2792	0.0755	1.3634	0.0036	2.0628	0.048	0.94591	6.6346
547	2.5446	0.2211	2.2764	0.074	1.3633	0.0036	2.0625	0.0478	0.94999	6.64659
548	2.5433	0.2187	2.2736	0.0726	1.3633	0.0036	2.0622	0.0477	0.95408	6.65858
549	2.5419	0.2164	2.2708	0.0713	1.3633	0.0036	2.0619	0.0475	0.95817	6.67056
550	2.5406	0.2141	2.2682	0.07	1.3633	0.0036	2.0617	0.0473	0.96226	6.68255
551	2.5391	0.2119	2.2655	0.0688	1.3632	0.0036	2.0614	0.0472	0.96635	6.69453
552	2.5377	0.2096	2.2629	0.0677	1.3632	0.0036	2.0611	0.047	0.97043	6.70652
553	2.5363	0.2074	2.2604	0.0666	1.3632	0.0036	2.0608	0.0468	0.97452	6.71851
554	2.5348	0.2053	2.2579	0.0656	1.3632	0.0036	2.0606	0.0466	0.97861	6.73049
555	2.5333	0.2032	2.2555	0.0646	1.3631	0.0036	2.0603	0.0465	0.9827	6.74248
556	2.5318	0.2011	2.2531	0.0637	1.3631	0.0036	2.06	0.0463	0.98678	6.75447
557	2.5303	0.199	2.2507	0.0628	1.3631	0.0036	2.0598	0.0462	0.99087	6.76645
558	2.5287	0.197	2.2484	0.0619	1.3631	0.0036	2.0595	0.046	0.99496	6.77844
559	2.5272	0.1951	2.2461	0.0611	1.3631	0.0037	2.0592	0.0458	0.99905	6.79043
560	2.5256	0.1931	2.2438	0.0603	1.363	0.0037	2.059	0.0457	1.00314	6.80241
561	2.5241	0.1912	2.2416	0.0596	1.363	0.0037	2.0587	0.0455	1.00722	6.8144
562	2.5225	0.1893	2.2394	0.0589	1.363	0.0037	2.0585	0.0454	1.01131	6.82639
563	2.5209	0.1875	2.2372	0.0582	1.363	0.0037	2.0582	0.0452	1.0154	6.83837
564	2.5192	0.1857	2.2351	0.0576	1.363	0.0037	2.058	0.045	1.01985	6.85008
565	2.5176	0.1839	2.233	0.057	1.3629	0.0037	2.0577	0.0449	1.02492	6.86131
566	2.516	0.1822	2.2309	0.0564	1.3629	0.0037	2.0575	0.0447	1.02999	6.87255
567	2.5143	0.1805	2.2288	0.0559	1.3629	0.0037	2.0572	0.0446	1.03507	6.88378
568	2.5126	0.1788	2.2268	0.0554	1.3629	0.0037	2.057	0.0444	1.04014	6.89501
569	2.511	0.1772	2.2248	0.055	1.3629	0.0037	2.0567	0.0443	1.04522	6.90624
570	2.5093	0.1756	2.2229	0.0546	1.3628	0.0037	2.0565	0.0441	1.05029	6.91747
571	2.5076	0.174	2.221	0.0542	1.3628	0.0037	2.0562	0.044	1.05536	6.9287
572	2.506	0.1725	2.2191	0.0539	1.3628	0.0037	2.056	0.0438	1.06044	6.93993
573	2.5043	0.171	2.2172	0.0535	1.3628	0.0037	2.0558	0.0437	1.06551	6.95116
574	2.5026	0.1696	2.2154	0.0533	1.3628	0.0038	2.0555	0.0436	1.07059	6.96239
575	2.5009	0.1682	2.2136	0.053	1.3627	0.0038	2.0553	0.0434	1.07566	6.97362
576	2.4992	0.1668	2.2118	0.0528	1.3627	0.0038	2.0551	0.0433	1.08074	6.98485
577	2.4974	0.1655	2.2101	0.0526	1.3627	0.0038	2.0548	0.0431	1.08581	6.99608
578	2.4957	0.1642	2.2084	0.0525	1.3627	0.0038	2.0546	0.043	1.09088	7.00731
579	2.494	0.1629	2.2068	0.0524	1.3627	0.0038	2.0544	0.0429	1.09596	7.01854
580	2.4923	0.1617	2.2052	0.0523	1.3626	0.0038	2.0541	0.0427	1.10103	7.02977
581	2.4906	0.1605	2.2036	0.0523	1.3626	0.0038	2.0539	0.0426	1.10611	7.041
582	2.4888	0.1593	2.2021	0.0522	1.3626	0.0038	2.0537	0.0424	1.11118	7.05224
583	2.4871	0.1582	2.2006	0.0522	1.3626	0.0038	2.0535	0.0423	1.11625	7.06347
584	2.4854	0.1571	2.1991	0.0523	1.3626	0.0038	2.0533	0.0422	1.12133	7.0747
585	2.4837	0.1561	2.1978	0.0523	1.3625	0.0038	2.053	0.042	1.1264	7.08593
586	2.482	0.1551	2.1964	0.0524	1.3625	0.0038	2.0528	0.0419	1.13148	7.09716
587	2.4803	0.1541	2.1951	0.0524	1.3625	0.0038	2.0526	0.0418	1.13655	7.10839
588	2.4786	0.1532	2.1939	0.0525	1.3625	0.0038	2.0524	0.0416	1.14163	7.11962
589	2.4769	0.1523	2.1927	0.0526	1.3625	0.0039	2.0522	0.0415	1.1467	7.13085
590	2.4752	0.1514	2.1916	0.0528	1.3625	0.0039	2.052	0.0414	1.15177	7.14208
591	2.4735	0.1506	2.1905	0.0529	1.3624	0.0039	2.0517	0.0413	1.15685	7.15331
592	2.4718	0.1498	2.1895	0.053	1.3624	0.0039	2.0515	0.0411	1.16192	7.16454
593	2.4701	0.1491	2.1885	0.0531	1.3624	0.0039	2.0513	0.041	1.167	7.17577

594	2.4684	0.1484	2.1876	0.0532	1.3624	0.0039	2.0511	0.0409	1.17207	7.187
595	2.4667	0.1477	2.1867	0.0533	1.3624	0.0039	2.0509	0.0407	1.17715	7.19823
596	2.4651	0.147	2.1858	0.0534	1.3623	0.0039	2.0507	0.0406	1.18222	7.20946
597	2.4634	0.1464	2.185	0.0535	1.3623	0.0039	2.0505	0.0405	1.18729	7.2207
598	2.4618	0.1459	2.1843	0.0536	1.3623	0.0039	2.0503	0.0404	1.19237	7.23193
599	2.4602	0.1453	2.1836	0.0536	1.3623	0.0039	2.0501	0.0403	1.19744	7.24316
600	2.4586	0.1449	2.1829	0.0537	1.3623	0.0039	2.0499	0.0401	1.20252	7.25439
601	2.457	0.1444	2.1823	0.0537	1.3623	0.0039	2.0497	0.04	1.20759	7.26562
602	2.4554	0.144	2.1817	0.0537	1.3622	0.0039	2.0495	0.0399	1.21266	7.27685
603	2.4538	0.1436	2.1811	0.0536	1.3622	0.0039	2.0493	0.0398	1.21774	7.28808
604	2.4523	0.1433	2.1806	0.0536	1.3622	0.004	2.0491	0.0397	1.22281	7.29931
605	2.4507	0.143	2.1801	0.0535	1.3622	0.004	2.0489	0.0395	1.22789	7.31054
606	2.4491	0.1427	2.1796	0.0534	1.3622	0.004	2.0487	0.0394	1.23296	7.32177
607	2.4476	0.1425	2.1792	0.0533	1.3622	0.004	2.0485	0.0393	1.23804	7.333
608	2.4461	0.1423	2.1788	0.0531	1.3621	0.004	2.0483	0.0392	1.24311	7.34423
609	2.4446	0.1422	2.1784	0.0529	1.3621	0.004	2.0481	0.0391	1.24818	7.35546
610	2.4431	0.1421	2.178	0.0526	1.3621	0.004	2.048	0.039	1.25326	7.36669
611	2.4417	0.142	2.1777	0.0523	1.3621	0.004	2.0478	0.0388	1.25833	7.37792
612	2.4403	0.142	2.1773	0.052	1.3621	0.004	2.0476	0.0387	1.26341	7.38915
613	2.4389	0.142	2.177	0.0516	1.3621	0.004	2.0474	0.0386	1.26848	7.40039
614	2.4375	0.142	2.1766	0.0513	1.362	0.004	2.0472	0.0385	1.27355	7.41162
615	2.4362	0.1421	2.1762	0.0508	1.362	0.004	2.047	0.0384	1.27863	7.42285
616	2.4349	0.1423	2.1759	0.0504	1.362	0.004	2.0468	0.0383	1.2837	7.43408
617	2.4336	0.1424	2.1755	0.0499	1.362	0.004	2.0467	0.0382	1.28878	7.44531
618	2.4324	0.1426	2.1752	0.0494	1.362	0.004	2.0465	0.0381	1.29385	7.45654
619	2.4312	0.1429	2.1748	0.0489	1.362	0.0041	2.0463	0.038	1.29893	7.46777
620	2.43	0.1432	2.1744	0.0483	1.362	0.0041	2.0461	0.0379	1.304	7.479
621	2.4288	0.1436	2.174	0.0478	1.3619	0.0041	2.046	0.0378	1.30964	7.48948
622	2.4276	0.144	2.1736	0.0472	1.3619	0.0041	2.0458	0.0377	1.31528	7.49996
623	2.4264	0.1445	2.1731	0.0466	1.3619	0.0041	2.0456	0.0375	1.32092	7.51044
624	2.4254	0.1449	2.1727	0.046	1.3619	0.0041	2.0454	0.0374	1.32655	7.52092
625	2.4243	0.1455	2.1722	0.0453	1.3619	0.0041	2.0453	0.0373	1.33219	7.5314
626	2.4234	0.146	2.1718	0.0447	1.3619	0.0041	2.0451	0.0372	1.33783	7.54188
627	2.4225	0.1466	2.1713	0.044	1.3619	0.0041	2.0449	0.0371	1.34347	7.55236
628	2.4217	0.1472	2.1708	0.0434	1.3618	0.0041	2.0447	0.037	1.34911	7.56285
629	2.4209	0.1478	2.1702	0.0427	1.3618	0.0041	2.0446	0.0369	1.35475	7.57333
630	2.4202	0.1485	2.1697	0.042	1.3618	0.0041	2.0444	0.0368	1.36039	7.58381
631	2.4195	0.1492	2.1692	0.0413	1.3618	0.0041	2.0442	0.0367	1.36603	7.59429
632	2.419	0.1499	2.1686	0.0406	1.3618	0.0041	2.0441	0.0366	1.37166	7.60477
633	2.4184	0.1507	2.168	0.0399	1.3618	0.0042	2.0439	0.0365	1.3773	7.61525
634	2.418	0.1514	2.1674	0.0392	1.3618	0.0042	2.0437	0.0364	1.38294	7.62573
635	2.4176	0.1523	2.1668	0.0386	1.3617	0.0042	2.0436	0.0363	1.38858	7.63621
636	2.4172	0.1531	2.1662	0.0379	1.3617	0.0042	2.0434	0.0362	1.39422	7.64669
637	2.417	0.1544	2.1655	0.0372	1.3617	0.0042	2.0433	0.0361	1.39986	7.65717
638	2.4168	0.1556	2.1649	0.0365	1.3617	0.0042	2.0431	0.036	1.4055	7.66765
639	2.4167	0.1568	2.1642	0.0358	1.3617	0.0042	2.0429	0.0359	1.41114	7.67813
640	2.4168	0.1579	2.1635	0.0351	1.3617	0.0042	2.0428	0.0359	1.41677	7.68861
641	2.4169	0.1591	2.1628	0.0345	1.3617	0.0042	2.0426	0.0358	1.42241	7.69909
642	2.4171	0.1601	2.1621	0.0338	1.3616	0.0042	2.0425	0.0357	1.42805	7.70957
643	2.4174	0.1612	2.1614	0.0332	1.3616	0.0042	2.0423	0.0356	1.43369	7.72005
644	2.4179	0.1622	2.1607	0.0325	1.3616	0.0042	2.0422	0.0355	1.43933	7.73054
645	2.4184	0.1631	2.16	0.0319	1.3616	0.0042	2.042	0.0354	1.44497	7.74102
646	2.419	0.1641	2.1592	0.0313	1.3616	0.0042	2.0418	0.0353	1.45061	7.7515
647	2.4197	0.165	2.1585	0.0307	1.3616	0.0043	2.0417	0.0352	1.45625	7.76198
648	2.4205	0.1658	2.1577	0.0301	1.3616	0.0043	2.0415	0.0351	1.46188	7.77246
649	2.4214	0.1666	2.157	0.0295	1.3615	0.0043	2.0414	0.035	1.46752	7.78294
650	2.4224	0.1674	2.1562	0.0289	1.3615	0.0043	2.0412	0.0349	1.47316	7.79342
651	2.4235	0.1682	2.1554	0.0283	1.3615	0.0043	2.0411	0.0348	1.4788	7.8039
652	2.4247	0.1689	2.1546	0.0278	1.3615	0.0043	2.0409	0.0348	1.48444	7.81438
653	2.4264	0.1696	2.1539	0.0272	1.3615	0.0043	2.0408	0.0347	1.49057	7.8249
654	2.4287	0.1703	2.1531	0.0267	1.3615	0.0043	2.0407	0.0346	1.49755	7.83549
655	2.431	0.1709	2.1523	0.0261	1.3615	0.0043	2.0405	0.0345	1.50453	7.84608
656	2.4333	0.1713	2.1515	0.0256	1.3615	0.0043	2.0404	0.0344	1.5115	7.85667
657	2.4356	0.1716	2.1507	0.0251	1.3614	0.0043	2.0402	0.0343	1.51848	7.86727
658	2.438	0.1718	2.15	0.0245	1.3614	0.0043	2.0401	0.0342	1.52546	7.87786
659	2.4403	0.1718	2.1492	0.024	1.3614	0.0043	2.0399	0.0341	1.53244	7.88845
660	2.4427	0.1717	2.1484	0.0236	1.3614	0.0044	2.0398	0.0341	1.53942	7.89904
661	2.4451	0.1714	2.1476	0.0231	1.3614	0.0044	2.0396	0.034	1.54639	7.90963
662	2.4475	0.171	2.1468	0.0226	1.3614	0.0044	2.0395	0.0339	1.55337	7.92022
663	2.4499	0.1705	2.146	0.0221	1.3614	0.0044	2.0394	0.0338	1.56035	7.93081

664	2.4524	0.1698	2.1452	0.0217	1.3614	0.0044	2.0392	0.0337	1.56733	7.9414
665	2.4548	0.169	2.1444	0.0212	1.3613	0.0044	2.0391	0.0336	1.57431	7.95199
666	2.4572	0.1681	2.1436	0.0208	1.3613	0.0044	2.039	0.0336	1.58128	7.96258
667	2.4597	0.167	2.1428	0.0203	1.3613	0.0044	2.0388	0.0335	1.58826	7.97318
668	2.4622	0.1658	2.142	0.0199	1.3613	0.0044	2.0387	0.0334	1.59524	7.98377
669	2.4647	0.1644	2.1412	0.0195	1.3613	0.0044	2.0385	0.0333	1.60222	7.99436
670	2.4672	0.163	2.1404	0.0191	1.3613	0.0044	2.0384	0.0332	1.6092	8.00495
671	2.4697	0.1603	2.1396	0.0187	1.3613	0.0044	2.0383	0.0332	1.61617	8.01554
672	2.472	0.1571	2.1388	0.0183	1.3613	0.0044	2.0381	0.0331	1.62315	8.02613
673	2.4743	0.1539	2.138	0.0179	1.3613	0.0044	2.038	0.033	1.63013	8.03672
674	2.4764	0.1506	2.1372	0.0175	1.3612	0.0045	2.0379	0.0329	1.63711	8.04731
675	2.4784	0.1473	2.1364	0.0171	1.3612	0.0045	2.0377	0.0328	1.64408	8.0579
676	2.4802	0.1439	2.1356	0.0168	1.3612	0.0045	2.0376	0.0328	1.65106	8.06849
677	2.482	0.1405	2.1348	0.0164	1.3612	0.0045	2.0375	0.0327	1.65804	8.07909
678	2.4836	0.1369	2.1341	0.0161	1.3612	0.0045	2.0374	0.0326	1.66502	8.08968
679	2.4851	0.1334	2.1333	0.0157	1.3612	0.0045	2.0372	0.0325	1.672	8.10027
680	2.4864	0.1297	2.1325	0.0154	1.3612	0.0045	2.0371	0.0325	1.67897	8.11086
681	2.4877	0.126	2.1317	0.0151	1.3612	0.0045	2.037	0.0324	1.68595	8.12145
682	2.4888	0.1223	2.1309	0.0148	1.3611	0.0045	2.0368	0.0323	1.69293	8.13204
683	2.4898	0.1184	2.1301	0.0145	1.3611	0.0045	2.0367	0.0322	1.69991	8.14263
684	2.4907	0.1145	2.1293	0.0142	1.3611	0.0045	2.0366	0.0321	1.70689	8.15322
685	2.4915	0.1106	2.1286	0.0139	1.3611	0.0045	2.0365	0.0321	1.71386	8.16381
686	2.4921	0.1066	2.1278	0.0136	1.3611	0.0045	2.0363	0.032	1.72084	8.1744
687	2.4926	0.1025	2.127	0.0133	1.3611	0.0046	2.0362	0.0319	1.72782	8.18499
688	2.493	0.0984	2.1263	0.013	1.3611	0.0046	2.0361	0.0319	1.7348	8.19559
689	2.4931	0.0942	2.1255	0.0128	1.3611	0.0046	2.036	0.0318	1.7421	8.20601
690	2.492	0.0904	2.1247	0.0125	1.3611	0.0046	2.0359	0.0317	1.75202	8.21509
691	2.4908	0.0866	2.124	0.0123	1.3611	0.0046	2.0357	0.0316	1.76194	8.22417
692	2.4895	0.0829	2.1232	0.012	1.361	0.0046	2.0356	0.0316	1.77186	8.23325
693	2.4881	0.0793	2.1225	0.0118	1.361	0.0046	2.0355	0.0315	1.78178	8.24233
694	2.4867	0.0758	2.1217	0.0116	1.361	0.0046	2.0354	0.0314	1.7917	8.25142
695	2.4851	0.0723	2.121	0.0114	1.361	0.0046	2.0353	0.0313	1.80162	8.2605
696	2.4835	0.0689	2.1202	0.0111	1.361	0.0046	2.0351	0.0313	1.81154	8.26958
697	2.4818	0.0655	2.1195	0.0109	1.361	0.0046	2.035	0.0312	1.82146	8.27866
698	2.48	0.0622	2.1188	0.0107	1.361	0.0046	2.0349	0.0311	1.83139	8.28774
699	2.4781	0.059	2.118	0.0105	1.361	0.0046	2.0348	0.0311	1.84131	8.29682
700	2.4761	0.0559	2.1173	0.0103	1.361	0.0046	2.0347	0.031	1.85123	8.3059
701	2.4741	0.0528	2.1166	0.0102	1.3609	0.0047	2.0346	0.0309	1.86115	8.31498
702	2.472	0.0498	2.1159	0.01	1.3609	0.0047	2.0344	0.0309	1.87107	8.32407
703	2.4697	0.0468	2.1152	0.0098	1.3609	0.0047	2.0343	0.0308	1.88099	8.33315
704	2.4674	0.0439	2.1145	0.0097	1.3609	0.0047	2.0342	0.0307	1.89091	8.34223
705	2.465	0.0411	2.1137	0.0095	1.3609	0.0047	2.0341	0.0307	1.90083	8.35131
706	2.4626	0.0384	2.113	0.0093	1.3609	0.0047	2.034	0.0306	1.91075	8.36039
707	2.46	0.0357	2.1123	0.0092	1.3609	0.0047	2.0339	0.0305	1.92067	8.36947
708	2.4574	0.0331	2.1117	0.009	1.3609	0.0047	2.0338	0.0305	1.93059	8.37855
709	2.4548	0.0308	2.111	0.0089	1.3609	0.0047	2.0337	0.0304	1.94051	8.38763
710	2.4523	0.029	2.1103	0.0088	1.3609	0.0047	2.0336	0.0303	1.95043	8.39672
711	2.4499	0.0273	2.1096	0.0087	1.3608	0.0047	2.0334	0.0303	1.96035	8.4058
712	2.4475	0.0256	2.1089	0.0085	1.3608	0.0047	2.0333	0.0302	1.97027	8.41488
713	2.445	0.024	2.1083	0.0084	1.3608	0.0047	2.0332	0.0301	1.98019	8.42396
714	2.4426	0.0224	2.1076	0.0083	1.3608	0.0048	2.0331	0.0301	1.99011	8.43304
715	2.4402	0.0209	2.1069	0.0082	1.3608	0.0048	2.033	0.03	2.00003	8.44212
716	2.4378	0.0195	2.1063	0.0081	1.3608	0.0048	2.0329	0.0299	2.00995	8.4512
717	2.4354	0.0181	2.1056	0.008	1.3608	0.0048	2.0328	0.0299	2.01987	8.46029
718	2.433	0.0169	2.105	0.0079	1.3608	0.0048	2.0327	0.0298	2.02979	8.46937
719	2.4306	0.0156	2.1043	0.0079	1.3608	0.0048	2.0326	0.0297	2.03971	8.47845
720	2.4282	0.0145	2.1037	0.0078	1.3608	0.0048	2.0325	0.0297	2.04963	8.48753
721	2.4258	0.0134	2.1031	0.0077	1.3608	0.0048	2.0324	0.0296	2.05955	8.49661
722	2.4235	0.0124	2.1025	0.0076	1.3607	0.0048	2.0323	0.0295	2.06947	8.50569
723	2.4211	0.0114	2.1019	0.0076	1.3607	0.0048	2.0322	0.0295	2.07939	8.51477
724	2.4187	0.0105	2.1012	0.0075	1.3607	0.0048	2.0321	0.0294	2.08931	8.52385
725	2.4164	0.0097	2.1006	0.0074	1.3607	0.0048	2.032	0.0294	2.09923	8.53294
726	2.414	0.0089	2.1	0.0074	1.3607	0.0048	2.0319	0.0293	2.10915	8.54202
727	2.4117	0.0082	2.0994	0.0073	1.3607	0.0049	2.0318	0.0292	2.11907	8.5511
728	2.4093	0.0076	2.0989	0.0073	1.3607	0.0049	2.0317	0.0292	2.12899	8.56018
729	2.407	0.007	2.0983	0.0072	1.3607	0.0049	2.0316	0.0291	2.13892	8.56926
730	2.4048	0.0065	2.0977	0.0072	1.3607	0.0049	2.0315	0.0291	2.14922	8.57331
731	2.4028	0.0059	2.0971	0.0072	1.3607	0.0049	2.0314	0.029	2.15979	8.57384
732	2.4007	0.0054	2.0966	0.0071	1.3607	0.0049	2.0313	0.0289	2.17037	8.57436
733	2.3987	0.005	2.096	0.0071	1.3606	0.0049	2.0312	0.0289	2.18094	8.57489

734	2.3968	0.0045	2.0955	0.0071	1.3606	0.0049	2.0311	0.0288	2.19151	8.57542
735	2.3948	0.0041	2.0949	0.007	1.3606	0.0049	2.031	0.0288	2.20208	8.57594
736	2.3929	0.0036	2.0944	0.007	1.3606	0.0049	2.0309	0.0287	2.21266	8.57647
737	2.391	0.0033	2.0938	0.007	1.3606	0.0049	2.0308	0.0286	2.22323	8.57699
738	2.3891	0.0029	2.0933	0.007	1.3606	0.0049	2.0307	0.0286	2.2338	8.57752
739	2.3872	0.0026	2.0928	0.0069	1.3606	0.0049	2.0306	0.0285	2.24438	8.57805
740	2.3854	0.0023	2.0923	0.0069	1.3606	0.005	2.0305	0.0285	2.25495	8.57857
741	2.3835	0.002	2.0918	0.0069	1.3606	0.005	2.0304	0.0284	2.26552	8.5791
742	2.3817	0.0017	2.0912	0.0069	1.3606	0.005	2.0303	0.0284	2.27609	8.57963
743	2.38	0.0015	2.0907	0.0069	1.3606	0.005	2.0302	0.0283	2.28667	8.58015
744	2.3782	0.0012	2.0902	0.0069	1.3606	0.005	2.0301	0.0282	2.29724	8.58068
745	2.3765	0.0011	2.0898	0.0069	1.3605	0.005	2.03	0.0282	2.30781	8.58121
746	2.3748	0.0009	2.0893	0.0068	1.3605	0.005	2.0299	0.0281	2.31839	8.58173
747	2.3731	0.0007	2.0888	0.0068	1.3605	0.005	2.0298	0.0281	2.32896	8.58226
748	2.3715	0.0006	2.0883	0.0068	1.3605	0.005	2.0297	0.028	2.33953	8.58279
749	2.3698	0.0005	2.0878	0.0068	1.3605	0.005	2.0296	0.028	2.3501	8.58331
750	2.3682	0.0005	2.0874	0.0068	1.3605	0.005	2.0296	0.0279	2.36068	8.58384
751	2.3666	0.0004	2.0869	0.0068	1.3605	0.005	2.0295	0.0279	2.37125	8.58437
752	2.3651	0.0004	2.0865	0.0068	1.3605	0.005	2.0294	0.0278	2.38182	8.58489
753	2.3636	0.0003	2.086	0.0068	1.3605	0.0051	2.0293	0.0277	2.3924	8.58542
754	2.3621	0.0002	2.0856	0.0068	1.3605	0.0051	2.0292	0.0277	2.40297	8.58594
755	2.3606	0.0002	2.0851	0.0067	1.3605	0.0051	2.0291	0.0276	2.41354	8.58647
756	2.3591	0.0001	2.0847	0.0067	1.3605	0.0051	2.029	0.0276	2.42411	8.587
757	2.3577	0	2.0843	0.0067	1.3604	0.0051	2.0289	0.0275	2.43469	8.58752
758	2.3563	0	2.0838	0.0067	1.3604	0.0051	2.0288	0.0275	2.44526	8.58805
759	2.3549	0	2.0834	0.0067	1.3604	0.0051	2.0288	0.0274	2.45583	8.58858
760	2.3535	0	2.083	0.0067	1.3604	0.0051	2.0287	0.0274	2.46641	8.5891
761	2.3521	0	2.0826	0.0067	1.3604	0.0051	2.0286	0.0273	2.47698	8.58963
762	2.3507	0	2.0822	0.0067	1.3604	0.0051	2.0285	0.0273	2.48755	8.59016
763	2.3494	0	2.0818	0.0067	1.3604	0.0051	2.0284	0.0272	2.49813	8.59068
764	2.348	0	2.0813	0.0066	1.3604	0.0051	2.0283	0.0272	2.5087	8.59121
765	2.3467	0	2.081	0.0066	1.3604	0.0051	2.0282	0.0271	2.51927	8.59174
766	2.3454	0	2.0805	0.0066	1.3604	0.0052	2.0282	0.0271	2.52984	8.59226
767	2.3441	0	2.0801	0.0066	1.3604	0.0052	2.0281	0.027	2.54042	8.59279
768	2.3429	0	2.0798	0.0066	1.3604	0.0052	2.028	0.0269	2.55099	8.59331
769	2.3416	0	2.0794	0.0066	1.3604	0.0052	2.0279	0.0269	2.56156	8.59384
770	2.3404	0	2.079	0.0065	1.3603	0.0052	2.0278	0.0268	2.57214	8.59437
771	2.3392	0	2.0786	0.0065	1.3603	0.0052	2.0277	0.0268	2.58271	8.59489
772	2.338	0	2.0782	0.0065	1.3603	0.0052	2.0277	0.0267	2.59328	8.59542
773	2.3368	0	2.0778	0.0065	1.3603	0.0052	2.0276	0.0267	2.60385	8.59595
774	2.3356	0	2.0774	0.0065	1.3603	0.0052	2.0275	0.0266	2.61443	8.59647
775	2.3345	0	2.0771	0.0064	1.3603	0.0052	2.0274	0.0266	2.625	8.597
776	2.3334	0	2.0767	0.0064	1.3603	0.0052	2.0273	0.0265	2.62732	8.59143
777	2.3322	0	2.0763	0.0064	1.3603	0.0052	2.0272	0.0265	2.62965	8.58585
778	2.3311	0	2.076	0.0064	1.3603	0.0052	2.0272	0.0264	2.63197	8.58028
779	2.33	0	2.0756	0.0064	1.3603	0.0053	2.0271	0.0264	2.63429	8.5747
780	2.3289	0	2.0752	0.0063	1.3603	0.0053	2.027	0.0264	2.63661	8.56913
781	2.3278	0	2.0749	0.0063	1.3603	0.0053	2.0269	0.0263	2.63894	8.56355
782	2.3268	0	2.0745	0.0063	1.3603	0.0053	2.0268	0.0263	2.64126	8.55798
783	2.3257	0	2.0741	0.0063	1.3603	0.0053	2.0268	0.0262	2.64358	8.55241
784	2.3246	0	2.0738	0.0062	1.3602	0.0053	2.0267	0.0262	2.6459	8.54683
785	2.3236	0	2.0734	0.0062	1.3602	0.0053	2.0266	0.0261	2.64823	8.54126
786	2.3226	0	2.0731	0.0062	1.3602	0.0053	2.0265	0.0261	2.65055	8.53568
787	2.3215		2.0727	0.0062	1.3602	0.0053	2.0265	0.026	2.65287	8.53011
788	2.3205		2.0723	0.0062	1.3602	0.0053	2.0264	0.026	2.65519	8.52454
789	2.3195		2.072	0.0061	1.3602	0.0053	2.0263	0.0259	2.65752	8.51896
790	2.3185		2.0717	0.0061	1.3602	0.0053	2.0262	0.0259	2.65984	8.51339
791	2.3176		2.0713	0.0061	1.3602	0.0053	2.0262	0.0258	2.66216	8.50781
792	2.3166		2.071	0.0061	1.3602	0.0054	2.0261	0.0258	2.66448	8.50224
793	2.3156		2.0706	0.006	1.3602	0.0054	2.026	0.0257	2.66681	8.49666
794	2.3147		2.0703	0.006	1.3602	0.0054	2.0259	0.0257	2.66913	8.49109
795	2.3137		2.0699	0.006	1.3602	0.0054	2.0259	0.0256	2.67145	8.48552
796	2.3128		2.0696	0.006	1.3602	0.0054	2.0258	0.0256	2.67377	8.47994
797	2.3119		2.0693	0.006	1.3602	0.0054	2.0257	0.0255	2.6761	8.47437
798	2.311		2.0689	0.0059	1.3602	0.0054	2.0256	0.0255	2.67842	8.46879
799	2.3101		2.0686	0.0059	1.3601	0.0054	2.0256	0.0255	2.68074	8.46322
800	2.3092		2.0683	0.0059	1.3601	0.0054	2.0255	0.0254	2.68306	8.45765

Table S1. Refractive indices and extinction coefficients of materials employed to simulate the devices (continuation).

	ITO		PTAA		Glass		ITO-r	
Wavelength (nm)	*n*	κ	*n*	κ	*n*	κ	*n*	κ
300	2.2813	0.00187	1.54252	0.13628	1.5522	0	2.34139	0.1216
301	2.2772	0.018603	1.53952	0.14087	1.55191	0	2.3345	0.11614
302	2.2726	0.018079	1.53673	0.14568	1.55162	0	2.32778	0.11091
303	2.2677	0.012044	1.53416	0.15073	1.55133	0	2.32124	0.10589
304	2.2625	0.015326	1.53185	0.15601	1.55105	0	2.31486	0.10108
305	2.2579	0.008774	1.52982	0.1615	1.55076	0	2.30863	0.09648
306	2.2539	0.0072714	1.5281	0.1672	1.55047	0	2.30257	0.09208
307	2.2475	0.0096037	1.52672	0.17309	1.55018	0	2.29665	0.08786
308	2.2415	0.0068349	1.52573	0.17914	1.54989	0	2.29088	0.08384
309	2.2361	0.016937	1.52514	0.18533	1.54961	0	2.28525	0.07999
310	2.2313	0.020463	1.52499	0.19163	1.54932	0	2.27975	0.07632
311	2.2263	0.024543	1.5253	0.19799	1.54903	0	2.27439	0.07282
312	2.2198	0.027014	1.52609	0.20438	1.54874	0	2.26916	0.06948
313	2.2134	0.022376	1.52739	0.21072	1.54845	0	2.26405	0.06629
314	2.2071	0.02211	1.5292	0.21698	1.54819	0	2.25906	0.06326
315	2.2033	0.024245	1.53153	0.22309	1.54788	0	2.25419	0.06037
316	2.1991	0.024293	1.53437	0.22897	1.54759	0	2.24943	0.05761
317	2.1943	0.029667	1.53768	0.23458	1.54729	0	2.24478	0.05499
318	2.189	0.028785	1.54146	0.23984	1.547	0	2.24024	0.0525
319	2.1836	0.026074	1.54565	0.2447	1.54671	0	2.2358	0.05012
320	2.178	0.027697	1.5502	0.24909	1.54643	0	2.23147	0.04787
321	2.1735	0.027027	1.55505	0.25296	1.54615	0	2.22723	0.04572
322	2.1694	0.025978	1.56013	0.2563	1.54587	0	2.22308	0.04369
323	2.1626	0.025679	1.56536	0.25905	1.54559	0	2.21903	0.04175
324	2.1567	0.021698	1.57064	0.26124	1.54532	0	2.21506	0.03991
325	2.1521	0.019034	1.57592	0.26284	1.54505	0	2.21118	0.03816
326	2.1471	0.02045	1.5811	0.26388	1.54479	0	2.20738	0.03651
327	2.1419	0.020627	1.5861	0.26439	1.54453	0	2.20367	0.03493
328	2.1357	0.022622	1.59085	0.26441	1.54427	0	2.20003	0.03344
329	2.1306	0.023577	1.59531	0.26398	1.54401	0	2.19647	0.03202
330	2.1262	0.022893	1.5994	0.26319	1.54375	0	2.19298	0.03068
331	2.1228	0.022661	1.60309	0.26208	1.5435	0	2.18957	0.0294
332	2.1181	0.020708	1.60636	0.26073	1.54325	0	2.18622	0.02819
333	2.1117	0.020224	1.60919	0.25921	1.54301	0	2.18294	0.02705
334	2.1074	0.020013	1.61157	0.25758	1.54276	0	2.17972	0.02596
335	2.1034	0.019556	1.6135	0.25593	1.54252	0	2.17657	0.02493
336	2.0996	0.02072	1.61499	0.25431	1.54228	0	2.17348	0.02396
337	2.0958	0.021017	1.61607	0.25278	1.54204	0	2.17045	0.02303
338	2.0919	0.019538	1.61677	0.2514	1.54181	0	2.16748	0.02215
339	2.0886	0.019201	1.6171	0.25023	1.54157	0	2.16456	0.02132
340	2.0856	0.018182	1.61712	0.24931	1.54134	0	2.1617	0.02054
341	2.0828	0.017667	1.61686	0.24867	1.54111	0	2.15888	0.01979
342	2.08	0.018114	1.61638	0.24836	1.54089	0	2.15612	0.01909
343	2.0766	0.017231	1.61571	0.2484	1.54067	0	2.15341	0.01842
344	2.0718	0.016527	1.61492	0.24882	1.54045	0	2.15075	0.01779
345	2.067	0.017471	1.61405	0.24965	1.54023	0	2.14813	0.01719
346	2.0623	0.016735	1.61315	0.25088	1.54001	0	2.14556	0.01662
347	2.0576	0.016918	1.6123	0.25253	1.5398	0	2.14303	0.01609
348	2.0533	0.016522	1.61155	0.2546	1.53959	0	2.14054	0.01558
349	2.0496	0.015512	1.61095	0.25708	1.53937	0	2.1381	0.0151
350	2.0479	0.015817	1.61058	0.25997	1.53916	0	2.13569	0.01464
351	2.0447	0.01492	1.6105	0.26323	1.53896	0	2.13333	0.01421
352	2.0381	0.014959	1.61077	0.26685	1.53875	0	2.131	0.0138
353	2.0359	0.014533	1.61144	0.27077	1.53855	0	2.1287	0.01342
354	2.0349	0.013847	1.61259	0.27495	1.53835	0	2.12644	0.01305
355	2.0314	0.013409	1.61426	0.27932	1.53815	0	2.12422	0.01271
356	2.028	0.012806	1.61649	0.28382	1.53796	0	2.12203	0.01238
357	2.0246	0.012533	1.61932	0.28835	1.53776	0	2.11987	0.01207
358	2.0224	0.012349	1.62279	0.29283	1.53757	0	2.11774	0.01178
359	2.0195	0.012155	1.62686	0.29715	1.53738	0	2.11564	0.0115
360	2.0154	0.011969	1.63158	0.30121	1.53719	0	2.11357	0.01124

361	2.0119	0.012039	1.63688	0.30492	1.53701	0	2.11153	0.011
362	2.0091	0.011581	1.64271	0.30815	1.53682	0	2.10952	0.01076
363	2.0086	0.011391	1.64901	0.31084	1.53664	0	2.10753	0.01054
364	2.0052	0.010411	1.65568	0.31288	1.53645	0	2.10557	0.01033
365	1.9994	0.0099474	1.66262	0.31422	1.53627	0	2.10363	0.01013
366	1.9968	0.0097086	1.66972	0.31481	1.53609	0	2.10172	0.00995
367	1.9946	0.0092788	1.67683	0.31464	1.53591	0	2.09983	0.00977
368	1.9932	0.009074	1.68384	0.31371	1.53573	0	2.09796	0.0096
369	1.9897	0.0082	1.69062	0.31204	1.53556	0	2.09612	0.00944
370	1.9864	0.0078682	1.69704	0.30969	1.53539	0	2.0943	0.00929
371	1.986	0.0077123	1.703	0.30675	1.53522	0	2.0925	0.00915
372	1.9843	0.0077311	1.70842	0.30329	1.53506	0	2.09071	0.00902
373	1.9817	0.0077597	1.71321	0.29941	1.53488	0	2.08895	0.00889
374	1.9806	0.0075086	1.71733	0.29524	1.53472	0	2.08721	0.00877
375	1.9791	0.0070569	1.72074	0.29087	1.53455	0	2.08548	0.00866
376	1.9768	0.0070063	1.72343	0.28645	1.53438	0	2.08378	0.00855
377	1.9748	0.006755	1.72539	0.28207	1.53422	0	2.08209	0.00845
378	1.973	0.0065038	1.72664	0.27785	1.53406	0	2.08041	0.00835
379	1.9712	0.0065226	1.72722	0.27391	1.53391	0	2.07876	0.00826
380	1.9691	0.00627	1.72715	0.27035	1.53375	0	2.07712	0.00818
381	1.9669	0.0057477	1.7265	0.26728	1.53359	0	2.07549	0.00809
382	1.9645	0.00575	1.72533	0.2648	1.53343	0	2.07388	0.00802
383	1.9635	0.005218	1.72372	0.263	1.53328	0	2.07228	0.00795
384	1.9644	0.00523	1.72175	0.26199	1.53312	0	2.0707	0.00788
385	1.9614	0.00481	1.71954	0.26184	1.53297	0	2.06913	0.00781
386	1.9587	0.0047046	1.7172	0.26268	1.53283	0	2.06757	0.00775
387	1.9599	0.00472	1.71489	0.26458	1.53268	0	2.06603	0.00769
388	1.9591	0.00445	1.71279	0.26762	1.53253	0	2.0645	0.00764
389	1.9572	0.0041482	1.71113	0.27188	1.53238	0	2.06298	0.00759
390	1.9562	0.00391	1.71018	0.27738	1.53224	0	2.06147	0.00754
391	1.9551	0.00364	1.71024	0.28411	1.53209	0	2.05997	0.00749
392	1.954	0.0031476	1.7117	0.29201	1.53195	0	2.05849	0.00745
393	1.9508	0.0031	1.71495	0.30085	1.53181	0	2.05701	0.00741
394	1.9477	0.00295	1.72036	0.31033	1.53167	0	2.05555	0.00737
395	1.9463	0.0026982	1.72832	0.31996	1.53153	0	2.05409	0.00733
396	1.946	0.00284	1.73909	0.32908	1.53139	0	2.05265	0.0073
397	1.9462	0.0027732	1.75269	0.33689	1.53125	0	2.05121	0.00726
398	1.9439	0.00257	1.76886	0.34255	1.53112	0	2.04978	0.00723
399	1.9431	0.00229	1.78705	0.34526	1.53099	0	2.04836	0.0072
400	1.9438	0.00229	1.80641	0.34441	1.53085	0	2.04695	0.00717
401	1.9428	0.00201	1.8259	0.33971	1.53072	0	2.04555	0.00715
402	1.9418	0.00202	1.84442	0.33136	1.53059	0	2.04416	0.00712
403	1.9412	0.00231	1.86107	0.31984	1.53045	0	2.04277	0.0071
404	1.9415	0.00232	1.87521	0.30592	1.53033	0	2.04139	0.00708
405	1.9419	0.00232	1.8865	0.29041	1.5302	0	2.04002	0.00706
406	1.9392	0.0022276	1.89491	0.27414	1.53007	0	2.03866	0.00704
407	1.9376	0.0023326	1.90065	0.25778	1.52994	0	2.0373	0.00702
408	1.9369	0.00205	1.90402	0.24184	1.52981	0	2.03595	0.007
409	1.9356	0.00205	1.90542	0.22667	1.52969	0	2.0346	0.00698
410	1.9347	0.0017688	1.90522	0.21248	1.52957	0	2.03326	0.00697
411	1.9349	0.00177	1.90376	0.19935	1.52945	0	2.03193	0.00695
412	1.9337	0.00148	1.90138	0.18731	1.52933	0	2.0306	0.00694
413	1.9324	0.0012743	1.89829	0.17634	1.52921	0	2.02928	0.00692
414	1.9334	0.00148	1.89471	0.16635	1.52908	0	2.02797	0.00691
415	1.9332	0.00149	1.8908	0.15728	1.52896	0	2.02666	0.0069
416	1.932	0.00179	1.88667	0.14904	1.52884	0	2.02535	0.00689
417	1.9321	0.0016962	1.88241	0.14156	1.52873	0	2.02405	0.00688
418	1.9323	0.0015	1.87811	0.13475	1.52862	0	2.02276	0.00687
419	1.9324	0.0012	1.8738	0.12854	1.5285	0	2.02147	0.00686
420	1.9317	0.0012006	1.86953	0.12287	1.52839	0	2.02018	0.00685
421	1.9309	0.00121	1.86531	0.11768	1.52827	0	2.0189	0.00684
422	1.9318	0.00121	1.86119	0.11292	1.52816	0	2.01762	0.00683
423	1.9312	0.00152	1.85716	0.10854	1.52804	0	2.01635	0.00682
424	1.9295	0.00152	1.85323	0.1045	1.52793	0	2.01508	0.00682
425	1.9306	0.00122	1.8494	0.10077	1.52782	0	2.01382	0.00681
426	1.931	0.00122	1.84568	0.0973	1.52772	0	2.01255	0.0068
427	1.9299	0.0012249	1.84207	0.09409	1.52761	0	2.0113	0.0068
428	1.9301	0.00123	1.83858	0.0911	1.5275	0	2.01004	0.00679
429	1.93	0.00123	1.83519	0.0883	1.5274	0	2.00879	0.00678
430	1.9258	0.0011517	1.83189	0.08569	1.52729	0	2.00754	0.00678

431	1.9256	0.00092697	1.82871	0.08324	1.52718	0	2.0063	0.00677
432	1.9285	0.00095649	1.82562	0.08094	1.52708	0	2.00506	0.00677
433	1.9274	0.00093127	1.82262	0.07878	1.52698	0	2.00382	0.00677
434	1.9255	0.00124	1.81972	0.07674	1.52687	0	2.00259	0.00676
435	1.9228	0.00125	1.8169	0.07481	1.52677	0	2.00136	0.00676
436	1.9251	0.00125	1.81417	0.073	1.52667	0	2.00013	0.00675
437	1.928	0.00125	1.81151	0.07128	1.52656	0	1.9989	0.00675
438	1.9281	0.00126	1.80893	0.06964	1.52647	0	1.99768	0.00675
439	1.9265	0.00126	1.80643	0.06808	1.52637	0	1.99646	0.00674
440	1.9236	0.00126	1.804	0.06661	1.52627	0	1.99524	0.00674
441	1.9263	0.0014432	1.80162	0.0652	1.52617	0	1.99402	0.00674
442	1.9283	0.0015838	1.79932	0.06386	1.52607	0	1.99281	0.00674
443	1.929	0.0014865	1.79708	0.06258	1.52598	0	1.99159	0.00673
444	1.9279	0.0015147	1.79489	0.06136	1.52588	0	1.99039	0.00673
445	1.9269	0.0015918	1.79277	0.06019	1.52578	0	1.98918	0.00673
446	1.9281	0.0016	1.79071	0.05907	1.52569	0	1.98797	0.00673
447	1.9296	0.0016188	1.78868	0.05798	1.5256	0	1.98677	0.00672
448	1.931	0.00193	1.78671	0.05695	1.52551	0	1.98557	0.00672
449	1.929	0.00193	1.7848	0.05596	1.52541	0	1.98437	0.00672
450	1.9292	0.0019385	1.78292	0.05501	1.52532	0	1.98317	0.00672
451	1.9323	0.00194	1.78109	0.05409	1.52523	0	1.98197	0.00672
452	1.9311	0.00194	1.77931	0.05321	1.52513	0	1.98078	0.00672
453	1.9296	0.0020535	1.77756	0.05235	1.52504	0	1.97959	0.00671
454	1.9295	0.00195	1.77586	0.05154	1.52496	0	1.9784	0.00671
455	1.9311	0.00196	1.77419	0.05074	1.52487	0	1.97721	0.00671
456	1.9334	0.00196	1.77255	0.04998	1.52478	0	1.97602	0.00671
457	1.9312	0.0021206	1.77096	0.04924	1.52469	0	1.97483	0.00671
458	1.9293	0.0023	1.7694	0.04852	1.52461	0	1.97365	0.00671
459	1.9278	0.0024165	1.76787	0.04783	1.52452	0	1.97246	0.00671
460	1.928	0.00264	1.76637	0.04716	1.52443	0	1.97128	0.00671
461	1.9286	0.0026426	1.76491	0.0465	1.52435	0	1.9701	0.00671
462	1.9296	0.00298	1.76347	0.04588	1.52426	0	1.96892	0.0067
463	1.9307	0.00299	1.76206	0.04527	1.52418	0	1.96774	0.0067
464	1.9317	0.0029912	1.76069	0.04467	1.5241	0	1.96656	0.0067
465	1.9309	0.003	1.75933	0.0441	1.52401	0	1.96538	0.0067
466	1.9292	0.00301	1.75801	0.04354	1.52393	0	1.96421	0.0067
467	1.9266	0.00301	1.75671	0.043	1.52385	0	1.96303	0.0067
468	1.9274	0.0033591	1.75543	0.04247	1.52376	0	1.96186	0.0067
469	1.9286	0.00366	1.75418	0.04196	1.52368	0	1.96069	0.0067
470	1.9295	0.0037082	1.75294	0.04146	1.5236	0	1.95952	0.0067
471	1.9299	0.0033779	1.75173	0.04097	1.52353	0	1.95835	0.0067
472	1.9298	0.00338	1.75055	0.0405	1.52345	0	1.95718	0.0067
473	1.9275	0.00339	1.74939	0.04003	1.52337	0	1.95601	0.0067
474	1.9257	0.0034	1.74824	0.03958	1.52329	0	1.95484	0.0067
475	1.9246	0.0034064	1.74712	0.03915	1.52322	0	1.95367	0.0067
476	1.9263	0.00375	1.74601	0.03872	1.52313	0	1.95251	0.0067
477	1.9262	0.00376	1.74492	0.0383	1.52306	0	1.95134	0.0067
478	1.9214	0.00377	1.74385	0.0379	1.52298	0	1.95018	0.0067
479	1.9198	0.00412	1.7428	0.0375	1.5229	0	1.94902	0.0067
480	1.9188	0.00413	1.74176	0.03711	1.52283	0	1.94785	0.0067
481	1.9146	0.00428	1.74075	0.03673	1.52275	0	1.94669	0.0067
482	1.9122	0.00449	1.73975	0.03636	1.52268	0	1.94553	0.00669
483	1.9115	0.0045	1.73876	0.036	1.5226	0	1.94437	0.00669
484	1.9122	0.00451	1.73779	0.03565	1.52253	0	1.94321	0.00669
485	1.9117	0.0046229	1.73684	0.0353	1.52246	0	1.94205	0.00669
486	1.909	0.00453	1.7359	0.03496	1.52238	0	1.94089	0.00669
487	1.9058	0.00489	1.73497	0.03463	1.52232	0	1.93973	0.00669
488	1.9026	0.00455	1.73406	0.03431	1.52225	0	1.93857	0.00669
489	1.8999	0.0046236	1.73316	0.03399	1.52217	0	1.93741	0.00669
490	1.8991	0.00492	1.73228	0.03368	1.5221	0	1.93626	0.00669
491	1.8996	0.00493	1.7314	0.03338	1.52203	0	1.9351	0.00669
492	1.8989	0.00494	1.73055	0.03308	1.52196	0	1.93394	0.00669
493	1.8971	0.00495	1.7297	0.03278	1.52189	0	1.93279	0.00669
494	1.8935	0.0053109	1.72886	0.03249	1.52182	0	1.93163	0.00669
495	1.8912	0.0053206	1.72804	0.03222	1.52176	0	1.93048	0.00669
496	1.8894	0.0053303	1.72723	0.03194	1.52169	0	1.92932	0.00669
497	1.8875	0.0057	1.72643	0.03167	1.52161	0	1.92817	0.00669
498	1.8866	0.00571	1.72564	0.03141	1.52155	0	1.92702	0.00669
499	1.8862	0.00572	1.72487	0.03115	1.52148	0	1.92586	0.00669
500	1.8839	0.0057397	1.7241	0.03089	1.52141	0	1.92471	0.00669

501	1.8833	0.0057497	1.72333	0.03064	1.52135	0	1.92356	0.00669
502	1.8853	0.0060982	1.72259	0.0304	1.52128	0	1.92241	0.00669
503	1.8833	0.00613	1.72185	0.03016	1.52121	0	1.92126	0.00669
504	1.8807	0.00614	1.72113	0.02992	1.52115	0	1.92011	0.00669
505	1.879	0.0061591	1.72041	0.02969	1.52109	0	1.91895	0.00669
506	1.8778	0.0061691	1.7197	0.02946	1.52103	0	1.9178	0.00669
507	1.8768	0.0065073	1.719	0.02923	1.52096	0	1.91665	0.00669
508	1.8726	0.0065591	1.7183	0.02902	1.5209	0	1.9155	0.00669
509	1.871	0.0065691	1.71762	0.02879	1.52084	0	1.91435	0.00669
510	1.873	0.00658	1.71695	0.02858	1.52077	0	1.91321	0.00669
511	1.8694	0.00659	1.71628	0.02838	1.52071	0	1.91206	0.00669
512	1.8655	0.0066088	1.71563	0.02817	1.52065	0	1.91091	0.00669
513	1.8641	0.00662	1.71498	0.02796	1.52058	0	1.90976	0.00669
514	1.8642	0.0069552	1.71434	0.02776	1.52052	0	1.90861	0.00669
515	1.865	0.0070188	1.7137	0.02757	1.52046	0	1.90746	0.00669
516	1.8641	0.0070288	1.71308	0.02738	1.5204	0	1.90631	0.00669
517	1.8634	0.00704	1.71246	0.02718	1.52033	0	1.90517	0.00669
518	1.8629	0.0070588	1.71185	0.027	1.52028	0	1.90402	0.00669
519	1.8602	0.00744	1.71124	0.02682	1.52022	0	1.90287	0.00669
520	1.8577	0.0074591	1.71064	0.02663	1.52016	0	1.90173	0.00669
521	1.8564	0.00747	1.71006	0.02645	1.5201	0	1.90058	0.00669
522	1.8557	0.0074891	1.70948	0.02628	1.52004	0	1.89943	0.00669
523	1.8553	0.00787	1.7089	0.02611	1.51998	0	1.89829	0.00669
524	1.855	0.0078894	1.70833	0.02593	1.51992	0	1.89714	0.00669
525	1.8533	0.0079	1.70776	0.02577	1.51987	0	1.89599	0.00669
526	1.8502	0.00754	1.70721	0.0256	1.51981	0	1.89485	0.00669
527	1.8492	0.0075597	1.70666	0.02544	1.51975	0	1.8937	0.00669
528	1.8481	0.00795	1.70612	0.02528	1.5197	0	1.89256	0.00669
529	1.8466	0.00796	1.70557	0.02512	1.51964	0	1.89141	0.00669
530	1.8459	0.00836	1.70504	0.02496	1.51959	0	1.89027	0.00669
531	1.8456	0.0083703	1.70452	0.02481	1.51953	0	1.88912	0.00669
532	1.8446	0.0083906	1.70399	0.02466	1.51947	0	1.88798	0.00669
533	1.8432	0.00841	1.70347	0.02451	1.51942	0	1.88683	0.00669
534	1.8415	0.0084209	1.70296	0.02436	1.51937	0	1.88569	0.00669
535	1.8412	0.00844	1.70246	0.02421	1.51931	0	1.88454	0.00669
536	1.8407	0.0085091	1.70196	0.02407	1.51925	0	1.8834	0.00669
537	1.8394	0.0088518	1.70147	0.02393	1.5192	0	1.88226	0.00669
538	1.8387	0.0088718	1.70098	0.02379	1.51914	0	1.88111	0.00669
539	1.8379	0.00889	1.70049	0.02365	1.51909	0	1.87997	0.00669
540	1.8349	0.0089024	1.70002	0.02351	1.51904	0	1.87882	0.00669
541	1.8335	0.00892	1.69954	0.02338	1.51898	0	1.87768	0.00669
542	1.8332	0.00894	1.69907	0.02325	1.51893	0	1.87654	0.00669
543	1.8304	0.0089533	1.6986	0.02311	1.51888	0	1.87539	0.00669
544	1.8286	0.00897	1.69814	0.02299	1.51883	0	1.87425	0.00669
545	1.8285	0.0089839	1.69769	0.02286	1.51878	0	1.87311	0.00669
546	1.8271	0.0093942	1.69724	0.02274	1.51873	0	1.87196	0.00669
547	1.8258	0.00941	1.69679	0.02261	1.51867	0	1.87082	0.00669
548	1.8257	0.0092291	1.69635	0.02249	1.51862	0	1.86968	0.00669
549	1.8248	0.00905	1.69591	0.02236	1.51857	0	1.86853	0.00669
550	1.8234	0.0094658	1.69548	0.02225	1.51852	0	1.86739	0.00669
551	1.8206	0.00948	1.69505	0.02213	1.51848	0	1.86625	0.00669
552	1.8189	0.0094967	1.69463	0.02201	1.51843	0	1.86511	0.00669
553	1.8187	0.009517	1.69421	0.0219	1.51837	0	1.86396	0.00669
554	1.8171	0.00953	1.69378	0.02178	1.51832	0	1.86282	0.00669
555	1.8158	0.0095479	1.69337	0.02167	1.51827	0	1.86168	0.00669
556	1.8165	0.0095685	1.69296	0.02155	1.51823	0	1.86053	0.00669
557	1.8155	0.0096285	1.69256	0.02145	1.51818	0	1.85939	0.00669
558	1.8136	0.0096	1.69215	0.02134	1.51813	0	1.85825	0.00669
559	1.8114	0.01002	1.69176	0.02123	1.51808	0	1.85711	0.00669
560	1.8108	0.01004	1.69136	0.02112	1.51803	0	1.85597	0.00669
561	1.812	0.010051	1.69097	0.02102	1.51798	0	1.85482	0.00669
562	1.8102	0.010072	1.69058	0.02091	1.51794	0	1.85368	0.00669
563	1.8083	0.010417	1.6902	0.02081	1.51789	0	1.85254	0.00669
564	1.8078	0.010209	1.68982	0.02071	1.51784	0	1.8514	0.00669
565	1.8067	0.010123	1.68944	0.02061	1.5178	0	1.85026	0.00669
566	1.8055	0.010144	1.68907	0.02051	1.51775	0	1.84911	0.00669
567	1.8041	0.010164	1.6887	0.02041	1.5177	0	1.84797	0.00669
568	1.8019	0.010585	1.68833	0.02031	1.51765	0	1.84683	0.00669
569	1.7989	0.010605	1.68797	0.02022	1.51761	0	1.84569	0.00669
570	1.7989	0.010378	1.68761	0.02012	1.51757	0	1.84455	0.00669

571	1.799	0.010237	1.68725	0.02003	1.51752	0	1.84341	0.00669
572	1.7984	0.01025	1.6869	0.01994	1.51748	0	1.84226	0.00669
573	1.7969	0.010593	1.68655	0.01985	1.51743	0	1.84112	0.00669
574	1.7952	0.010698	1.6862	0.01975	1.51739	0	1.83998	0.00669
575	1.7943	0.010719	1.68586	0.01966	1.51735	0	1.83884	0.00669
576	1.7942	0.01074	1.68552	0.01957	1.5173	0	1.8377	0.00669
577	1.7947	0.010751	1.68518	0.01949	1.51726	0	1.83656	0.00669
578	1.7931	0.010771	1.68484	0.0194	1.51721	0	1.83541	0.00669
579	1.792	0.010792	1.6845	0.01931	1.51717	0	1.83427	0.00669
580	1.792	0.010813	1.68417	0.01923	1.51712	0	1.83313	0.00669
581	1.7906	0.01083	1.68385	0.01914	1.51708	0	1.83199	0.00669
582	1.7887	0.01085	1.68352	0.01906	1.51703	0	1.83085	0.00669
583	1.7871	0.010865	1.6832	0.01898	1.51699	0	1.82971	0.00669
584	1.7866	0.010886	1.68288	0.01889	1.51695	0	1.82857	0.00669
585	1.787	0.010907	1.68256	0.01881	1.5169	0	1.82743	0.00669
586	1.7866	0.01092	1.68225	0.01873	1.51687	0	1.82629	0.00669
587	1.7855	0.01094	1.68194	0.01865	1.51682	0	1.82514	0.00669
588	1.7832	0.010959	1.68163	0.01857	1.51678	0	1.824	0.00669
589	1.7819	0.01098	1.68132	0.0185	1.51675	0	1.82286	0.00669
590	1.7809	0.011421	1.68102	0.01842	1.5167	0	1.82172	0.00669
591	1.7802	0.011012	1.68072	0.01834	1.51666	0	1.82058	0.00669
592	1.7791	0.011033	1.68041	0.01826	1.51662	0	1.81944	0.00669
593	1.7777	0.011053	1.68012	0.01819	1.51658	0	1.8183	0.00669
594	1.7766	0.011074	1.67983	0.01812	1.51653	0	1.81716	0.00669
595	1.776	0.01109	1.67953	0.01804	1.51649	0	1.81602	0.00669
596	1.7762	0.011536	1.67924	0.01796	1.51646	0	1.81488	0.00669
597	1.7747	0.011557	1.67896	0.01789	1.51641	0	1.81373	0.00669
598	1.7732	0.011578	1.67867	0.01782	1.51637	0	1.81259	0.00669
599	1.7727	0.011599	1.67839	0.01775	1.51634	0	1.81145	0.00669
600	1.7704	0.01161	1.67811	0.01768	1.5163	0	1.81031	0.00669
601	1.7673	0.011631	1.67783	0.01761	1.51625	0	1.80917	0.00669
602	1.7674	0.011652	1.67755	0.01754	1.51622	0	1.80803	0.00669
603	1.7671	0.011673	1.67727	0.01747	1.51618	0	1.80689	0.00669
604	1.7664	0.01169	1.67701	0.0174	1.51614	0	1.80575	0.00669
605	1.7643	0.01171	1.67673	0.01734	1.5161	0	1.80461	0.00669
606	1.7625	0.01173	1.67647	0.01727	1.51606	0	1.80347	0.00669
607	1.7618	0.011317	1.6762	0.01721	1.51602	0	1.80233	0.00669
608	1.7607	0.011768	1.67593	0.01714	1.51598	0	1.80119	0.00669
609	1.7592	0.01135	1.67567	0.01707	1.51595	0	1.80005	0.00669
610	1.7572	0.011371	1.67542	0.01701	1.51591	0	1.7989	0.00669
611	1.7561	0.01139	1.67515	0.01695	1.51587	0	1.79776	0.00669
612	1.756	0.01141	1.67489	0.01688	1.51584	0	1.79662	0.00669
613	1.7545	0.011424	1.67465	0.01682	1.5158	0	1.79548	0.00669
614	1.7539	0.011679	1.67439	0.01675	1.51576	0	1.79434	0.00669
615	1.7552	0.011467	1.67414	0.0167	1.51573	0	1.7932	0.00669
616	1.7532	0.011832	1.67389	0.01663	1.51569	0	1.79206	0.00669
617	1.7507	0.01194	1.67364	0.01657	1.51565	0	1.79092	0.00669
618	1.7532	0.01196	1.6734	0.01652	1.51562	0	1.78978	0.00669
619	1.753	0.011982	1.67315	0.01645	1.51558	0	1.78864	0.00669
620	1.7506	0.012003	1.67291	0.01639	1.51554	0	1.7875	0.00669
621	1.7481	0.01202	1.67267	0.01633	1.51551	0	1.78636	0.00669
622	1.7458	0.01204	1.67244	0.01628	1.51547	0	1.78522	0.00669
623	1.7439	0.01206	1.6722	0.01622	1.51543	0	1.78408	0.00669
624	1.7439	0.012078	1.67196	0.01616	1.51539	0	1.78294	0.00669
625	1.7441	0.012099	1.67172	0.0161	1.51536	0	1.7818	0.00669
626	1.7423	0.01212	1.67149	0.01605	1.51532	0	1.78066	0.00669
627	1.7403	0.01214	1.67127	0.01599	1.51529	0	1.77951	0.00669
628	1.738	0.012153	1.67104	0.01594	1.51526	0	1.77837	0.00669
629	1.7373	0.011725	1.67081	0.01588	1.51522	0	1.77723	0.00669
630	1.7367	0.011746	1.67059	0.01582	1.51519	0	1.77609	0.00669
631	1.7364	0.01176	1.67036	0.01577	1.51516	0	1.77495	0.00669
632	1.736	0.01178	1.67014	0.01572	1.51512	0	1.77381	0.00669
633	1.7355	0.012236	1.66992	0.01566	1.51508	0	1.77267	0.00669
634	1.7352	0.0122	1.6697	0.01561	1.51505	0	1.77153	0.00669
635	1.7342	0.012293	1.66948	0.01556	1.51502	0	1.77039	0.00669
636	1.733	0.01231	1.66926	0.01551	1.51498	0	1.76925	0.00669
637	1.7314	0.011876	1.66905	0.01545	1.51494	0	1.76811	0.00669
638	1.7293	0.01189	1.66884	0.0154	1.51491	0	1.76697	0.00669
639	1.7267	0.01191	1.66863	0.01535	1.51488	0	1.76583	0.00669
640	1.7245	0.011931	1.66842	0.0153	1.51484	0	1.76469	0.00669

641	1.7232	0.01195	1.66821	0.01526	1.51481	0	1.76355	0.00669
642	1.7241	0.011964	1.668	0.01521	1.51478	0	1.76241	0.00669
643	1.7234	0.011985	1.66779	0.01516	1.51474	0	1.76127	0.00669
644	1.722	0.012007	1.66759	0.01511	1.51471	0	1.76013	0.00669
645	1.72	0.01202	1.66738	0.01506	1.51468	0	1.75899	0.00669
646	1.7195	0.01204	1.66718	0.01501	1.51465	0	1.75785	0.00669
647	1.7203	0.01206	1.66698	0.01496	1.51462	0	1.75671	0.00669
648	1.7172	0.012543	1.66679	0.01492	1.51459	0	1.75557	0.00669
649	1.715	0.012095	1.66658	0.01487	1.51456	0	1.75443	0.00669
650	1.7151	0.011808	1.66639	0.01482	1.51452	0	1.75328	0.00669
651	1.7149	0.011668	1.6662	0.01477	1.51449	0	1.75214	0.00669
652	1.7142	0.01169	1.666	0.01472	1.51446	0	1.751	0.00669
653	1.712	0.011702	1.66581	0.01468	1.51443	0	1.74986	0.00669
654	1.711	0.012032	1.66561	0.01463	1.5144	0	1.74872	0.00669
655	1.7109	0.011967	1.66542	0.01459	1.51436	0	1.74758	0.00669
656	1.7102	0.011757	1.66523	0.01455	1.51433	0	1.74644	0.00669
657	1.7094	0.012188	1.66505	0.0145	1.5143	0	1.7453	0.00669
658	1.7084	0.012231	1.66486	0.01445	1.51427	0	1.74416	0.00669
659	1.7052	0.012282	1.66467	0.01441	1.51424	0	1.74302	0.00669
660	1.7025	0.012304	1.66449	0.01437	1.51421	0	1.74188	0.00669
661	1.7031	0.01232	1.6643	0.01432	1.51417	0	1.74074	0.00669
662	1.7033	0.011868	1.66413	0.01428	1.51414	0	1.7396	0.00669
663	1.7034	0.01188	1.66394	0.01424	1.51411	0	1.73846	0.00669
664	1.7013	0.011902	1.66376	0.0142	1.51408	0	1.73732	0.00669
665	1.7001	0.01192	1.66358	0.01415	1.51405	0	1.73618	0.00669
666	1.6998	0.012199	1.66341	0.01411	1.51402	0	1.73504	0.00669
667	1.6984	0.011957	1.66323	0.01407	1.51399	0	1.7339	0.00669
668	1.6972	0.01197	1.66305	0.01402	1.51396	0	1.73276	0.00669
669	1.6969	0.012411	1.66288	0.01399	1.51394	0	1.73162	0.00669
670	1.6958	0.01249	1.6627	0.01394	1.51391	0	1.73048	0.00669
671	1.6946	0.012505	1.66254	0.01391	1.51388	0	1.72934	0.00669
672	1.6946	0.012186	1.66236	0.01386	1.51385	0	1.7282	0.00669
673	1.6936	0.01206	1.66219	0.01382	1.51382	0	1.72706	0.00669
674	1.6914	0.012498	1.66202	0.01378	1.51379	0	1.72592	0.00669
675	1.6906	0.0121	1.66186	0.01375	1.51376	0	1.72478	0.00669
676	1.6892	0.012115	1.66169	0.01371	1.51373	0	1.72364	0.00669
677	1.6868	0.011647	1.66153	0.01367	1.5137	0	1.7225	0.00669
678	1.6862	0.011669	1.66136	0.01363	1.51367	0	1.72136	0.00669
679	1.686	0.011682	1.6612	0.01359	1.51364	0	1.72022	0.00669
680	1.6856	0.01219	1.66103	0.01355	1.51361	0	1.71907	0.00669
681	1.684	0.012206	1.66087	0.01352	1.51359	0	1.71793	0.00669
682	1.6815	0.01222	1.66071	0.01348	1.51356	0	1.71679	0.00669
683	1.6806	0.01175	1.66054	0.01344	1.51353	0	1.71565	0.00669
684	1.6802	0.01177	1.66039	0.0134	1.5135	0	1.71451	0.00669
685	1.6808	0.011785	1.66023	0.01336	1.51347	0	1.71337	0.00669
686	1.6794	0.0118	1.66007	0.01333	1.51344	0	1.71223	0.00669
687	1.6773	0.01182	1.65992	0.01329	1.51341	0	1.71109	0.00669
688	1.6742	0.012331	1.65976	0.01325	1.51339	0	1.70995	0.00669
689	1.6727	0.011854	1.65961	0.01322	1.51336	0	1.70881	0.00669
690	1.672	0.011376	1.65945	0.01318	1.51334	0	1.70767	0.00669
691	1.6715	0.01139	1.6593	0.01315	1.51331	0	1.70653	0.00669
692	1.6707	0.011411	1.65914	0.01311	1.51328	0	1.70539	0.00669
693	1.6697	0.011923	1.659	0.01308	1.51325	0	1.70425	0.00669
694	1.6687	0.01194	1.65885	0.01304	1.51323	0	1.70311	0.00669
695	1.6673	0.012458	1.65869	0.013	1.5132	0	1.70197	0.00669
696	1.6646	0.01247	1.65855	0.01297	1.51317	0	1.70083	0.00669
697	1.6632	0.011992	1.6584	0.01294	1.51315	0	1.69969	0.00669
698	1.6624	0.01201	1.65826	0.0129	1.51312	0	1.69855	0.00669
699	1.662	0.011527	1.65811	0.01287	1.51309	0	1.69741	0.00669
700	1.6615	0.01154	1.65797	0.01284	1.51306	0	1.69627	0.00669
701	1.6611	0.01156	1.65782	0.0128	1.51303	0	1.69513	0.00669
702	1.6606	0.011575	1.65768	0.01277	1.51301	0	1.69399	0.00669
703	1.6602	0.01159	1.65754	0.01273	1.51298	0	1.69285	0.00669
704	1.66	0.011116	1.6574	0.0127	1.51296	0	1.69171	0.00669
705	1.6579	0.011235	1.65725	0.01267	1.51293	0	1.69057	0.00669
706	1.6559	0.01164	1.65711	0.01263	1.5129	0	1.68943	0.00669
707	1.6567	0.011657	1.65697	0.0126	1.51288	0	1.68829	0.00669
708	1.6551	0.01167	1.65683	0.01257	1.51286	0	1.68715	0.00669
709	1.6521	0.01169	1.6567	0.01254	1.51284	0	1.68601	0.00669
710	1.6512	0.0112	1.65656	0.01251	1.51282	0	1.68487	0.00669

711	1.6506	0.011218	1.65642	0.01248	1.51279	0	1.68373	0.00669
712	1.6501	0.01174	1.65629	0.01244	1.51277	0	1.68259	0.00669
713	1.6492	0.01176	1.65615	0.01241	1.51275	0	1.68145	0.00669
714	1.6481	0.01126	1.65602	0.01238	1.51273	0	1.6803	0.00669
715	1.6466	0.011279	1.65589	0.01235	1.51271	0	1.67916	0.00669
716	1.6462	0.01078	1.65575	0.01232	1.51269	0	1.67802	0.00669
717	1.6461	0.01131	1.65562	0.01229	1.51267	0	1.67688	0.00669
718	1.6442	0.01081	1.65549	0.01226	1.51265	0	1.67574	0.00669
719	1.6427	0.01082	1.65536	0.01223	1.51262	0	1.6746	0.00669
720	1.6414	0.01084	1.65523	0.0122	1.5126	0	1.67346	0.00669
721	1.6409	0.011118	1.6551	0.01217	1.51258	0	1.67232	0.00669
722	1.6396	0.011388	1.65497	0.01214	1.51256	0	1.67118	0.00669
723	1.6366	0.011401	1.65484	0.01211	1.51254	0	1.67004	0.00669
724	1.6346	0.01142	1.65472	0.01208	1.51252	0	1.6689	0.00669
725	1.6328	0.01109	1.65459	0.01205	1.5125	0	1.66776	0.00669
726	1.631	0.01093	1.65447	0.01202	1.51248	0	1.66662	0.00669
727	1.6311	0.01147	1.65434	0.01199	1.51245	0	1.66548	0.00669
728	1.6328	0.012005	1.65422	0.01197	1.51243	0	1.66434	0.00669
729	1.6325	0.01202	1.6541	0.01194	1.51241	0	1.6632	0.00669
730	1.6317	0.011511	1.65397	0.01191	1.51239	0	1.66206	0.00669
731	1.6302	0.01153	1.65385	0.01188	1.51237	0	1.66092	0.00669
732	1.6286	0.01102	1.65373	0.01185	1.51235	0	1.65978	0.00669
733	1.6273	0.01104	1.65361	0.01182	1.51233	0	1.65864	0.00669
734	1.6266	0.010697	1.65348	0.0118	1.51231	0	1.6575	0.00669
735	1.6247	0.011066	1.65336	0.01177	1.51229	0	1.65636	0.00669
736	1.6224	0.01108	1.65325	0.01174	1.51226	0	1.65522	0.00669
737	1.6216	0.011093	1.65313	0.01171	1.51224	0	1.65408	0.00669
738	1.6204	0.01111	1.65301	0.01168	1.51222	0	1.65294	0.00669
739	1.6188	0.010599	1.65289	0.01166	1.5122	0	1.6518	0.00669
740	1.6164	0.01114	1.65278	0.01163	1.51218	0	1.65066	0.00669
741	1.6149	0.011155	1.65266	0.0116	1.51216	0	1.64952	0.00669
742	1.6156	0.01117	1.65254	0.01158	1.51214	0	1.64838	0.00669
743	1.6156	0.01119	1.65243	0.01156	1.51212	0	1.64724	0.00669
744	1.6152	0.0112	1.65231	0.01153	1.51209	0	1.6461	0.00669
745	1.6147	0.011218	1.6522	0.0115	1.51207	0	1.64496	0.00669
746	1.6134	0.011231	1.65209	0.01147	1.51205	0	1.64382	0.00669
747	1.6115	0.010962	1.65198	0.01145	1.51203	0	1.64268	0.00669
748	1.6106	0.010836	1.65186	0.01142	1.51201	0	1.64154	0.00669
749	1.6093	0.01128	1.65175	0.01139	1.51199	0	1.6404	0.00669
750	1.6074	0.01129	1.65164	0.01137	1.51197	0	1.63926	0.00669
751	1.6062	0.011308	1.65154	0.01134	1.51195	0	1.63811	0.00669
752	1.6055	0.010782	1.65143	0.01132	1.51192	0	1.63697	0.00669
753	1.606	0.010795	1.65131	0.0113	1.5119	0	1.63583	0.00669
754	1.6047	0.01081	1.6512	0.01127	1.51188	0	1.63469	0.00669
755	1.6026	0.010822	1.6511	0.01124	1.51186	0	1.63355	0.00669
756	1.6022	0.01084	1.65099	0.01122	1.51184	0	1.63241	0.00669
757	1.602	0.01085	1.65089	0.01119	1.51182	0	1.63127	0.00669
758	1.6019	0.01087	1.65078	0.01117	1.5118	0	1.63013	0.00669
759	1.598	0.01088	1.65067	0.01115	1.51178	0	1.62899	0.00669
760	1.5945	0.010899	1.65056	0.01112	1.51175	0	1.62785	0.00669
761	1.5927	0.01091	1.65046	0.01109	1.51173	0	1.62671	0.00669
762	1.5921	0.010927	1.65035	0.01107	1.51171	0	1.62557	0.00669
763	1.5915	0.01094	1.65025	0.01105	1.51169	0	1.62443	0.00669
764	1.5894	0.010752	1.65014	0.01103	1.51167	0	1.62329	0.00669
765	1.5885	0.01042	1.65005	0.011	1.51165	0	1.62215	0.00669
766	1.5885	0.010376	1.64995	0.01097	1.51163	0	1.62101	0.00669
767	1.5868	0.0099	1.64984	0.01095	1.51161	0	1.61987	0.00669
768	1.5857	0.00991	1.64974	0.01093	1.51158	0	1.61873	0.00669
769	1.5853	0.010472	1.64964	0.01091	1.51156	0	1.61759	0.00669
770	1.5848	0.01049	1.64954	0.01088	1.51154	0	1.61645	0.00669
771	1.5842	0.01105	1.64944	0.01086	1.51152	0	1.61531	0.00669
772	1.5839	0.01107	1.64934	0.01084	1.5115	0	1.61417	0.00669
773	1.5819	0.01108	1.64924	0.01082	1.51148	0	1.61303	0.00669
774	1.5791	0.010541	1.64914	0.01079	1.51146	0	1.61189	0.00669
775	1.5778	0.01	1.64905	0.01077	1.51144	0	1.61075	0.00669
776	1.5762	0.01001	1.64895	0.01075	1.51142	0	1.60961	0.00669
777	1.5744	0.01003	1.64885	0.01072	1.51139	0	1.60847	0.00669
778	1.5733	0.01004	1.64876	0.0107	1.51137	0	1.60733	0.00669
779	1.5731	0.010051	1.64866	0.01068	1.51135	0	1.60619	0.00669
780	1.5747	0.010065	1.64856	0.01066	1.51133	0	1.60505	0.00669

781	1.5756	0.01008	1.64847	0.01063	1.51131	0	1.60391	0.00669
782	1.5755	0.01009	1.64837	0.01061	1.51129	0	1.60277	0.00669
783	1.5717	0.010667	1.64828	0.01059	1.51127	0	1.60163	0.00669
784	1.5682	0.010718	1.64818	0.01057	1.51125	0	1.60049	0.00669
785	1.565	0.011255	1.64809	0.01055	1.51122	0	1.59935	0.00669
786	1.5665	0.01127	1.648	0.01052	1.5112	0	1.59821	0.00669
787	1.5655	0.01072	1.64791	0.01051	1.51118	0	1.59707	0.00669
788	1.5616	0.01017	1.64781	0.01048	1.51116	0	1.59593	0.00669
789	1.5598	0.00962	1.64772	0.01047	1.51114	0	1.59478	0.00669
790	1.5591	0.00963	1.64763	0.01044	1.51112	0	1.59364	0.00669
791	1.5601	0.00964	1.64754	0.01042	1.5111	0	1.5925	0.00669
792	1.5594	0.00965	1.64745	0.0104	1.51108	0	1.59136	0.00669
793	1.5579	0.010112	1.64736	0.01038	1.51105	0	1.59022	0.00669
794	1.5554	0.010679	1.64727	0.01036	1.51103	0	1.58908	0.00669
795	1.5548	0.01026	1.64718	0.01034	1.51101	0	1.58794	0.00669
796	1.5556	0.0097	1.64709	0.01032	1.51099	0	1.5868	0.00669
797	1.5556	0.00857	1.64701	0.0103	1.51097	0	1.58566	0.00669
798	1.5545	0.0091303	1.64692	0.01027	1.51095	0	1.58452	0.00669
799	1.5518	0.00881	1.64683	0.01026	1.51093	0	1.58338	0.00669
800	1.5489	0.00965	1.64674	0.01023	1.51091	0	1.58224	0.00669
